# Synthesis and structure–activity relationships of aryl fluorosulfate-based inhibitors as novel antitubercular agents

**DOI:** 10.1016/j.bmcl.2023.129596

**Published:** 2024-01-15

**Authors:** Baiyuan Yang, Paridhi Sukheja, Bo Qin, Gencheng Li, Grant A.L. Bare, Alessandro Cascioferro, Melissa S. Love, H. Michael Petrassi, K. Barry Sharpless, Case W. McNamara, Arnab K. Chatterjee

**Affiliations:** a Calibr, a Division of Scripps Research, La Jolla, CA 92037, USA; b Department of Chemistry, Scripps Research, La Jolla, CA 92037, USA

**Keywords:** Mycobacterium tuberculosis, Aryl fluorosulfate, Sulfur (VI) fluoride exchange, Structure–activity relationship studies, Anti-TB activity, Pharmacokinetic, SuFEx

## Abstract

To identify new compounds that can effectively inhibit *Mycobacterium tuberculosis* (Mtb), the causative agent of tuberculosis (TB), we screened, synthesized, and evaluated a series of novel aryl fluorosulfate derivatives for their *in vitro* inhibitory activity against Mtb. Compound **21b** exhibited an *in vitro* minimum inhibitory concentration (MIC) of 0.06 µM against Mtb, no cytotoxicity against both HEK293T and HepG2 mammalian cell lines, and had good *in vivo* mouse plasma exposure and lung concentration with a 20 mg/kg oral dose, which supports advanced development as a new chemical entity for TB treatment.

## Graphical abstract

**Figure:**
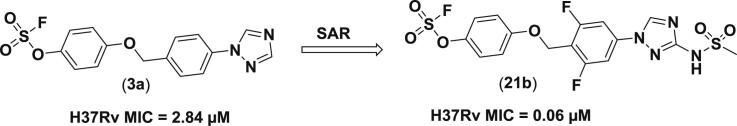

Before the COVID-19 global pandemic, tuberculosis (TB), caused by *Mycobacterium tuberculosis* (Mtb), was the leading cause of death due to a single infectious agent.^1^ People with TB need take multiple drug combo for standard 6 month, or even up to 2 years or longer for multi- and extensive-drug-resistant TB (MDR-TB/XDR-TB). The rising emergence of MDR-TB/XDR-TB is one of the most relevant public health problems worldwide.^1^, ^2^ For these reasons, there is an unmet medical need to deliver new drug candidates to expand the TB drug development pipeline and to shorten the treatment period. Click chemistry of sulfur (VI) fluoride exchange (SuFEx) groups, developed by Sharpless and coworkers,^3^ has been successfully introduced into many bioactive molecules in chemical biology and drug discovery, especially as a covalently binding warhead.^4^, ^5^, ^6^, ^7^, ^8^ However, the application of SuFEx as anti-TB agents has not been reported. Working with the Sharpless group, we discovered that aryl fluorosulfate **1** with a diazo linker has good anti-TB activity with an IC_50_ = 1.1 µM from screening of a small library of 406 SuFEx compounds against Mtb in the attenuated lab H37Ra strain.

To address the photolabile issue of the diazo linker,^9^ we carried out a formal hit assessment, exploring multiple linkers including amide, 1,2,3-triazole, olefin, and ether. While most of these linkers were much less active or inactive than compound **1**, phenyl ether compound **2** exhibited comparable activity (H37Ra IC_50_ = 1.3 µM). We then performed further hit-to-lead optimization of compound **2** focusing on replacement of the metabolically labile amide tail and generated the *N1*-1,2,4-triazole-containing early lead **3a**, which shows only slightly decreased activity (H37Ra IC_50_ = 1.5 µM) (Fig. 1). Encouraged by these results, we conducted lead optimization of compound **3a** via detailed structure − activity relationship (SAR) studies, as described in this paper. The SAR campaign led to compound **21b** with good anti-TB activity (H37Rv MIC = 0.06 µM) and favorable pharmacokinetic (PK) properties for further preclinical development.

**Fig. 1:**
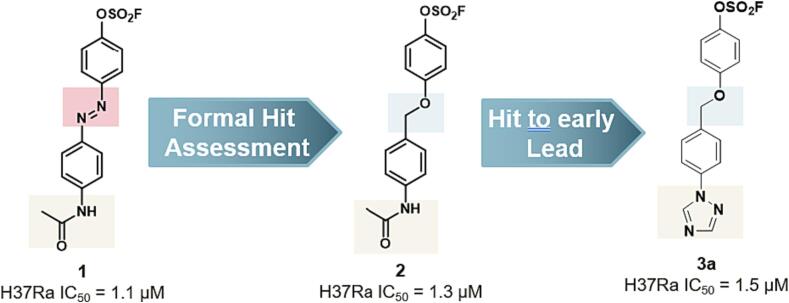
Hit to early lead **3a** from screening hit **1.**

We explored the SAR of the initial lead **3a** in three regions (Fig. 2). The general synthesis of aryl fluorosulfates started from a conversion of commercially available (4-bromophenyl)methanol derivatives (**4**) to 1-bromo-4-(chloromethyl)benzenes (**5**), followed by phenyl ether formation with methoxymethyl (MOM)-protected hydroquinones (**6**). After Buchwald amination with the corresponding triazoles (**8**), MOM was removed under an acidic condition. The resulting phenol (**10**) was converted to the desired aryl fluorosulfate **3a** and their analogs by sulfuryl fluoride (SO_2_F_2_) gas or a shelf-stable SuFEx reagent, 4-(acetylamino)phenyl]imidodisulfuryl difluoride (AISF),^10^ under a basic condition.

**Fig. 2:**
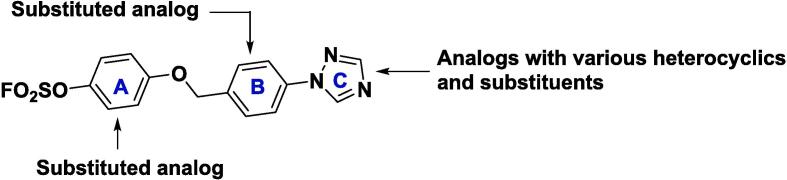
Chemical structure of aryl fluorosulfate **3a** and its derivatization for the SAR study.

A focused library of western phenyl (A) and middle phenyl (B) modified analogs was synthesized and evaluated for antitubercular activity by H37Rv MIC or CDC1551 IC_90_ (Table 1). The cytotoxicity against both HEK293T and HepG2 mammalian cells was also examined. Our early lead **3a** has moderate anti-TB activity with H37Rv MIC = 2.84 µM and CDC1551 IC_90_ = 4.71 µM. The substitution SAR on the western phenyl ring revealed that a methoxy (OMe) group at the ortho position to the SuFEx group is not tolerated: compounds **3b** was not active against Mtb. Replacement with a chloro group gave compound **3c** with similar activity to **3a** against CDC1551; however, **3c** exhibited cytotoxicity against HEK293T and HepG2 cell lines. Next, we turned our attention to middle phenyl modification. We began by testing mono-fluorinated and di-fluorinated analogs (**3d-3i**) against Mtb. In general, fluorination at the *meta* position to triazole increased potency: both **3d** and **3i** showed 4-fold increases in H37Rv MIC. While difluorinated analog **3i** showed moderate cytotoxicity against HepG2 (IC_50_ = 5.8 µM), it still had good selectivity index (SI = 9.6). Changing one fluoro to a more electron-deficient nitrile group gave compound **3j** with much decreased potency (MIC = 5.0 µM).

**Table 1:** *In vitro* anti-TB inhibitory activity and cytotoxicity of aryl fluorosulfate **3a-3j**

**Figure:**
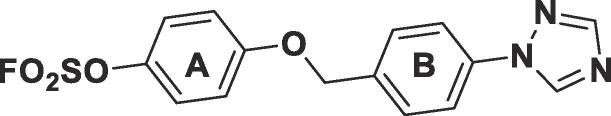

.

Compound	Phenyl A	Phenyl B	Anti-TB activity		Cytotoxicity	
			H37Rv MIC (µM)	CDC1551 IC_90_ (µM)	HEK293T CC_50_ (µM)	HepG2 CC_50_ (µM)
**3a**			2.84	4.71	11.6	19.3
**3b**			25	>30	>40	13.3
**3c**			ND	4.64	3.7	5.8
**3d**			0.6	1.3	22.7	12.5
**3e**			2.5	ND	16.8	17.6
**3f**			1.25	ND	18.8	22.4
**3 g**			10	ND	34.8	>40
**3 h**			1.25	ND	21.6	29.5
**3i**			0.6	ND	>40	5.8
**3j**			5	ND	>40	>40

ND = not determined.

In the context of difluoro-substituted phenyl, we further examined the activity of isomeric triazoles and other 5-membered heterocycles (Table 2). Analogs **3 k-3 m** were made with a general route shown in Scheme 1. Compounds **19a-19j** were synthesized using a modified procedure (Scheme 2) or using procedures described in supporting document. Using a similar procedure as in Scheme 1, phenol ether **14** was obtained, then phenyl bromide was transformed into phenyl boronic ester **15**. The following Suzuki coupling was used to install 5-membered heterocycles as exemplified by a trimethylsilylethoxymethyl (SEM)-protected triazole **16**. The subsequent SuFEx installation followed by SEM removal gave desired fluorosulfates **19a** and related analogs.

**Table 2:** *In vitro* anti-TB inhibitory activity and cytotoxicity of aryl fluorosulfates **3j, 3i-3o**, and **19a**-**19 m**

**Figure:**
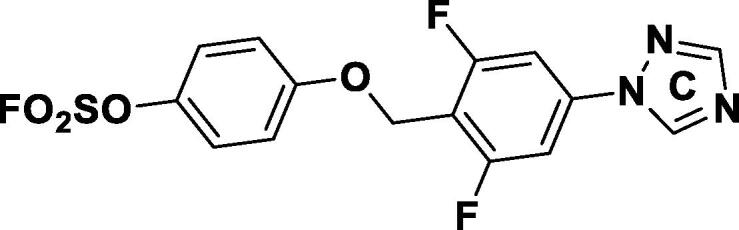

.

Compound	Heterocyclic ring C	Anti-TB activity	Cytotoxicity	
		H37Rv MIC (µM)	HEK293T CC_50_ µM)	HepG2 CC_50_ (µM)
**3i**		0.6	>40	5.8
**3 k**		1.23	>40	>40
**3 l**		0.16	27.7	17.0
**3 m**		0.22	>40	>40
**19a**		0.9	14.7	19.3
**19b**		1.25	ND	>40
**19c**		0.84	31.7	18.5
**19d**		0.3	>40	30.14
**19e**		1.25	26.0	13.7
**19f**		0.37	9.65	8.63
**19 g**		0.45	13.2	12.1
**19 h**		5	>40	>40
**19i**		0.62	>40	>40
**19j**		0.78	23.8	6.99

**Scheme 1:**
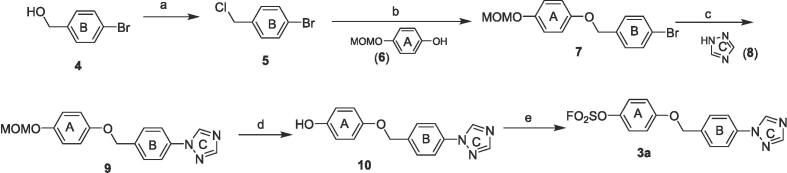
General synthetic route. Reagents and conditions: a) SOCl_2_, DMF/DCM, rt, 2 h; b) **6**, K_2_CO_3_, DMF, rt, 12 h; c) **8**, Pd_2_(dba)_3_, Me_4_-tBu-X-Phos, Cs_2_CO_3_, toluene, 100 °C, 5 h; d) Formic acid, DCM, rt, 2 h; e) SO_2_F_2_, TEA, DCM, rt, 30 min; or AISF, DBU, THF, 0 °C, 30 min.

**Scheme 2:**
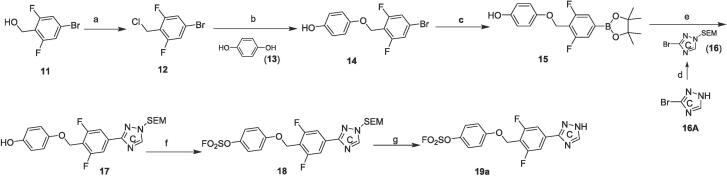
General synthetic route for compounds **19a-19j**: a) SOCl_2_, DMF/DCM, rt, 2 h; b) **13**, K_2_CO_3_, DMF, rt, 5 h; c) Pin_2_B_2_, Pd(dppf)Cl_2_, KOAc, dioxane, 100 °C, 2 h; d) SEMCl, TEA, DCM, rt, 12 h; e) **16**, Pd(dppf)Cl_2_, K_3_PO_4_, dioxane/H_2_O, 80 °C, 2 h; f) AISF, DBU, THF, rt, 30 min; g) TFA, DCM, rt, 1 h.

Among isomeric triazoles, *N*2-1,2,3 triazole analog **3 k** showed 2-fold decreased activity, and *N1*-1,2,3 triaozle **3 l** and *N1*-1,3,4-triazole **3 m** had 3-fold increases in MIC. *4H*-1,2,4-triazole analog **19a** had an MIC < 1.0 µM. We also synthesized other 5-membered heterocyclic analogs including pyrazole **19c**, imidazole **19d** and **19f**, and tetrazole **19 g** and **19 h**, and evaluated them for anti-TB activity. Some corresponding nitrogen-substituted methyl analogs, including **19b** and **19e** were also prepared. In general, these compounds had low activity compared to triazole analogs. In addition, oxadiazole **19i** and isoxazole **19j** were made and they exhibited MIC < 1 µM against Mtb. All analogs had good cytotoxicity profiles in HEK293T and HepG2 cell lines, except one imidazole analog **19f** that had a half maximal cellular cytotoxicity concentration 50 (CC_50_) < 10 µM.

We further examined the substitution effect with the *N1*-1,2,4- triazole system (Table 3). Most compounds were prepared with a general synthetic method as outlined in Scheme 1. 3-Methyl-substituted analog **20a** showed 2-fold increased potency, while ethyl- and isopropyl-substituted analogs **20b** and **20c** retained similar potency. The introduction of a more steric *t*-butyl (**20d**) resulted in 2-fold decreased activity with MIC = 1.2 µM.

**Table 3:** *In vitro* anti-TB inhibitory activity and cytotoxicity of aryl fluorosulfate **3i**, **20a-20 l**, and **21a**-**21f**

**Figure:**
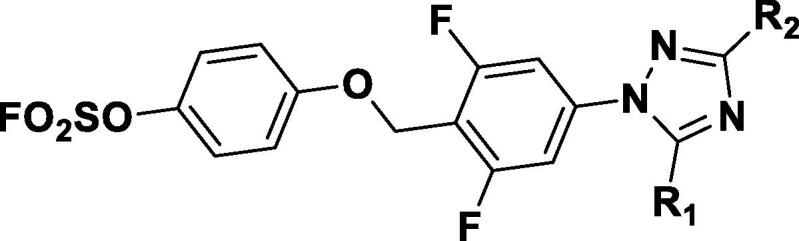

.

compound	R_1_	R_2_	Anti-TB activity	Cytotoxicity	
			H37Rv MIC (µM)	HEK293T CC_50_ (µM)	HepG2 CC_50_ (µM)
**3i**	**H**	**H**	0.6	>40	5.8
**20a**	**H**	**Me**	0.3	17.1	16.3
**20b**	**H**	**Et**	0.6	>40	5.8
**20c**	**H**	***i*Pr**	0.6	16.7	12.4
**20d**	**H**	***t*Bu**	1.2	13.7	6.9
**20e**	**Me**	**Me**	0.23	14.3	8.1
**20f**	**H**	**OMe**	0.6	>40	>40
**20 g**	**H**	**CN**	0.45	ND	10.3
**20 h**	**H**	**N(Me)_2_**	0.31	>40	>40
**20i**	**H**	**SO_2_Me**	0.40	17.2	9.3
**20j**	**H**	**COOH**	1.25	>40	>40
**20 k**	**H**	**CONH_2_**	0.17	>40	>40
**21a**	**H**	**NHCOMe**	0.15	>40	>40
**21b**	**H**	**NHSO_2_Me**	0.06	>40	>40
**20 l**	**H**	**SONH_2_**	0.47	ND	>40
**21c**	**H**	**N(Me)SO_2_Me**	0.31	ND	15.3
**21d**	**H**	**NHSO_2_Et**	0.08	ND	>40
**21e**	**H**	**NHSO_2_*i*Pr**	0.31	>40	>40
**21f**	**H**	**NHSO_2_*t*Bu**	2.5	>40	>40

We then synthesized 3,5-dimethyl analog disubstituted analog (**20e**), which had similar potency to 3-mono methyl analog **20a**. We also investigated an electronic effect by introducing electronic donating substituent methoxy (**20f**), and electronic withdrawing nitrile (**20 g**). Both showed similar activity to non-substituted analog **3i**. Dimethyl amine and methyl sulfone substituted triazole analogs **20 h** and **20i** had slightly improved activity. The addition of COOH showed low activity with MIC = 1.25 µM (**20j**).

Encouraged by these results, we explored further fine tuning with steric and electronic effects. Introduction of acetamide gave compound **20 k** a 4-fold increased anti-TB activity with MIC = 0.17 µM. The methyl amide analog **21a** had a similar potency. We observed further improved activity with the methyl sulfonamide-substituted analog **21b** which had an MIC = 0.06 µM and a 47-fold improvement in anti-TB activity compared to our initial lead **3a**. Compound **21b** showed no cytotoxicity against both HEK293T and HepG2 cell lines. We also conducted additional SAR around sulfonamide. The reversed sulfonamide analog **20 l** had much lower activity than **21b**. The *N*-methyl substituted analog **21c**, also had 4-fold decreased activity compared to **21b**, suggesting the free NH is needed for high potency. Exchanging methyl for ethyl in analog **21d** provided similar potency. However, isopropyl and *tert*butyl sulfonamide **21e** and **21f** had much lower anti-TB activity, with **21f** having a MIC = 2.5 µM. Compound **21 g**, the phenol version of compound **21b,** showed no activity in H37Rv MIC (Fig. 3), which confirmed that SuFEx group is essential for anti-TB activity.

**Fig. 3:**
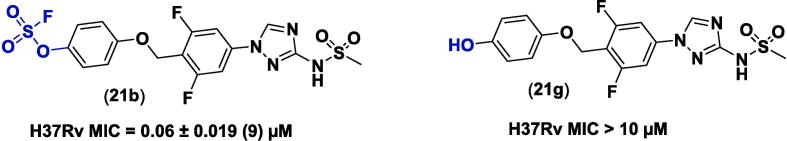
SuFEx is essential for anti-TB activity.

The synthesis of methyl sulfonamide-substituted analog **21b** was shown in Scheme 3. A optimized condition of step b, Chan-Lam Coupling,^11^ was used for the reaction of 3-nitro-1,2,4-triazole **24** with *tert*-butyldimethylsilyl (TBDMS) protected phenyl boronic ester **23**. The following hydrogenation (step c), TBDMS removal by *tetra-n*-butylammonium fluoride (TBAF) (step d) and SuFEx installation with ASIF (step e) gave amino triazole **28**. The reaction of **28** with methylsulfonyl chloride gave di-substituted analog **29**. Subsequent treatment with TBAF provided desired sulfonamide **21b**.

**Scheme 3:**
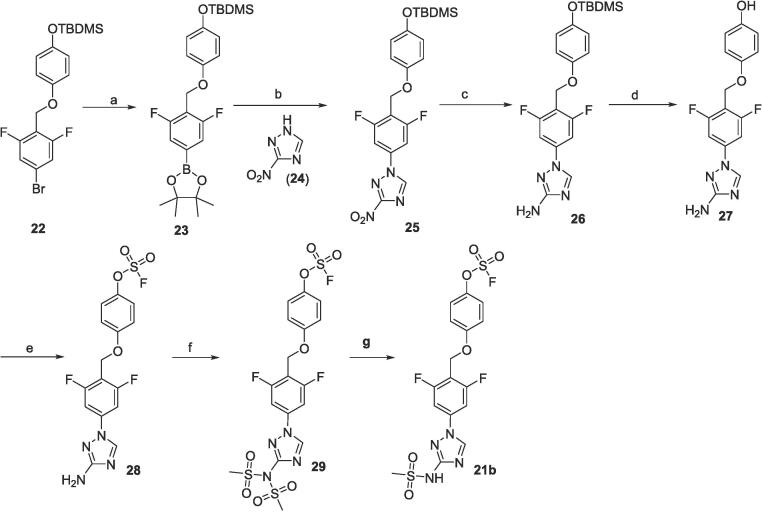
Synthetic route for compound **21b**: a) Pin_2_B_2_, Pd(dppf)Cl_2_, KOAc, dioxane, 100 °C, 2 h; b) **24**, Cu(OAc)_2_, pyridine, boric acid, CH_3_CN, 90 °C, 4 h; c) Pd/C, H_2_, THF; d) TABF, THF,0 °C - rt, 1 h; e) AISF, DBU, THF, 0 °C, 30 min; f) MeSO_2_Cl, TEA, DCM, 0 °C -rt, 1 h; g) TBAF, THF, 0 °C, 30 min.

With compound **21b** exhibiting good *in vitro* anti-TB activity and clean cytotoxicity profile, we next checked its PK properties. **21b** was administered to mice using both oral (PO) and intravenous (IV) routes at 20 mg/kg and 3 mg/kg in 75 % PEG/25 % D5W solution, respectively. In the PO arm, in addition to plasma sampling, we also examined compound residence in lung at 8 and 24 h post-dosing with consideration for TB treatment. Overall, compound **21b** showed good oral plasma exposure with excellent bioavailability (F = 99.4 %; Table 4). The 20 mg/kg PO dose achieved a near 24 h coverage of plasma and lung levels above the H37Rv MIC (MIC = 0.06 µM = 28 ng/mL) (Table 4). We further checked both mouse plasma and lung protein binding of **21b**: the unbound levels were 0.8 % and 1.6 %, respectively. As a result, the unbound levels of plasma and lung coverage over MIC were near 8 h at 20 mg/kg PO. These PK results supported advancing compound **21b** into a TB efficacy mouse model.

**Table 4:** Mouse PO/IV PK results of compound **21b.**

route	Dose (mg/kg)	Formulation	Cmax (ng/mL)	Tmax (hr)	T_1/2_ (hr)	Cl (mL/min/kg)	Vd (L/kg)	AUC_0-24_ (hr*ng/mL)	F (%)	C_plasma_ at 8 h (ng/mL)	C_lung_ at 8 h (ng/mL)	C_plasma_ at 24 h (ng/mL)	C_lung_ at 24 h (ng/mL)
PO	20	75 %PEG/25 % D5W solution	9,290	1.00	2.57	ND	ND	47,615	99.4	2,120	1,596	22.6	14.1
IV	3		3,314 (C0)	–	3.93	7.05	2.38	7,085	–	–	–	–	–

In conclusion, we developed a new class of arylfluorosulfate compounds with good *in vitro* activity against Mtb. Current lead **21b** has 0.06 µM H37Rv MIC, clean cytotoxicity profile and good mouse *in vivo* exposure. These aryl fluorosulfates added a novel chemotype for future evaluation in the TB drug development pipeline. We are performing detailed mechanism of action and efficacy studies, results which will be reported in due course.

## Declaration of competing interest

The authors declare that they have no known competing financial interests or personal relationships that could have appeared to influence the work reported in this paper.

## Data Availability

Data will be made available on request.

## References

[1] WHO, Global Tuberculosis Report 2022. https://www.who.int/teams/globaltuberculosis-programme/tb-reports/global-tuberculosis-report-2022 Retrieved 28 April 2023.

[2] Günthe G. (2014). Multidrug-resistant and extensively drug-resistant tuberculosis: a review of current concepts and future challenges. Clin Med J.

[3] Dong J., Sharpless K.B., Kwisnek L., Oakdale J.S., Fokin V.V. (2014). SuFEx-based synthesis of polysulfates. Angew Chem Int Ed.

[4] Chen W., Dong J., Plate L. (2016). Arylfluorosulfates inactivate intracellular lipid binding protein(s) through chemoselective SuFEx reaction with a binding site Tyr residue. J Am Chem Soc.

[5] Fadeyi O.O., Hoth L.R., Choi C. (2017). Covalent enzyme inhibition through fluorosulfate modification of a noncatalytic serine residue. ACS Chem Biol.

[6] Jones L.H. (2018). Emerging utility of fluorosulfate chemical probes. ACS Med Chem Lett.

[7] Zheng Q., Woehl J.L., Kitamura S. (2019). SuFEx-enabled, agnostic discovery of covalent inhibitors of human neutrophil elastase. Proc Natl Acad Sci.

[8] Bolding J.E., Martín-Gago P., Rajabi N. (2022). Aryl fluorosulfate based inhibitors that covalently target the SIRT5 lysine deacylase. Angew Chem Int Ed.

[9] S. Patai. *The chemistry of the hydrazo, azo and azoxy groups*. PATAI'S Chemistry of Functional Groups. John Wiley & Son, 1997. DOI:10.1002/0470023503.

[10] Zhou H., Mukherjee P., Liu R. (2018). Introduction of a crystalline, shelf-stable reagent for the synthesis of sulfur(VI) fluorides. Org Lett.

[11] Rao K.S., Wu T.S. (2012). Chan-Lam coupling reactions: synthesis of heterocycles. Tetrahedron.

