# Supplementary material for: Synthesis and structure–activity relationships of aryl fluorosulfate-based inhibitors as novel antitubercular agents
Source: Bioorg Med Chem Lett. 2024 Jan 15;98:129596. doi: 10.1016/j.bmcl.2023.129596 (PMC10808981; doi:10.1016/j.bmcl.2023.129596)

**Supplementary Material**

**Synthesis and Structure−Activity Relationships of Aryl Fluorosulfate-based Inhibitors as Novel Antitubercular Agents**

Baiyuan Yang^a^*, Paridhi Sukheja^a^, Bo Qin^a^, Grant A. L. Bare^b^, Alessandro Cascioferro^a^, Melissa S. Love^a^, H. Michael Petrassi^a^, K. Barry Sharpless^b^, Case W. McNamara^a^, Arnab K. Chatterjee^a^

^a^Calibr, a division of Scripps Research, La Jolla, CA 92037, USA; ^b^Department of Chemistry, Scripps Research, La Jolla 92037, CA, USA

*Corresponding author: [byang@scripps.edu](mailto:byang@scripps.edu)

- Experimental methods and materials, and representative synthesis procedures
- Biological assay protocols
- ^1^H and ^19^F NMR spectra for compounds, ^1^H, ^19^F NMR and ^13^C NMR for compounds **21b**

### **Experimental Methods and Materials**

All chemicals were commercially available except those whose synthesis is described or referenced.

**Representative Synthetic Procedures and Spectral Data**

*General Synthesis of aryl fluorosulfates* ***3a-3n*** *can be performed as exemplified for* ***3d***

4-((2-fluoro-4-(1H-1,2,4-triazol-1-yl)benzyl)oxy)phenyl sulfurofluoridate (**3d**)

**Step a:** To a solution of (4-bromo-2-fluoro-phenyl)methanol **4e** (2.00 g, 9.75 mmol, 1.00 *eq.*) in DCM (20 mL) was added SOCl_2_ (4.64 g, 39.0 mmol, 2.83 mL, 4.00 *eq.*) and DMF (71.3 mg, 976 umol, 75.1 uL, 0.10 *eq.*) at 0 °C. The mixture was stirred at 30 °C for 2 hours. The reaction mixture was diluted with water (200 mL) and extracted with DCM (200 mL × 3). The combined organic layers were washed with water (100 mL) and brine (100 mL × 2), dried over anhydrous sodium sulfate, filtered and concentrated under reduced pressure to give 4-bromo-1-(chloromethyl)-2-fluoro-benzene **5e** (1.96 g, 7.89 mmol, 81% yield, 90% purity) as a yellow oil, which is used directly in next step. ^1^H NMR (400 MHz, CDCl_3_) δ 7.26 - 7.15 (m, 3H), 4.50 (s, 2H).

**Step b**: To a solution of 4-(methoxymethoxy)phenol **6** (1.10 g, 7.15 mmol, 1.60 *eq.*) and 4-bromo-1-(chloromethyl)-2-fluoro-benzene **5e** (1.00 g, 4.47 mmol, 1.00 *eq.*) in DMF (10 mL) was added K_2_CO_3_ (1.85 g, 13.4 mmol, 3.00 *eq.*). The mixture was stirred at 25 °C for 12 hours. The reaction mixture was diluted with water (100 mL) and extracted with ethyl acetate (100 mL × 3). The combined organic layers were washed with water (100 mL) and brine (100 mL × 2), dried over anhydrous sodium sulfate, filtered and concentrated under reduced pressure to give a residue. The residue was purified by column chromatography (SiO2, Petroleum ether/Ethyl acetate=15/1 to 2/1) to give 4-bromo-2-fluoro-1-[[4-(methoxymethoxy) phenoxy]methyl]benzene **7e** (600 mg, 1.51 mmol, 34% yield, 86% purity) as a yellow oil.

**Step c**: A mixture of 4-bromo-2-fluoro-1-[[4-(methoxymethoxy)phenoxy]methyl]benzene **7e** (200 mg, 504 umol, 86% purity, 1.00 *eq.*), ditert-butyl-[2,3,4,5-tetramethyl-6-(2,4,6-triisopropyl phenyl)phenyl]phosphane (48.5 mg, 101 umol, 0.20 *eq.*), 1*H*-1,2,4-triazole **8** (69.6 mg, 1.01 mmol, 2.00 *eq.*), Pd_2_(dba)_3_ (46.2mg, 50.4 umol, 0.10 *eq.*) and Cs_2_CO_3_ (329 mg, 1.01 mmol, 2.00 *eq.*) in toluene (2 mL) was degassed and purged with N_2_ for 3 times, and then the mixture was stirred at 100 °C for 5 hours under N_2_ atmosphere. The reaction mixture was diluted with water (10 mL) and extracted with ethyl acetate (20 mL × 3). The combined organic layers were washed with water (20 mL) and brine (10 mL × 2), dried over anhydrous sodium sulfate, filtered and concentrated under reduced pressure to give a residue. The residue was purified by prep-HPLC (column: Waters X bridge 150 × 25mm × 5 um; mobile phase: [water (10 mM NH_4_HCO_3_) - CAN]; B%: 40% - 70%, 8min). The desired fraction was collected and lyophilized to give 1-[3-fluoro-4-[[4-(methoxy methoxy)phenoxy]methyl]phenyl]-1,2,4- triazole **9e** (90.0 mg, 265 umol, 53% yield, 97% purity) as a yellow oil. LCMS [ESI, M+1]: 330.1; ^1^H NMR (400 MHz, CDCl_3_) δ 8.62 (s, 1H), 8.13 (s, 1H), 7.67 (t, *J* = 8.0 Hz, 1H), 7.55 - 7.48 (m, 2H), 7.05 - 6.98 (m, 2H), 6.95 - 6.88 (m, 2H), 5.14 (s, 2H), 5.13 (s, 2H), 3.49 (s, 3H).

**Step d**: Formic acid (1.22 g, 26.5 mmol, 1.00 mL, 175 *eq.*) was added to a solution of 1-[3-fluoro-4-[[4-(methoxymethoxy)phenoxy]methyl]phenyl]-1,2,4-triazo le **9e** (50.0 mg, 151 umol, 1.00 *eq.*). The mixture was stirred at 25 °C for 2 hours. The reaction mixture was diluted with MeCN (5 mL) and concentrated under reduced pressure to give 4-[[2-fluoro-4-(1,2,4-triazol-1-yl)phenyl] methoxy]phenol **10e** (20.0 mg, 56.1 umol, 37% yield, 80% purity) as a yellow oil. LCMS [ESI, M+1]: 286.0

**Step e**: To a solution of 4-[[2-fluoro-4-(1,2,4-triazol-1-yl) phenyl]methoxy]phenol **10e** (20.0 mg, 56.1 umol, 80% purity, 1.00 *eq.*) in DCM (1 mL) was added TEA (22.7 mg, 224 umol, 31.2 uL, 4.00 *eq.*), sulfuryl fluoride (8.59 mg, 84.1 umol, 1.50 *eq.*) was bubbled into the mixture. The mixture was stirred at 25 °C for 1 hour. The reaction mixture was concentrated under reduced pressure to give a residue. The residue was purified by prep-HPLC (column: Phenomenex luna C18 150 × 40 mm × 15 um; mobile phase: [water (0.2%FA) - ACN]; B%: 48% - 78%, 10 min). The desired fraction was collected and lyophilized to give 1-[3-fluoro-4-[(4-fluorosulfonyloxyphenoxy)methyl]phenyl]-1,2,4-triazole **3d** (2.28 mg, 6.14 umol, 11% yield, 99% purity) as a gray solid. LCMS [ESI, M+1]: 367.9; ^1^H NMR (400 MHz, CDCl_3_) δ 8.60 (s, 1H), 8.13 (s, 1H), 7.65 (t, *J* = 8.0 Hz, 1H), 7.59 - 7.48 (m, 2H), 7.30 (d, *J* = 9.2 Hz, 2H), 7.06 (d, *J* = 9.2 Hz, 2H), 5.19 (s, 2H); ^19^F NMR (377 MHz, CDCl_3_) δ 36.62, -114.60.

4-((4-(1H-1,2,4-triazol-1-yl)benzyl)oxy)phenyl sulfurofluoridate (**3a**)

Obtained as a white solid. LCMS [ESI, M+1]: 350.0; ^1^H NMR (400 MHz, DMSO-*d_6_*) δ 9.31(s, 1H), 8.25 (s, 1H), 7.90 (m, 2H), 7.65 (m, 2H); 7.54 (m, 2H), 7.20 (m, 2H), 5.23 (s, 2H).

4-((4-(1H-1,2,4-triazol-1-yl)benzyl)oxy)-2-methoxyphenyl sulfurofluoridate (**3b**)

Obtained as a white solid. LCMS [ESI, M+1]: 380.1; ^1^H NMR (400 MHz, DMSO-*d_6_*) 9.33 (s, 1H), 8.26 (s, 1H), 7.91 (d, *J* = 8.1 Hz, 2H), 7.67 (d, *J* = 8.1 Hz, 2H), 7.51 (d, *J* = 9.1 Hz, 1H), 6.99 (s, 1H), 6.72 (d, *J* = 9.2 Hz, 1H), 5.23 (s, 2H), 3.90 (s, 3H).

4-((4-(1H-1,2,4-triazol-1-yl)benzyl)oxy)-2-chlorophenyl sulfurofluoridate (**3c**)

Obtained as a white solid. LCMS [ESI, M+1]: 384.1; ^1^H NMR (400 MHz, DMSO-*d_6_*) δ 9.34 (s, 1H), 8.26 (s, 1H), 7.97 – 7.87 (m, 2H), 7.77 (d, *J* = 9.0 Hz, 1H), 7.66 (d, *J* = 8.5 Hz, 2H), 7.51 (m, 1H), 7.21 (dd, *J* = 9.2, 2.9 Hz, 1H), 5.26 (s, 2H).

4-((3-fluoro-4-(1H-1,2,4-triazol-1-yl)benzyl)oxy)phenyl sulfurofluoridate (**3e**)

Obtained as white solid. LCMS [ESI, M+1]: 368.1; 1H NMR (400 MHz, DMSO-*d_6_*) δ 9.03 (d, J = 2.4 Hz, 1H), 8.30 (s, 1H), 7.83 (t, *J* = 8.0 Hz, 1H), 7.64 (d, *J* = 11.6 Hz, 1H), 7.56 (d, *J* = 9.2 Hz, 2H), 7.50 (d, *J* = 8.0 Hz, 1H), 7.22 (d, *J* = 9.2 Hz, 2H), 5.27 (s, 2H); 19F NMR (400 MHz, DMSO-*d_6_*) δ 37.49, -123.51.

4-((2,3-difluoro-4-(1H-1,2,4-triazol-1-yl)benzyl)oxy)phenyl sulfurofluoridate (**3f**)

Obtained as a white solid. LCMS [ESI, M+1]: 386.0; ^1^H NMR (400 MHz, DMSO-*d_6_*) δ 9.10 (d, *J* = 2.4 Hz, 1H), 8.35 (s, 1H), 7.74 - 7.66 (m, 1H), 7.63 - 7.59 (m, 1H), 7.59 - 7.53 (m, 2H), 7.30 - 7.20 (m, 2H), 5.33 (s, 2H); ^19^F NMR (400 MHz, DMSO-*d_6_*) δ 37.51, -140.01, -147.39.

4-((3,5-difluoro-4-(1H-1,2,4-triazol-1-yl)benzyl)oxy)phenyl sulfurofluoridate (**3g**)

Obtained as a brown solid. LCMS [ESI, M+1]: 386.0; ^1^H NMR (400 MHz, CDCl_3_) δ 8.94 - 8.48 (m, 1H), 8.47 - 8.10 (m, 1H), 7.32 (d, *J* = 8.8 Hz, 2H), 7.24 (br d, *J* = 8.8 Hz, 2H), 7.03 (d, *J* = 9.2 Hz, 2H), 5.15 (s, 2H); ^19^F NMR (377 MHz, CDCl_3_) δ 36.73, -117.83.

4-((2,5-difluoro-4-(1H-1,2,4-triazol-1-yl)benzyl)oxy)phenyl sulfurofluoridate (**3h**)

Obtained as a light yellow solid. LCMS [ESI, M+1]: 386.0; ^1^H NMR (400 MHz, DMSO-*d_6_*) δ 9.08 (d, *J* = 2.0 Hz, 1H), 8.34 (s, 1H), 7.83 (td, *J* = 6.4, 10.4 Hz, 2H), 7.57 (br d, *J* = 9.0 Hz, 2H), 7.25 (d, *J* = 9.2 Hz, 2H), 5.26 (s, 2H); ^19^F NMR (400 MHz, *DMSO-d_6_*) δ 37.52, -120.17, -127.63.

4-((2,6-difluoro-4-(1H-1,2,4-triazol-1-yl)benzyl)oxy)phenyl sulfurofluoridate (**3i**)

Obtained a white solid. LCMS [ESI, M+1]: 386.0; ^1^H NMR (400 MHz, DMSO-*d_6_*) δ 9.43 (s, 1H), 8.33 (s, 1H), 7.82 (br d, *J* = 8.4 Hz, 2H), 7.58 (br d, *J* = 8.8 Hz, 2H), 7.24 (br d, *J* = 9.2 Hz, 2H), 5.21 (s, 2H); ^19^F NMR (400 MHz, DMSO-*d_6_*) δ 37.53, -111.91.

4-((2-cyano-6-fluoro-4-(1H-1,2,4-triazol-1-yl)benzyl)oxy)phenyl sulfurofluoridate (**3j**)

Obtained as a white solid. LCMS [ESI, M+1]: 392.9; 1H NMR (400 MHz, DMSO-*d_6_*) δ 9.47 (s, 1H), 8.41 (d, *J* = 1.2 Hz, 1H), 8.36 (s, 1H), 8.27 (dd, *J* = 1.8, 10.6 Hz, 1H), 7.60 (d, *J* = 9.0 Hz, 2H), 7.32 - 7.22 (m, 2H), 5.32 (s, 2H);^19^F NMR (377 MHz, DMSO-*d_6_*) δ 37.61, -111.22.

4-((2,6-difluoro-4-(2H-1,2,3-triazol-2-yl)benzyl)oxy)phenyl sulfurofluoridate (**3k**)

Obtained as a white solid. LCMS [ESI, M+1]: 385.9; ^1^H NMR (400 MHz, DMSO-*d_6_*) δ 8.25 (s, 2H), 7.83 (d, *J* = 8.4 Hz, 2H), 7.58 (d, *J* = 9.2 Hz, 2H), 7.25 (d, *J* = 9.2 Hz, 2H), 5.23 (s, 2H); ^19^F NMR (400 MHz, DMSO-*d_6_*) δ 37.55, -111.57.

4-((2,6-difluoro-4-(1H-1,2,3-triazol-1-yl)benzyl)oxy)phenyl sulfurofluoridate (**3l**)

Obtained as a white solid. LCMS [ESI, M+1]: 386.1; ^1^H NMR (400 MHz, CD_3_OD) δ 8.68 (d, *J* = 1.3 Hz, 1H), 7.95 (d, *J* = 1.3 Hz, 1H), 7.87 – 7.68 (m, 2H), 7.50 – 7.36 (m, 2H), 7.30 – 7.15 (m, 2H), 5.26 (s, 2H). ^19^F NMR (400 MHz, CD_3_OD) δ 31.7, -115.8.

4-((2,6-difluoro-4-(1H-1,2,3-triazol-1-yl)benzyl)oxy)phenyl sulfurofluoridate (**3m**)

Obtained as a yellow solid. LCMS [ESI, M+1]: 386.0; ^1^H NMR (400 MHz, CDCl_3_) δ 8.54 (s, 2H), 7.31 (d, *J* = 9.2 Hz, 2H), 7.16 - 7.02 (m, 4H), 5.17 (s, 2H); ^19^F NMR (400 MHz, CDCl_3_) δ 36.66, -109.01.

*General Synthesis of aryl fluorosulfates* ***19a-19J*** *can be performed as exemplified for* ***19a***

4-((2,6-difluoro-4-(1*H*-1,2,4-triazol-3-yl)benzyl)oxy)phenylsulfurofluoridate (**19a**)

**Step a:** To a solution of (4-bromo-2,6-difluoro-phenyl)methanol **11** (15.0 g, 67.2 mmol, 1.00 *eq.*) in DCM (100 mL) was added SOCl_2_ (32.0 g, 269 mmol, 19.5 mL, 4.00 *eq.*) and DMF (491 mg, 6.73 mmol, 517 uL, 0.10 *eq*.). The mixture was stirred at 25 °C for 2 hrs. The pH of the mixture was adjusted with NaHCO_3_ aqueous solution to 7. Then the solution was extracted with EA (300 mL × 3), and the combined organic layer was washed with brine (500 mL), dried over Na_2_SO_4_. The reaction mixture was concentrated under reduced pressure to give 5-bromo-2-(chloromethyl)-1,3-difluoro-benzene **12** (13.0 g, crude) as a yellow oil.

**Step b:** To a solution of 5-bromo-2-(chloromethyl)-1,3-difluoro-benzene 12 (11.0 g, 45.5 mmol, 1.00 eq.) and benzene-1,4-diol **13** (7.52 g, 68.3 mmol, 10.1 mL, 1.50 eq.) in DMF (100 mL) was added K_2_CO_3_ (18.8 g, 136 mmol, 3.00 eq.). The mixture was stirred at 20 °C for 5 hrs. The reaction mixture was diluted with water (300 mL) and extracted with ethyl acetate (200 mL × 3). The combined organic layers were washed with brine (200 mL × 2), dried over anhydrous sodium sulfate, filtered and concentrated under reduced pressure to give a residue. The residue was purified by column chromatography (SiO_2_, Petroleum ether/Ethyl acetate = 30/1 to 3/1) to give compound 4-[(4-bromo-2,6-difluoro-phenyl)methoxy]phenol **14** (8.00 g, 22.8 mmol, 50% yield, 90% purity) as a white solid. ^1^H NMR (400 MHz, DMSO-*d_6_*) δ 9.00 (s, 1H), 7.54 (d, *J* = 7.2 Hz, 2H), 6.82 (d, *J* = 8.8 Hz, 2H), 6.67 (d, *J* = 8.8 Hz, 2H), 4.96 (s, 2H).

**Step c:** A mixture of 4-[(4-bromo-2,6-difluoro-phenyl)methoxy]phenol **14** (7.50 g, 23.8 mmol, 1.00 eq.), 4,4,5,5-tetramethyl-2-(4,4,5,5-tetramethyl-1,3,2-dioxaborolan-2-yl)-1,3,2-dioxaborolane (9.07 g, 35.7 mmol, 1.50 eq.), Pd(dppf)Cl_2_ (1.74 g, 2.38 mmol, 0.10 eq.) and AcOK (5.84 g, 59.5 mmol, 2.50 eq.) in dioxane (80.0 mL) was degassed and purged with N_2_ for 3 times, and then the mixture was stirred at 100 °C for 2 hrs under N_2_ atmosphere. The reaction mixture was filtered and concentrated under reduced pressure to give a residue. The residue was purified by column chromatography (SiO_2_, Petroleum ether/Ethyl acetate = 10/1 to 2/1) to give compound 4-[[2,6- difluoro- 4- (4,4,5,5- tetramethyl -1,3,2-dioxaborolan-2-yl) phenyl]methoxy]phenol **15** (9.0 g, 22.4 mmol, 93% yield, 90% purity) as a yellow solid. ^1^H NMR (400 MHz, DMSO-*d_6_*) δ 9.00 (s, 1H), 7.28 (d, *J* = 7.2 Hz, 2H), 6.82 (d, *J* = 9.2 Hz, 2H), 6.67 (d, *J* = 8.8 Hz, 2H), 5.01 (s, 2H), 1.30 (s, 12H).

**Step d:** To a solution of 3-bromo-1*H*-1,2,4-triazole **16a** (5.00 g, 33.8 mmol, 1.00 eq) and 2-(chloromethoxy)ethyl-trimethyl-silane (16.9 g, 101 mmol, 17.9 mL, 3.00 eq.) in DCM (50.0 mL) was added TEA (10.3 g, 101 mmol, 14.1 mL, 3.00 eq.). The mixture was stirred at 25 °C for 12 hrs. The reaction mixture was concentrated under reduced pressure to give a residue. The residue was purified by column chromatography (SiO_2_, Petroleum ether/Ethyl acetate = 30/1 to 3/1) to give compound 3-bromo-1-((2-(trimethylsilyl)ethoxy)methyl)-1*H*-1,2,4-triazole **16** (4.60 g, 14.8 mmol, 44% yield, 90% purity) as a yellow oil. LCMS [ESI, M+1]: 279.2.

**Step e**: A mixture of 4-[[2,6-difluoro-4-(4,4,5,5-tetramethyl-1,3,2-dioxaborolan-2-yl)phenyl]methoxy]phenol **15** (3.51 g, 9.70 mmol, 1.00 eq.**)**, 2-[(3-bromo-1,2,4-triazol-1-yl)methoxy]ethyl-trimethyl-silane **16** (5.4 g, 19.4 mmol, 2 eq.), K_3_PO_4_ (6.18 g, 29.1 mmol, 3.00 eq.) and ditert-butyl(cyclopentyl)phosphane;dichloropalladium;iron (632 mg, 970 umol, 0.10 eq.) in dioxane (70.0 mL) and H_2_O (14.0 mL) was degassed and purged with N_2_ for 3 times, and then the mixture was stirred at 80 °C for 2 hrs under N_2_ atmosphere (15 psi). The reaction mixture was diluted with water (50 mL) and extracted with ethyl acetate (60 mL × 3). The combined organic layers were washed with brine (60 mL × 2), dried over anhydrous sodium sulfate, filtered and concentrated under reduced pressure to give a residue. The residue was purified by column chromatography (SiO_2_, Petroleum ether/Ethyl acetate = 20/1 to 1/1) to give compound 4-[[2,6-difluoro-4-[1-(2-trimethylsilylethoxymethyl)-1,2,4-triazol-3-yl]phenyl]methoxy]phenol **17** (3.00 g, 6.64 mmol, 68% yield, 96%

purity) as a yellow oil. LCMS [ESI, M+1]: 434.3.

**Step f**: To a solution of 4-[[2,6-difluoro-4-[1-(2-trimethylsilylethoxymethyl)-1,2,4-triazol-3-yl]phenyl]methoxy]phenol **17** ( 2.80 g, 6.46 mmol, 1.00 eq.) in DCM (30.0 mL) was added TEA (2.61 g, 25.8 mmol, 3.60 mL, 4.00 eq.) under sulfuryl fluoride (659 mg, 6.46 mmol, 1.00 eq.) atmosphere (15 psi). The mixture was stirred at 20 °C for 1 hr. The reaction mixture was diluted with water (30 mL) and extracted with ethyl acetate (50 mL × 3). The combined organic layers were washed with brine (50 mL × 2), dried over anhydrous sodium sulfate, filtered and concentrated under reduced pressure to give a residue. The residue was purified by column chromatography (SiO_2_, Petroleum ether/Ethyl acetate = 10/1 to 1/1) to give 3-[3,5-difluoro-4-[(4-fluorosulfonyloxyphenoxy)methyl]phenyl]-1-(2-trimethylsilylethoxymethyl)-1,2,4-triazole **18** (2.50 g, 4.61 mmol, 71% yield, 95% purity) as a yellow oil. LCMS [ESI, M+1]: 516.1.

**Step g**: To a solution of 3-[3,5-difluoro-4-[(4-fluorosulfonyloxyphenoxy)methyl]phenyl]-1-(2-trimethylsilylethoxymethyl)-1,2,4-triazole **18** (2.30 g,4.46 mmol, 1.00 *eq.*) in DCM (30.0 mL) was added TFA (24.4 g, 214 mmol,15.8 mL, 48.0 *eq.*). The mixture was stirred at 20 °C for 2 hrs. The reaction mixture was concentrated under reduced pressure to give a residue. The residue was purified by column chromatography (SiO_2_, Petroleum ether/Ethyl acetate = 10/1 to 1/1) to give 3-[3,5-difluoro-4-[(4-fluorosulfonyloxyphenoxy)methyl]phenyl]-1*H*-1,2,4-triazole **19a** (950 mg, 2.44 mmol, 54% yield, 99% purity) as a white solid. LCMS [ESI, M+1]: 385.9; ^1^H NMR (400 MHz, DMSO-*d_6_*) δ 14.43 (s, 1H), 8.65 (s, 1H), 7.72 (d, *J* = 8.0 Hz, 2H), 7.57 (d, *J* = 8.0 Hz, 2H), 7.25 - 7.22 (m, 2H), 5.21 (s, 2H); ^19^F NMR (377 MHz, DMSO-*d_6_*) δ 37.54, -113.84.

4-((2,6-difluoro-4-(1-methyl-1H-1,2,4-triazol-3-yl)benzyl)oxy)phenyl sulfurofluoridate (**19b**)

Obtained as a white solid. LCMS [ESI, M+1]: 400.1; ^1^H NMR (400 MHz, DMSO-*d_6_*) δ 8.63 (s, 1H), 7.69 (d, *J* = 8.1 Hz, 2H), 7.58 (d, *J* = 9.2 Hz, 2H), 7.25 (d, *J* = 9.2 Hz, 2H), 5.21 (s, 2H), 3.96 (s, 3H).

4-((2,6-difluoro-4-(1H-pyrazol-5-yl)benzyl)oxy)phenyl sulfurofluoridate (**19c**)

Obtained as a white solid. LCMS [ESI, M+1]: 385.0; ^1^H NMR (400 MHz, DMSO-*d_6_*) δ 7.82 (d, *J* = 2.4 Hz, 1H), 7.62 (d, *J* = 8.8 Hz, 2H), 7.55 (d, *J* = 9.2 Hz, 2H), 7.31 - 7.17 (m, 2H), 6.91 (d, *J* = 2.4 Hz, 1H), 6.48 (br s, 4H), 5.17 (s, 2H); ^19^F NMR (377 MHz, DMSO-*d_6_*) δ 37.51, -114.85.

4-((2,6-difluoro-4-(1H-imidazol-2-yl)benzyl)oxy)phenyl sulfurofluoridate (**19d**)

Obtained as a white solid. LCMS [ESI, M+1]: 384.9; ^1^H NMR (400 MHz, DMSO-*d_6_*) δ 7.76 (d, *J* = 8.4 Hz, 2H), 7.58 (d, *J* = 8.8 Hz, 2H), 7.48 (s, 2H), 7.23 (d, *J* = 9.2 Hz, 2H), 5.20 (s, 2H); ^19^F NMR (377 MHz, DMSO-*d_6_*) δ 37.53, -113.42.

4-((2,6-difluoro-4-(1-methyl-1H-imidazol-2-yl)benzyl)oxy)phenyl sulfurofluoridate (**19e**)

Obtained as a white solid. LCMS [ESI, M+1]: 399.0; ^1^H NMR (400 MHz, DMSO-*d_6_*) δ 7.58 – 7.51 (m, 4H), 7.34 (d, *J* = 12 Hz, 1 H), 7.26 – 7.23 (m, 2 H), 7.03 (d, *J* = 12 Hz, 1 H), 5.21 (s, 2H), 3.89 (s, 3H). ^19^F NMR (400MHz, DMSO-*d_6_*) δ 37.5, -114.2.

4-((2,6-difluoro-4-(1H-imidazol-1-yl)benzyl)oxy)phenyl sulfurofluoridate (**19f**)

Obtained as a brown solid. LCMS [ESI, M+1]: 385.1; ^1^H NMR (400 MHz, DMSO-*d_6_*) δ 8.46 (s, 1H), 7.92 (s, 1H), 7.73 (d, *J* = 8.8 Hz, 2H), 7.57 (d, *J* = 9.2 Hz, 2H), 7.23 (d, *J* = 9.2 Hz, 2H), 7.15 (s, 1H), 5.18 (s, 2H); ^19^F NMR (377 MHz, DMSO-*d_6_*) δ 37.53, -112.58.

4-((2,6-difluoro-4-(1H-tetrazol-1-yl)benzyl)oxy)phenyl sulfurofluoridate (**19g**)

Obtained as a black oil. LCMS [ESI, M+1]: 387.1; ^1^H NMR (400 MHz, DMSO-*d_6_*) δ 10.17 (s, 1H), 7.94 (d, *J* = 8.0 Hz, 2H), 7.58 (d, *J* = 9.2 Hz, 2H), 7.24 (d, *J* = 9.2 Hz, 2H), 5.25 (s, 2H); ^19^F NMR (400 MHz, DMSO-*d_6_*) δ - 37.56, 111.20.

4-((2,6-difluoro-4-(1H-tetrazol-5-yl)benzyl)oxy)phenyl sulfurofluoridate (**19h**)

Obtained as a yellow solid. LCMS [M+1]: 386.8; ^1^H NMR (400 MHz, DMSO-*d_6_*) δ 7.79 (br d, *J* = 8.0 Hz, 2H), 7.57 (br d, *J* = 8.8 Hz, 2H), 7.24 (br d, *J* = 9.2 Hz, 2H), 5.23 (s, 2H); ^19^F NMR (377 MHz, DMSO-*d_6_*) δ 37.54, -113.07.

4-((2,6-difluoro-4-(1,2,4-oxadiazol-3-yl)benzyl)oxy)phenyl sulfurofluoridate (**19i**)

Obtained as a white solid. LCMS [M+1]: 387.0; ^1^H NMR (400 MHz, DMSO-*d_6_*) δ 9.83 (s, 1H), 7.81 (d, *J* = 7.6 Hz, 2H), 7.58 (d, *J* = 8.8 Hz, 2H), 7.25 (m, 2H), 5.27 (s, 2H); ^19^F NMR (377 MHz, DMSO-*d_6_*) δ = 37.56, -112.27, -112.29.

4-((2,6-difluoro-4-(isoxazol-4-yl)benzyl)oxy)phenyl sulfurofluoridate **(19j)**

Obtained as a white solid. LCMS [M+1]: 386.0; 1H NMR (400 MHz, CD_3_OD) δ 9.23 (s, 1H), 8.93 (s, 1H), 7.48 – 7.32 (m, 4H), 7.16 (d, *J* = 9.2 Hz, 2H), 5.19 (s, 2H); ^19^F NMR (377 MHz, CD_3_OD) δ 38.30, -112.10.

4-((2,6-difluoro-4-(3-methyl-1H-1,2,4-triazol-1-yl)benzyl)oxy)phenyl sulfurofluoridate **(20a)**

Obtained as a yellow solid; LCMS [ESI, M+1]: 400.0; ^1^H NMR (400 MHz, CDCl_3_) δ 8.53 (s, 1H), 7.40 - 7.28 (m, 4H), 7.05 (d, *J* = 9.2 Hz, 2H), 5.14 (s, 2H), 2.50 (s, 3H); ^19^F NMR (400 MHz, CDCl_3_) δ 36.59, -110.70.

4-((4-(3-ethyl-1H-1,2,4-triazol-1-yl)-2,6-difluorobenzyl)oxy)phenyl sulfurofluoridate (**20b**)

Obtained as a white solid. LCMS [M+1]: 414.0; ^1^H NMR (400 MHz, DMSO-*d_6_*) δ 9.31 (s, 1H), 7.77 (d, *J* = 8.5 Hz, 2H), 7.59 (d, *J* = 9.1 Hz, 2H), 7.25 (d, *J* = 9.2 Hz, 2H), 5.20 (s, 2H), 2.75 (d, *J* = 7.6 Hz, 2H), 1.28 (t, J = 7.6 Hz, 3H).

4-((2,6-difluoro-4-(3-isopropyl-1H-1,2,4-triazol-1-yl)benzyl)oxy)phenyl sulfurofluoridate (**20c**)

Obtained as a white solid. LCMS [M+1]: 428.0; ^1^H NMR (400 MHz, DMSO-*d_6_*) δ 9.30 (s, 1H), 7.76 (d, *J* = 8.5 Hz, 2H), 7.66 – 7.49 (m, 2H), 7.32 – 7.16 (m, 2H), 5.20 (s, 2H), 3.07 (m, 1H), 1.31 (d, *J* = 6.9 Hz, 6H).

4-((4-(3-(tert-butyl)-1H-1,2,4-triazol-1-yl)-2,6-difluorobenzyl)oxy)phenyl sulfurofluoridate (**20d**)

Obtained as a white solid. LCMS [M+1]: 442.0; ^1^H NMR (400 MHz, DMSO-*d_6_*) δ 9.29 (s, 1H), 7.76 (d, *J* = 8.4 Hz, 2H), 7.59 (d, *J* = 9.1 Hz, 2H), 7.25 (d, *J* = 9.2 Hz, 2H), 5.20 (s, 2H), 1.36 (s, 9H).

4-((4-(3,5-dimethyl-1H-1,2,4-triazol-1-yl)-2,6-difluorobenzyl)oxy)phenyl sulfurofluoridate (**20e**)

Obtained as a yellow solid; LCMS [M+1]: 413.9; ^1^H NMR (400 MHz, CDCl_3_) δ 7.29 (d, *J* = 8.8 Hz, 2H), 7.16 (d, *J* = 7.6 Hz, 2H), 7.06 (d, *J* = 9.2 Hz, 2H), 5.16 (s, 2H), 2.58 (s, 3H), 2.40 (s, 3H); ^19^F NMR (377 MHz, CDCl_3_) δ 36.59, -111.34.

4-((2,6-difluoro-4-(3-methoxy-1H-1,2,4-triazol-1-yl)benzyl)oxy)phenyl sulfurofluoridate (**20f**)

Obtained as a yellow solid. LCMS [ESI, M+1]: 416.0; ^1^H NMR (400 MHz, DMSO-*d_6_*) δ 9.16 (s, 1H), 7.71 (d, *J* = 8.8 Hz, 2H), 7.57 (d, *J* = 8.8 Hz, 2H), 7.23 (d, *J* = 9.2 Hz, 2H), 5.19 (s, 2H), 3.98 (s, 3H); ^19^F NMR (377 MHz, DMSO-*d_6_*) δ 37.54, -112.14.

4-((4-(3-cyano-1H-1,2,4-triazol-1-yl)-2,6-difluorobenzyl)oxy)phenyl sulfurofluoridate (**20g**)

Obtained as a yellow solid. LCMS [ESI, M-1]: 409.2; ^1^H NMR (400 MHz, DMSO-*d_6_*) δ 9.72 (s, 1H), 7.91 (m, 2H), 7.57 (d, *J* = 8.8 Hz, 2H), 7.23 (m, 2H), 5.24 (s, 2H); ^19^F NMR (377 MHz, DMSO-*d_6_*) δ 37.57, -111.19, -111.22.

4-((2,6-difluoro-4-(3-isopropyl-1H-1,2,4-triazol-1-yl)benzyl)oxy)phenyl sulfurofluoridate (**20h**)

Obtained as a white solid. LCMS [ESI, M+1]: 428.9; ^1^H NMR (400 MHz, DMSO-*d_6_*) δ 9.06 (s, 1H), 7.69 - 7.61 (m, 2H), 7.57 (d, *J* = 8.8 Hz, 2H), 7.28 - 7.19 (m, 2H), 5.16 (s, 2H), 2.98 (s, 6H); ^19^F NMR (377 MHz, DMSO-*d_6_*) δ 37.52, -112.65.

4-((2,6-difluoro-4-(3-(methylsulfonyl)-1H-1,2,4-triazol-1-yl)benzyl)oxy)phenyl sulfurofluoridate (**20i**)

Obtained as a yellow oil. LCMS [ESI, M+1]: 463.9; ^1^H NMR (400 MHz, DMSO-*d_6_*) δ 9.68 (s, 1H), 7.88 (d, *J* = 8.0 Hz, 2H), 7.58 (d, *J* = 9.2 Hz, 2H), 7.30 - 7.19 (m, 2H), 5.24 (s, 2H), 3.46 (s, 3H); ^19^F NMR (377 MHz, DMSO-*d_6_*) δ 37.56, -111.35.

1-(3,5-difluoro-4-((4-((fluorosulfonyl)oxy)phenoxy)methyl)phenyl)-1H-1,2,4-triazole-3-carboxylic acid (**20j**)

Obtained as a white solid. LCMS [ESI, M+1]: 429.8; ^1^H NMR (400 MHz, DMSO-*d_6_*) δ 9.51 (s, 1H), 7.85 (d, *J* = 8.4 Hz, 2H), 7.58 (d, *J* = 9.2 Hz, 2H), 7.24 (d, *J* = 9.2 Hz, 2H), 5.23 (s, 2H); ^19^F NMR (377 MHz, DMSO-*d_6_*) δ 37.56, -111.58.

4-((4-(3-carbamoyl-1H-1,2,4-triazol-1-yl)-2,6-difluorobenzyl)oxy)phenyl sulfurofluoridate (**20k**)

Obtained as a gray solid. LCMS [ESI, M-1]: 429.0; ^1^H NMR (400 MHz, DMSO-*d_6_*) δ 9.49 (s, 1H), 8.07 (s, 1H), 7.91 - 7.84 (m, 2H), 7.80 (s, 1H), 7.57 (d, *J* = 9.0 Hz, 2H), 7.24 (d, J = 9.2 Hz, 2H), 5.22 (s, 2H); ^19^F NMR (377 MHz, DMSO-*d_6_*) δ 37.52, -111.76.

4-((4-(3-acetamido-1H-1,2,4-triazol-1-yl)-2,6-difluorobenzyl)oxy)phenyl sulfurofluoridate (**21a**)

Obtained as a gray solid. LCMS [ESI, M+1]: 443.0; ^1^H NMR (400 MHz, DMSO-*d_6_*) δ 10.68 (br s, 1H), 9.26 (s, 1H), 7.71 (d, *J* = 8.4 Hz, 2H), 7.57 (d, *J* = 9.2 Hz, 2H), 7.28 - 7.20 (m, 2H), 5.20 (s, 2H), 2.09 (s, 3H); ^19^F NMR (377 MHz, DMSO-*d_6_*) δ 37.54, -112.07.

*General Synthesis of 4-((2,6-difluoro-4-(3-(methylsulfonamido)-1H-1,2,4-triazol-1-yl)benzyl)oxy)phenyl sulfurofluoridate (****21b)***

**Step a:** A mixture of (4-((4-bromo-2,6-difluorobenzyl)oxy)phenoxy)(tert-butyl)dimethylsilane **22** (30 g, 69.87 mmol), bis(pinacolato)diboron (21.29 g, 83.84 mmol), potassium acetate (20.57 g, 209.61 mmol) in 1,4-dioxane (300 mL) was deoxygenated by purging argon over 15 min, then [1,1′-Bis(diphenylphosphino)ferrocene]dichloropalladium(II) (5.11 g, 6.987 mmol) was added and the reaction mixture was stirred at 100 °C for 3 h. After completion, the reaction mixture was cooled to room temperature, filtered through a pad of celite, washed with ethyl acetate (150 mL) and the filtrate was concentrated to give crude product. The crude product was then purified by column chromatography over silica gel (230-400 mesh) using 0-10% ethyl acetate in pet-ether to afford tert-butyl(4-((2,6-difluoro-4-(4,4,5,5-tetramethyl-1,3,2-dioxaborolan-2-yl)benzyl)oxy)phenoxy)dimethylsilane **23** (30 g, 90.12% yield) as an yellow gum. LCMS [ESI, M+1]: 477.3.

**Step b:** A mixture of tert-butyl(4-((2,6-difluoro-4-(4,4,5,5-tetramethyl-1,3,2-dioxaborolan-2-yl)benzyl)oxy)phenoxy)dimethylsilane **23** (30 g, 62.97 mmol), 3-nitro-1H-1,2,4-triazole **24** (14.36 g, 125.93 mmol), boric acid (7.79 g, 125.93 mmol), pyridine (19.92 g, 251.88 mmol) and molecular sieves (4 Å powder; 15 g) in acetonitrile (300 mL) was purged with oxygen for 15 min. Then copper(II) acetate (34.31 g, 188.91 mmol) was added and the mixture was stirred at 90 °C for 4 h. After completion, the reaction mixture was cooled to room temperature, diluted with ethyl acetate (150 mL), filtered through a pad of celite, washed the celite pad with ethyl acetate (150 mL) and the filtrate was concentrated to give crude product. The crude product was then purified by column chromatography over silica gel (230-400 mesh) using 10% ethyl acetate in pet-ether to afford 1-(4-((4-((tert-butyldimethylsilyl)oxy)phenoxy)methyl)-3,5-difluorophenyl)-3-nitro-1H-1,2,4-triazole **25** (15 g, 51.5% yield) as a pale yellow solid. LCMS [ESI, M+1]: 463.2.

**Step c**: Zinc dust (10.6 g, 162.15 mmol) was added to a mixture of 1-(4-((4-((tert-butyldimethylsilyl)oxy)phenoxy)methyl)-3,5-difluorophenyl)-3-nitro-1H-1,2,4-triazole **25** (15 g, 32.43 mmol) in tetrahydrofuran (75 mL) and methanol (75 mL), was added ammonium chloride (34.7 g, 648.6 mmol) in water (75 mL) at 0 °C, the resultant mixture was stirred at room temperature for 3 h. After completion, the reaction mixture was diluted with ethyl acetate (150 mL) and water (100 mL), stirred for 15 min, then filtered through a pad of celite. The celite pad was washed with ethyl acetate (150 mL), and the layers were separated and the aqueous layer was extracted with ethyl acetate (2 X 200 mL). The combined organic layer was dried over anhydrous sodium sulphate and evaporated to give crude product. The crude product was stirred with 10% ethyl acetate in pet-ether (200 mL), the resultant solid was filtered and dried to afford 1-(4-((4-((tert-butyldimethylsilyl)oxy)phenoxy)methyl)-3,5-difluorophenyl)-1H-1,2,4-triazol-3-amine **26** (9.1 g, 64.3%) as a pale yellow solid. LCMS [ESI, M+1]: 433.3; ^1^H NMR (400 MHz, DMSO-*d_6_*) δ 8.93 (s, 1 H), 7.54 (d, *J* = 8.80 Hz, 2 H), 6.92-6.90 (m, 2 H), 6.78-6.76 (m, 2 H), 5.90 (s, 2 H), 5.00 (s, 2 H), 0.93 (s, 9 H), 0.15 (s, 6 H); ^19^F NMR (400 MHz, DMSO-*d_6_*) δ -112.81.

**Step d**: Tetrabutylammonium fluoride 1.0 M tetrahydrofuran (29.4 mL, 24.5 mmol) was added to a suspension of 1-(4-((4-((tert-butyldimethylsilyl)oxy)phenoxy)methyl)-3,5-difluorophenyl)-1H-1,2,4-triazol-3-amine **26** (10.6 g, 24.5 mmol) in tetrahydrofuran (53 mL) at 0 °C and the reaction mixture was stirred at room temperature for 1 h. After completion, the reaction mixture was diluted with water (100 mL) and resulting mixture was extracted with ethyl acetate (2 X 200 mL). The combined organic layer was dried over anhydrous sodium sulphate and evaporated to give crude product. The crude product was then stirred with 10% ethyl acetate in pet-ether (200 mL), resultant solid was filtered and dried under vacumm to afford 4-((4-(3-amino-1H-1,2,4-triazol-1-yl)-2,6-difluorobenzyl)oxy)phenol **27** (7.7 g, 98.7%) off-white solid. LCMS [ESI, M+1]: 319.1; ^1^H NMR (400 MHz, DMSO-*d_6_*) δ ppm 9.00 (s, 1 H), 8.93 (s, 1 H), 7.53 (d, *J* = 8.80 Hz, 2 H), 6.83 (d, *J* = 6.80 Hz, 2 H), 6.68 (d, *J* = 8.80 Hz, 2 H), 5.89 (s, 2 H), 4.97 (s, 2 H); ^19^F NMR (400 MHz, DMSO-*d_6_*) δ = -112.81.

**Step e**: To a stirred suspension of 4-((4-(3-amino-1H-1,2,4-triazol-1-yl)-2,6-difluorobenzyl)oxy)phenol **27** (7.7 g, 24.19 mmol) in tetrahydrofuran (77 mL) was added 1,8-diazabicyclo[5.4.0]undec-7-ene (11 g, 72.57 mmol) followed by 4-(acetylamino)phenyl]imidodisulfuryl difluoride (9.12 g, 29.03 mmol) at 0 °C and the resultant mixture was stirred at 0 °C for 30 min. After completion, the reaction mixture was diluted with ice-cold water (500 mL) and stirred for 10 min. After 10 min, precipitate was filtered, washed with n-pentane (100 mL) and dried under vaccumm to afford 4-((4-(3-amino-1H-1,2,4-triazol-1-yl)-2,6-difluorobenzyl)oxy)phenyl sulfurofluoridate **28** (9.5 g, 98.1%) as a pale yellow solid. LCMS [ESI, M+1]: 401.2; ^1^H NMR (400 MHz, DMSO-*d_6_*) δ 8.94 (s, 1 H), 7.58-7.55 (m, 4 H), 7.22 (d, *J* = 9.20 Hz, 2 H), 5.91 (s, 2 H), 5.15 (s, 2 H); ^19^F NMR (400 MHz, DMSO-*d_6_*): 37.51, -112.67

**Step f**: To a stirred suspension of 4-((4-(3-amino-1H-1,2,4-triazol-1-yl)-2,6-difluorobenzyl)oxy)phenyl sulfurofluoridate **28** (9.5 g, 23.74 mmol) in dichloromethane (95 mL) was added triethylamine (16.54 mL, 118.68 mmol) followed by methanesulfonyl chloride (9.2 mL, 118.68 mmol) slowly at 0 °C and the resultant mixture was stirred at room temperature for 1 h. After completion, the mixture was diluted with ice cold water (200 mL) and extracted with dichloromethane (2 × 200 mL). The combined organic layer was dried over anhydrous sodium sulphate and evaporated under reduced pressure to give crude product. The crude product was then purified by column chromatography over silica gel (230-400 mesh) using 30-35% ethyl acetate in pet-ether as a gradient to afford 4-((2,6-difluoro-4-(3-(N-(methylsulfonyl)methylsulfonamido)-1H-1,2,4-triazol-1-yl)benzyl)oxy)phenyl sulfurofluoridate **29** (11.4 g, 86.4%) as an off-white solid. LCMS [ESI, M+1]: 557.0; ^1^H NMR (400 MHz, DMSO-*d_6_*) δ 9.55 (s, 1 H), 7.91 (d, *J* = 8 .4 Hz, 2 H), 7.57 (d, *J* = 9.20 Hz, 2 H), 7.24 (d, *J* = 9.20 Hz, 2 H), 5.23 (s, 2 H), 3.68 (s, 6 H); ^19^F NMR (400 MHz, DMSO-*d_6_*): 37.56, -111.44.

**Step g**: Tetrabutylammonium fluoride (1.0 M in tetrahydrofuran; 24.58 mL, 24.58 mmol) was added to a solution of 4-((2,6-difluoro-4-(3-(N-(methylsulfonyl)methylsulfonamido)-1H-1,2,4-triazol-1-yl)benzyl)oxy)phenyl sulfurofluoridate **29** (11.4 g, 20.48 mmol) in tetrahydrofuran (57 mL) at 0 °C and the resultant mixture was stirred at 0 °C for 1 h. After completion, the reaction mixture was diluted with water (100 mL) and the reaction mixture was extracted with ethyl acetate (3 × 150 mL). The combined organic layer was dried over sodium sulphate and evaporated to give crude product. The crude was then purified by column chromatography over silica gel using 65-70% ethyl acetate in pet-ether to afford 4-((2,6-difluoro-4-(3-(methylsulfonamido)-1H-1,2,4-triazol-1-yl)benzyl)oxy)phenyl sulfurofluoridate **21b** (4.6 g, 46.94%) as a white solid. LCMS [ESI, M+1]: 479.1; ^1^H NMR (400 MHz, DMSO-*d_6_*) δ 11.2 (s, 1 H), 9.28 (s, 1 H), 7.73 (d, *J* = 8.4 Hz, 2 H), 7.57 (d, *J* = 9.20 Hz, 2 H), 7.23 (d, *J* = 9.20 Hz, 2 H), 5.20 (s, 2 H), 3.32 (s, 3 H); ^13^C NMR (100 MHz, DMSO- *d_6_*) δ 163.30, 163.21, 160.82, 160.74, 158.33, 157.89, 144.09, 143.69, 139.04, 138.90, 138.76, 122.93, 116.87, 111.05, 110.86, 110.66, 103.18, 102.97, 102.88, 58.55, 41.72; ^19^F NMR (400 MHz, DMSO- *d_6_*): δ 37.54, -112.00; Elemental analysis: theoretical (C, 40.17%; H, 2.74%; N, 11.71%), found (C, 40.29%; H, 2.75%; N, 11.68%).

4-((2,6-difluoro-4-(3-sulfamoyl-1H-1,2,4-triazol-1-yl)benzyl)oxy)phenyl sulfurofluoridate (**20l**)

Obtained as a brown solid. LCMS [ESI, M+1]: 465.0. ^1^H NMR (400 MHz, DMSO-*d_6_*) δ 9.57 (s, 1H), 8.04 (s, 2H), 7.82 (d, *J* = 8.4 Hz, 2H), 7.58 (d, *J* = 9.2 Hz, 2H), 7.24 (d, *J* = 9.2 Hz, 2H), 5.23 (s, 2H); ^19^F NMR (376 MHz, DMSO- *d_6_*) δ 37.57, -111.44.

4-((2,6-difluoro-4-(3-(N-methylmethylsulfonamido)-1H-1,2,4-triazol-1-yl)benzyl)oxy)phenyl sulfurofluoridate (**21c**)

Obtained as an off white solid. LCMS [ESI, M+1]: 493.2; ^1^H NMR (400 MHz, CDCl_3_) δ 8.42 (s, 1H), 7.33-7.26 (m, 2H), 7.05 (m, 2H), 5.14 (s, 2H), 3.50 (s, 3H), 3.38 (s, 3H); ^19^F NMR (376 MHz, CDCl_3_) δ 36.62, -110.25, -110.27.

4-((4-(3-(ethylsulfonamido)-1H-1,2,4-triazol-1-yl)-2,6-difluorobenzyl)oxy)phenyl sulfurofluoridate (**21d**)


Obtained as an off white solid. LCMS [ESI, M+1]: 493.0; ^1^H NMR (400 MHz, DMSO- *d_6_*) δ 9.12 (s, 1H), 7.68 (d, *J* = 8.4 Hz, 2H), 7.57 (d, *J* = 9.2 Hz, 2H), 7.22 (d, *J* = 9.2 Hz, 2H), 5.29 (s, 2H), 3.41 (m, 2H), 3.23 (t, *J* = 7.2 Hz, 3 H); ^19^F NMR (376 MHz, DMSO- *d_6_*) δ 37.54, -112.25.

4-((2,6-difluoro-4-(3-((1-methylethyl)sulfonamido)-1H-1,2,4-triazol-1-yl)benzyl)oxy)phenyl sulfurofluoridate (**21e**)

Obtained as an off white solid. LCMS [ESI, M+1]: 507.2; ^1^H NMR (400 MHz, DMSO-*d_6_*) δ 10.99 (br s, 1H), 9.14 (s, 1H), 7.66 (d, *J* = 8.4 Hz, 2H), 7.57 (d, *J* = 8.8 Hz, 2H), 7.23 (m, 2H), 5.18 (s, 2H), 3.77 (m, 1H), 2.26 (d, *J* = 7.2 Hz, 3 H); ^19^F NMR (376 MHz, DMSO-*d_6_*) δ 37.54, -112.32.

4-((4-(3-((1,1-dimethylethyl)sulfonamido)-1H-1,2,4-triazol-1-yl)-2,6-difluorobenzyl)oxy)phenyl sulfurofluoridate (**21f**)

Obtained as an off white solid. LCMS [ESI, M+1]: 521.2; ^1^H NMR (400 MHz, DMSO-*d_6_*) δ 10.62 (s, 1H), 9.22 (s, 3H), 7.68 (d, *J* = 8.4 Hz, 2H), 7.57 (d, *J* = 9.2 Hz, 2H), 7.23 (d, *J* = 9.2 Hz, 2H), 5.19 (s, 2H), 1.39 (s, 9H); ^19^F NMR (376 MHz, DMSO-*d_6_*) δ 37.57, -111.79.

**Biological assay protocols**

**MIC determination in Mtb**

The minimum inhibitory concentration (MIC) of the test compounds were determined using ten-point, two-fold serial dilutions in 7H9 broth supplemented with 10% OADC and glycerol using Microdilution Alamar Blue Assay (MABA) as previously^1,2^.  Briefly, Mycobacterial cells (H37Rv and CDC1551) at the mid-logarithmic phase of growth were diluted (1:1,000). Twenty µl of this dilution was added to each well containing 2-fold serially diluted test compounds and incubated for 7 days at 37 °C.  Alamar Blue (Invitrogen) reagent (4 µL per well) was added along with 20% Tween 80 (2.5 µL per well, Sigma Aldrich) to evaluate bacterial cell viability.  Plates were read 24 h after adding Alamar Blue at absorbance 570 nm with a reference wavelength of 600 nm.

**IC_50_ Determination in Mtb H37Ra**

Inhibition concentration of 50% was determined against Mtb H37Ra in liquid culture using an Alamar Blue reduction assay was used as described (VanderVen et al., 2015). For inhibition assays in Mtb was first cultured to an (OD600 of 0.4) in 7H9 media (7H9 base supplemented with 10% OADC and glycerol) and 0.05% tyloxapol. 1.0´106 bacteria were added to 384-well microplates containing 7H9 media to a final volume of 20 μL containing the experimental compounds or controls. Compounds were tested in a 9-point dose-titration, serially diluted 1:3 starting at 30 μM. The microplates were incubated for 7 days in humidified, sealed plastic bags at 37°C. To quantify bacterial proliferation 10 μL of an Alamar Blue solution 50% was added to each well and the plates were re-incubated at 37°C for 16 hr. Alamar Blue reduction was quantified using a PHERAstar plate reader (BMG Labtech) with λex = 492 nm and λem = 595 nm. All assay plates contained DMSO and 10 μM rifampicin control wells and percent inhibition for the experimental compounds was calculated. IC50 and IC90 values were determined by using the Smart Fit function in Genedata Screener Analyzer (v16.0.8) with the maximum % activity and the minimum % activity fixed at 100% and 0%, respectively.

**HEK293T cytotoxicity assay**

HEK293T (ATCC) were grown in DMEM (Gibco Cat # 11965-092) supplemented with 10% heat-inactivated FBS, Pen Strep (Gibco) 1X in a 5% CO_2_ humidified atmosphere at 37◦C. Cells were washed once with DPBS 1X (Gibco), incubated for less than 5 minutes at room temperature in TrypLE solution (Gibco), washed once in propagation medium to remove trypsin, and counted with Trypan Blue. 5 µL of cell suspension (7.5x10^4^ cells/well) was dispensed into 1536-well plates containing serial 3-fold drug dilution ranging from 40 µM to 2 nM and incubated for 3 days in a 5% CO_2_ humidified atmosphere at 37 ◦C. Cell Titer-Glo® Luminescent Cell Viability Assay (Promega) reagent was diluted 1:1 with water and then 2 µL were added to plates. Luminescence was measured in a PHERAStar plate reader (BMG Labtech) and data normalized to negative control (DMSO) minus inhibitor (Puromycin 10 µM). Dose-response curves were fit using the Smart Fit function in Genedata Screener Analyzer (v16.0.8).

**HEPG2 CYTOTOXICITY ASSAY**

HepG2 (ATCC) were grown in DMEM (Gibco Cat # 31053-028) supplemented with 10% heat-inactivated FBS, Pen Strep (Gibco) 1×, Glutamax 1× (Gibco), Sodium pyruvate 1 mM (Gibco) in a 5% CO_2_ humidified atmosphere at 37 ◦C. Cells were washed twice with DPBS 1× (Gibco), incubated ten minutes at 37 ◦C in TrypLE solution (Gibco), washed once in propagation medium to remove trypsin and counted with Trypan Blue. 5 µL of cell suspension (5.0×10^4^ cells/well) was dispensed into 1536-well plates, containing serial 3-fold drug dilution ranging from 40µM to 2 nM and incubated for 3 days in a 5% CO_2_ humidified atmosphere at 37 ◦C. Cell Titer-Glo® Luminescent Cell Viability Assay (Promega) reagent was diluted 1:1 with water and then 2 µL were added to plates. Luminescence was measured in a PHERAStar plate reader (BMG Labtech) and data normalized to negative control (DMSO) minus inhibitor (Puromycin 10µM). Dose-response curves were fit using the Smart Fit function in Genedata Screener Analyzer (v16.0.8)

Reference:

1. S. Cho, H. S. Lee, S. Franzblau. Microplate Alamar Blue Assay (MABA) and Low Oxygen Recovery Assay (LORA) for Mycobacterium tuberculosis. *Methods Mol. Biol.*

2015; 1285, 281-292. doi: 10.1007/978-1-4939-2450-9_17.

1. B. C. VanderVen, R. J. Fahey, W. Lee, Y. C. Liu, R. B. Abramovitch, C. Memmott, A. M. Crowe, L. D. Eltis, E. Perola, D. Deininger, T. S. Wang, C. P. Locher, D. G. Russell. Novel Inhibitors of Cholesterol Degradation in Mycobacterium tuberculosis Reveal How the Bacterium’s Metabolism Is Constrained by the Intracellular Environment. *PLOS Pathogen*. 2015; DOI:10.1371/journal.ppat.1004679

^1^H and ^19^F NMR of compound **3d**


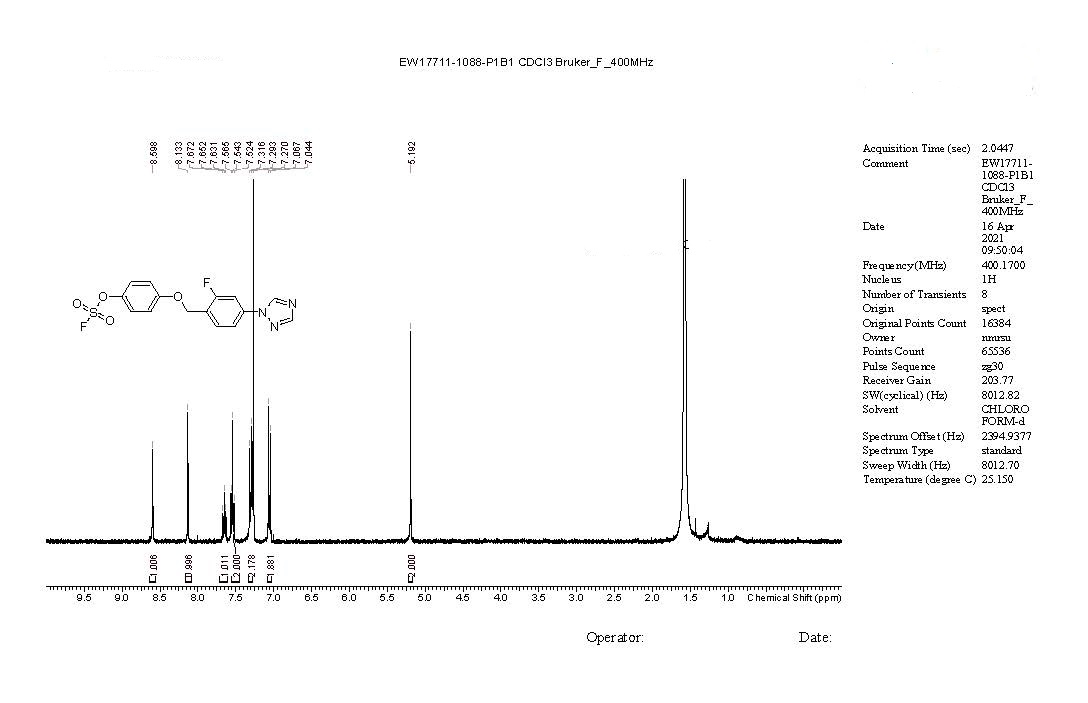


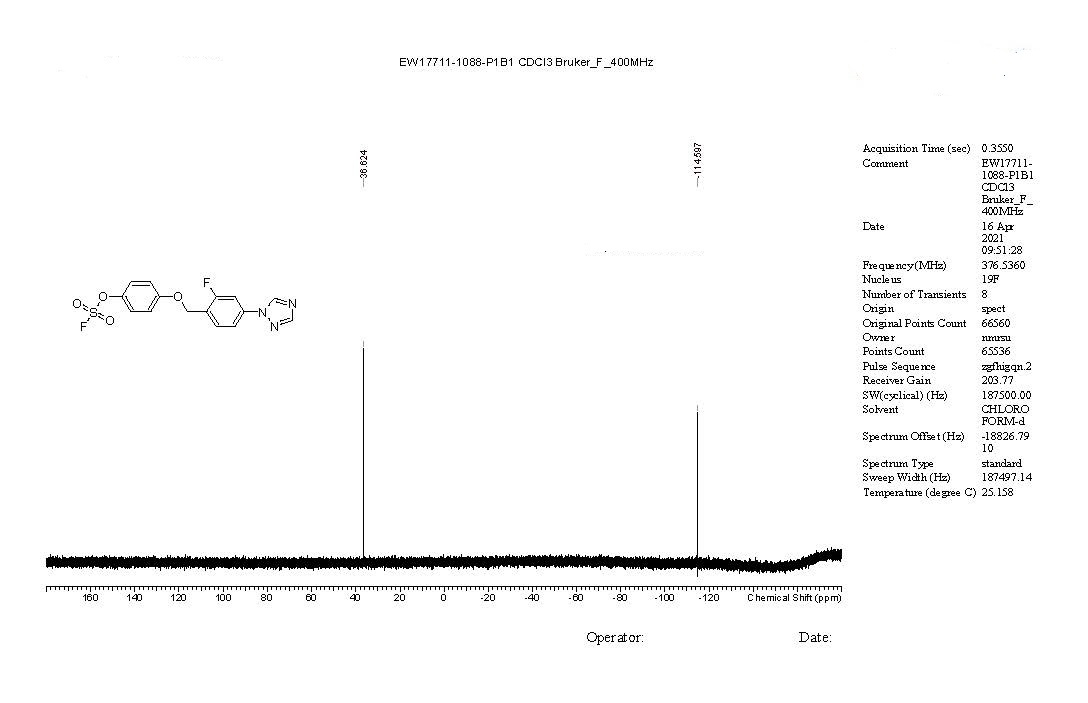


^1^H NMR of compound **3a**


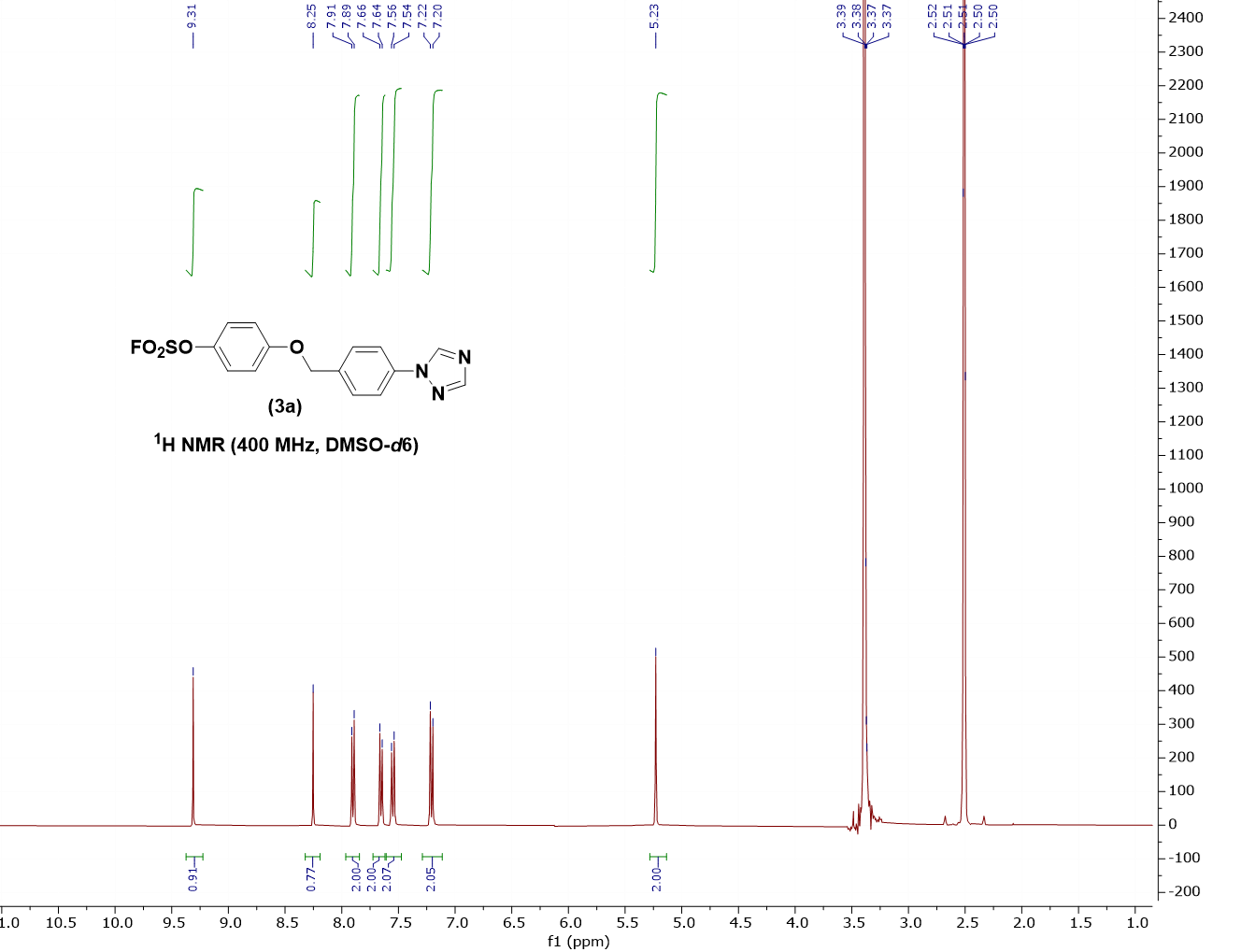


^1^H NMR of compound **3b**


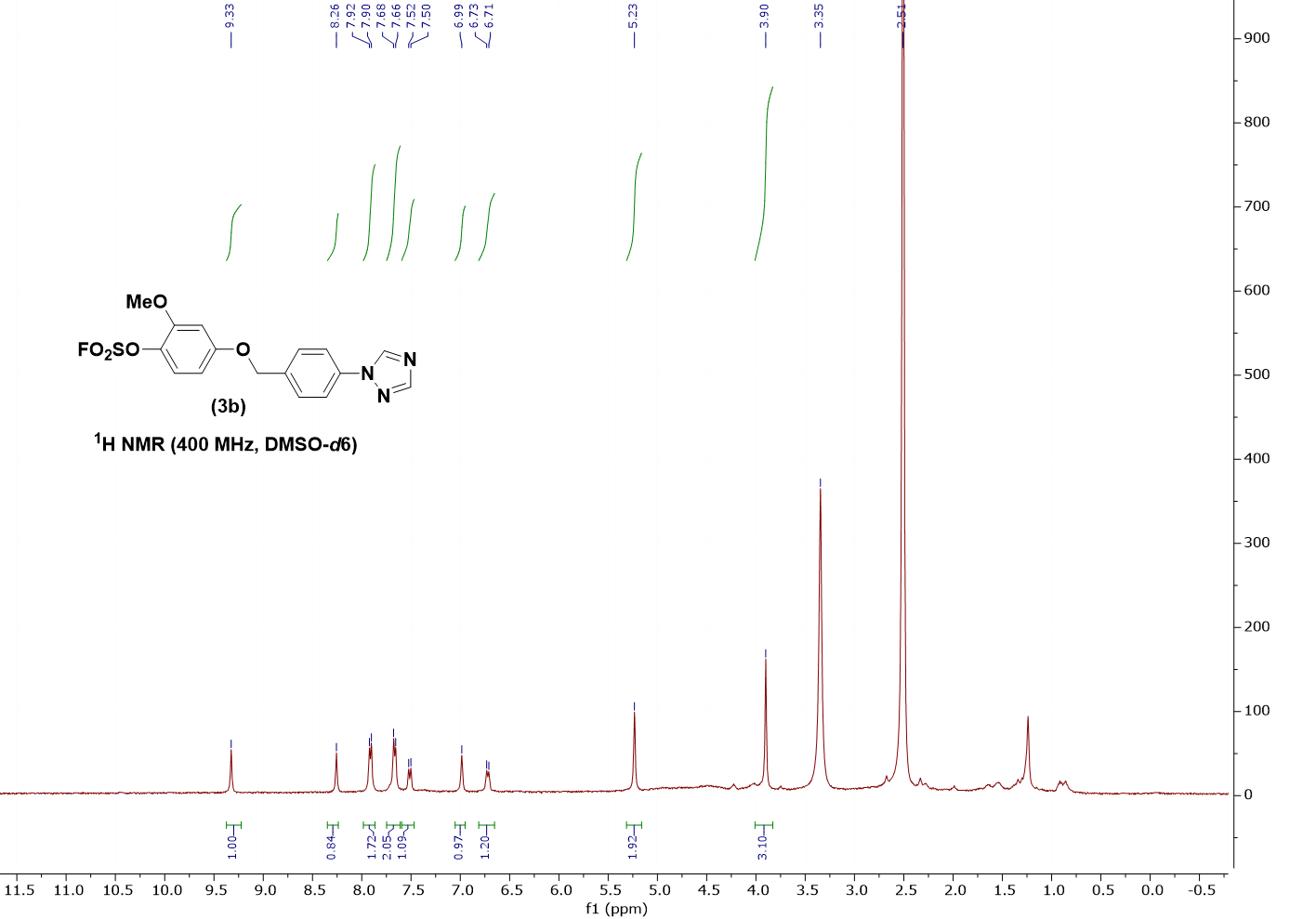


^1^H NMR of compound **3c**


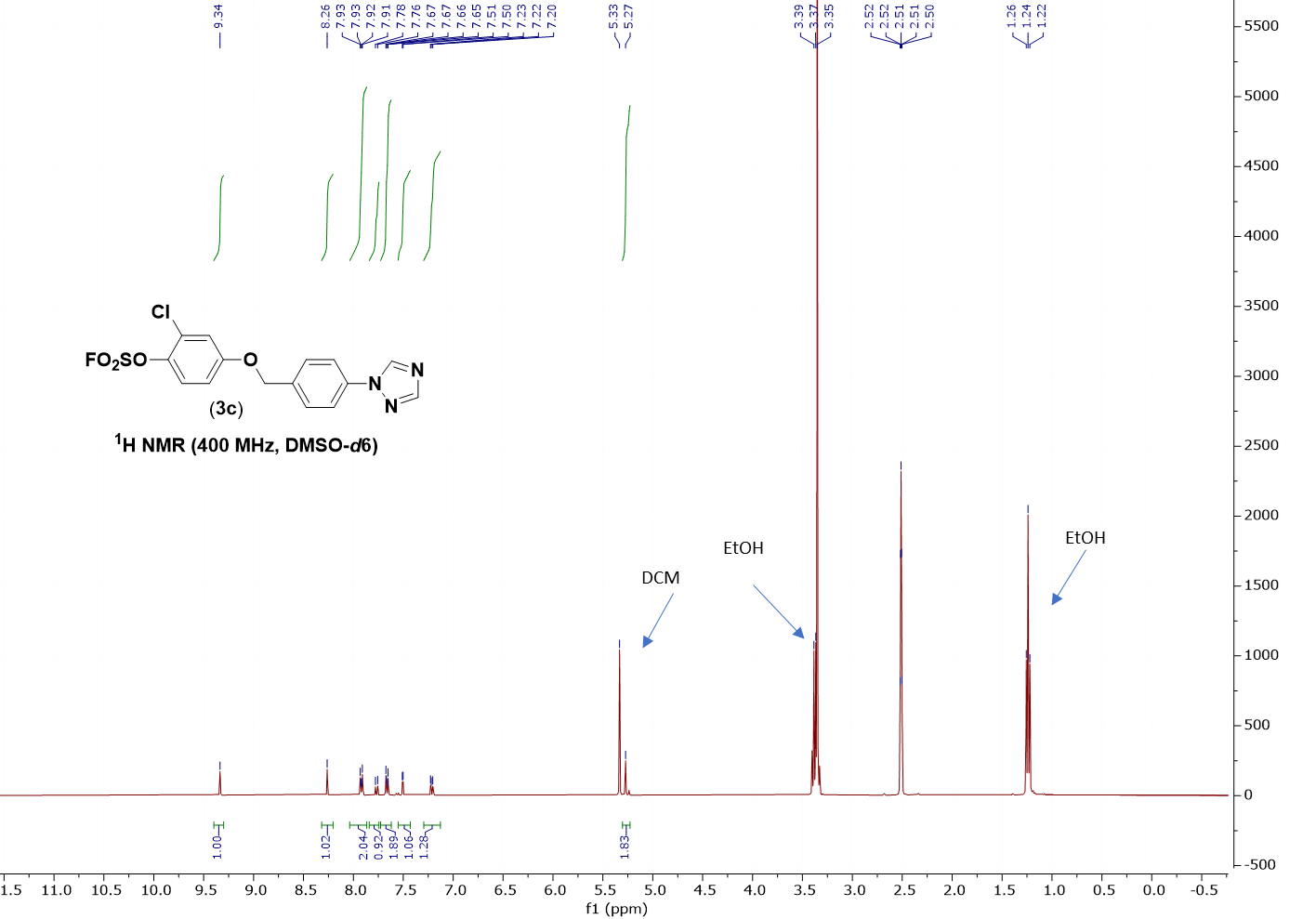


^1^H and ^19^F NMR of compound **3e**


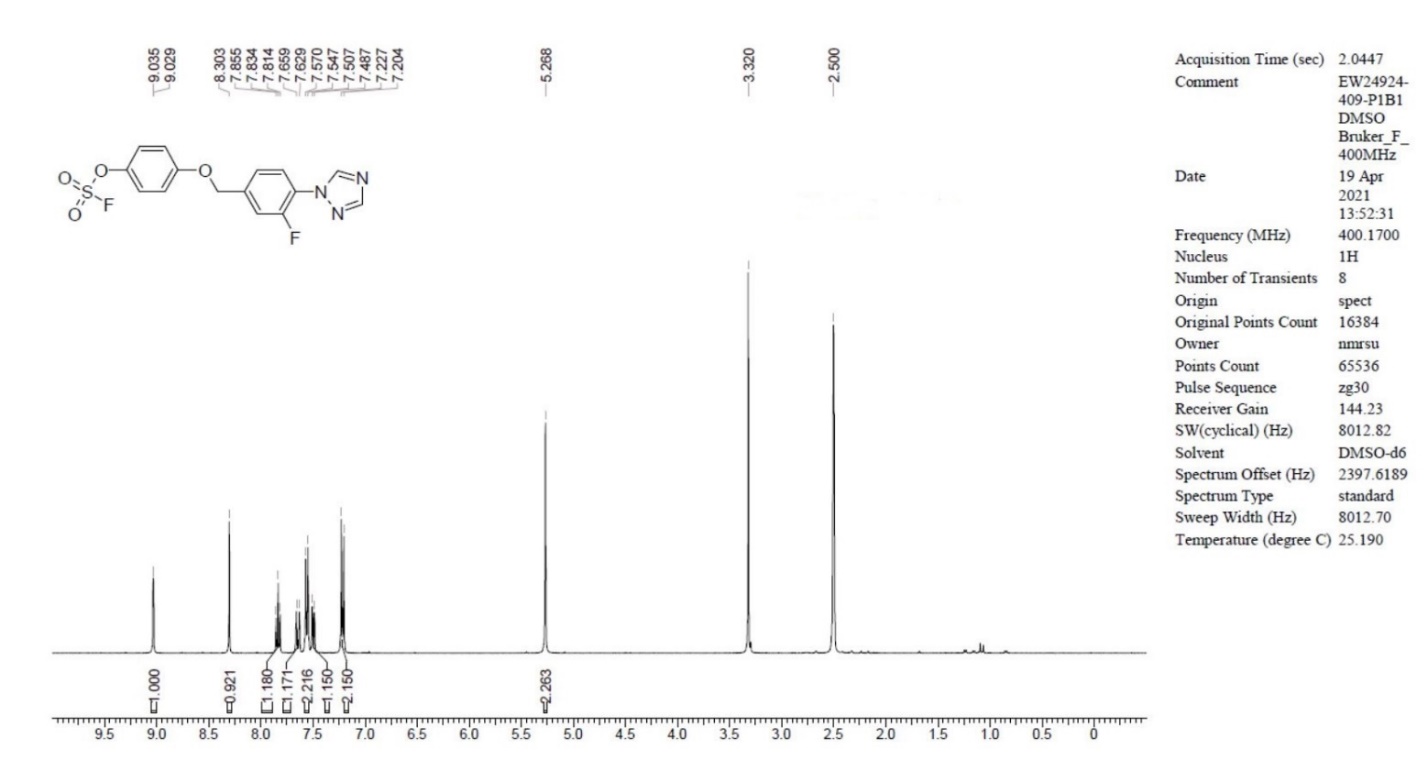


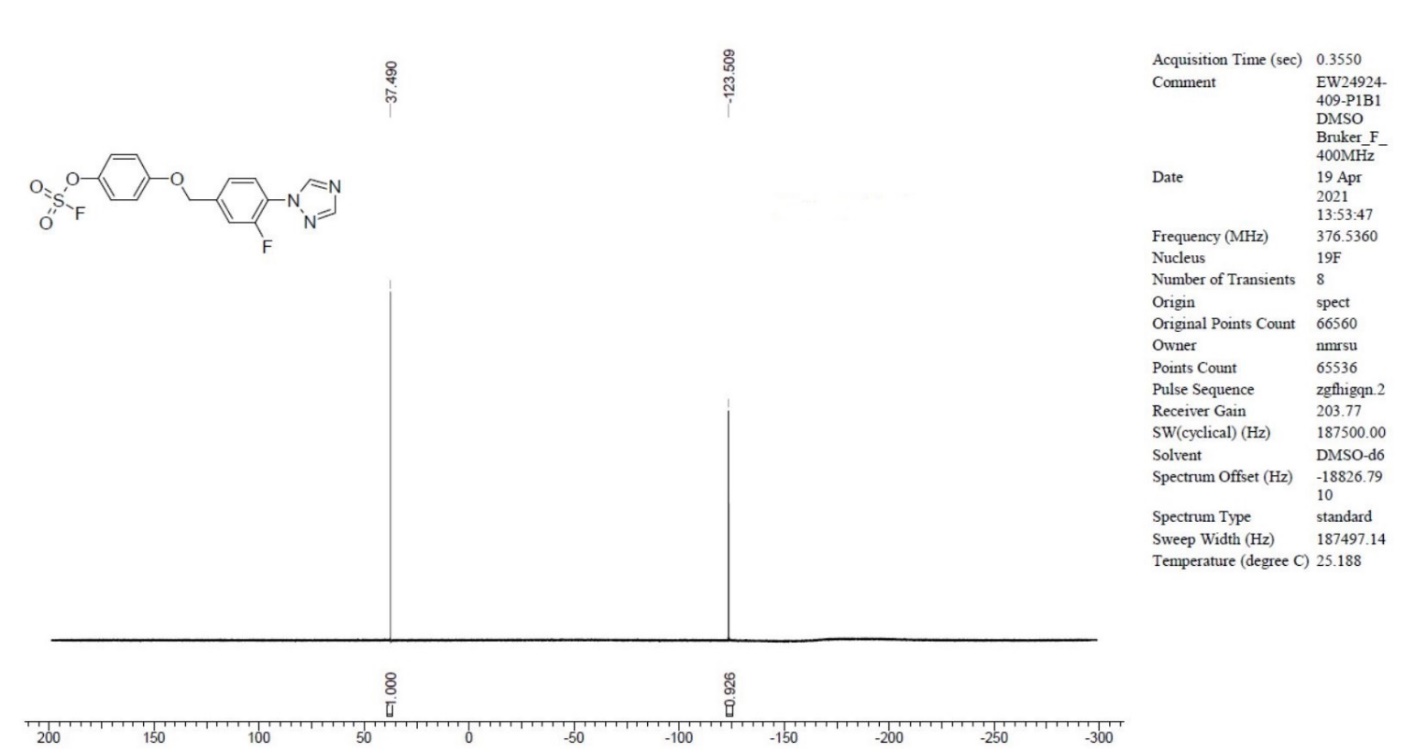


^1^H and ^19^F NMR of compound **3f**


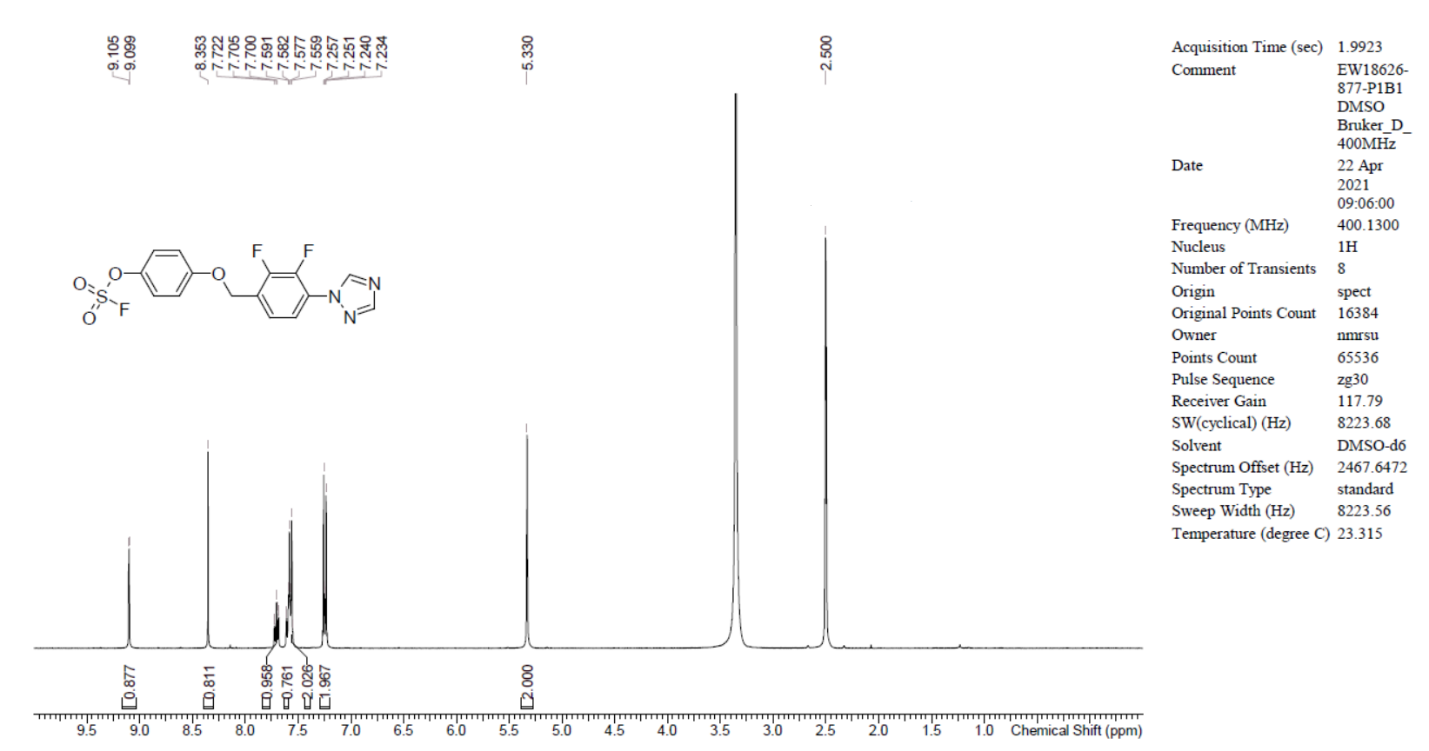


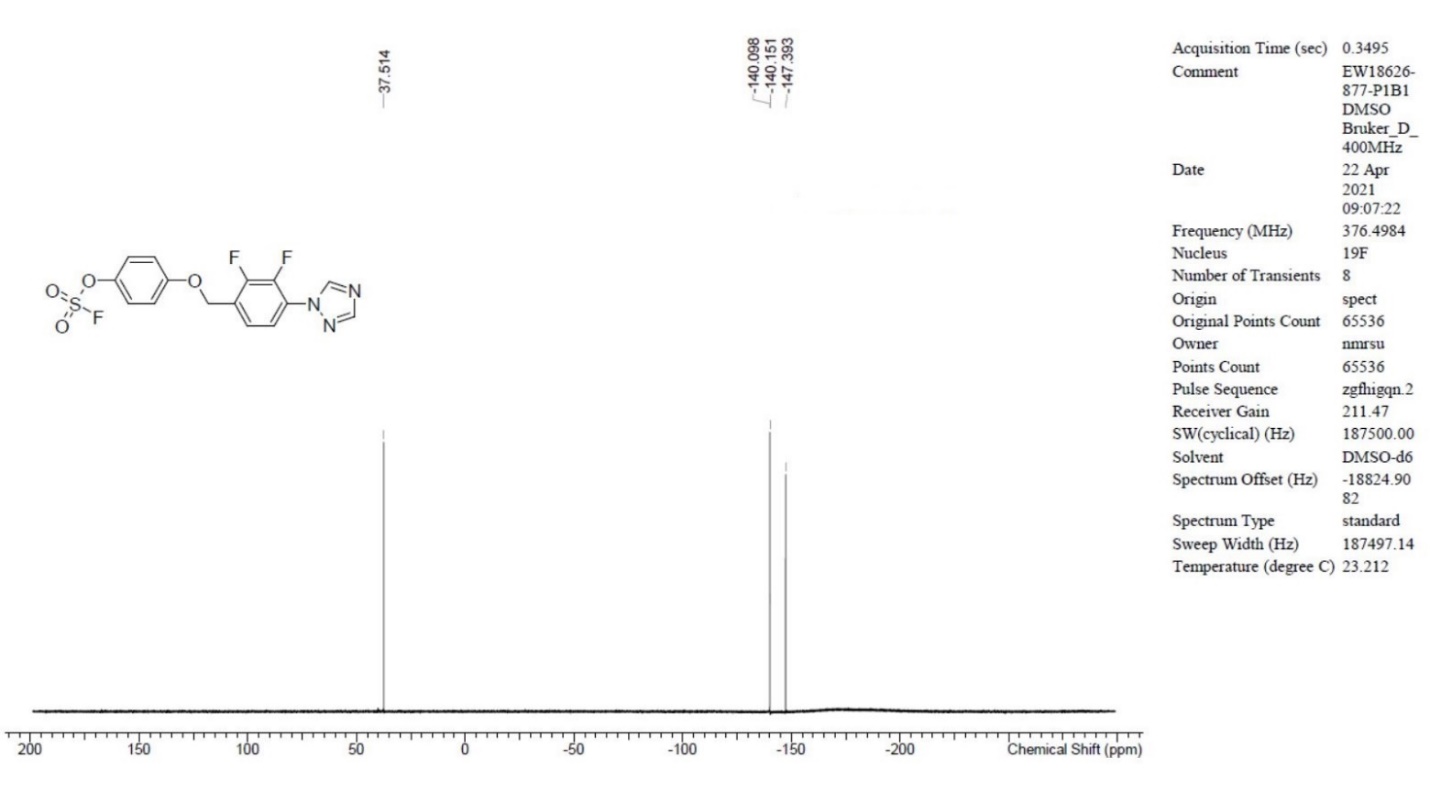


^1^H and ^19^F NMR of compound **3g**


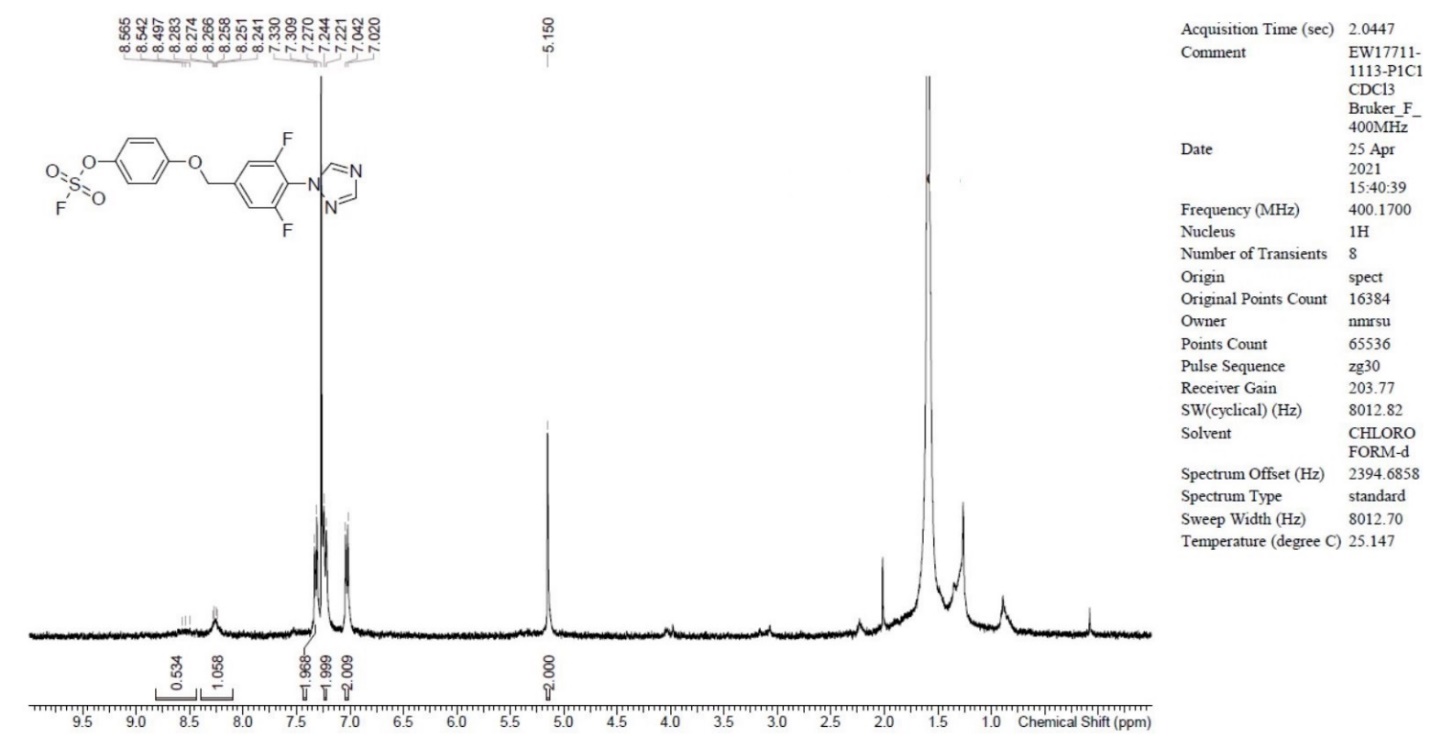


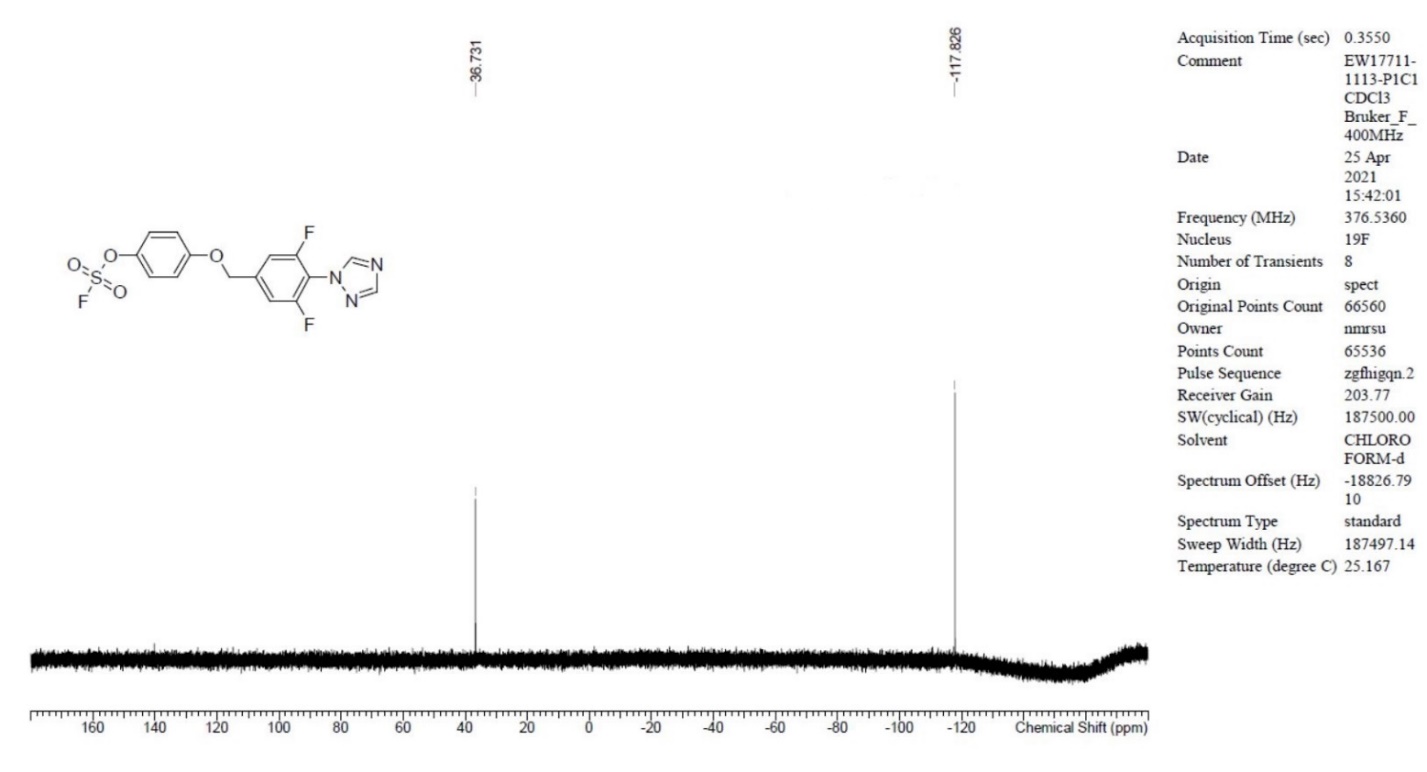


^1^H and ^19^F NMR of compound **3h**


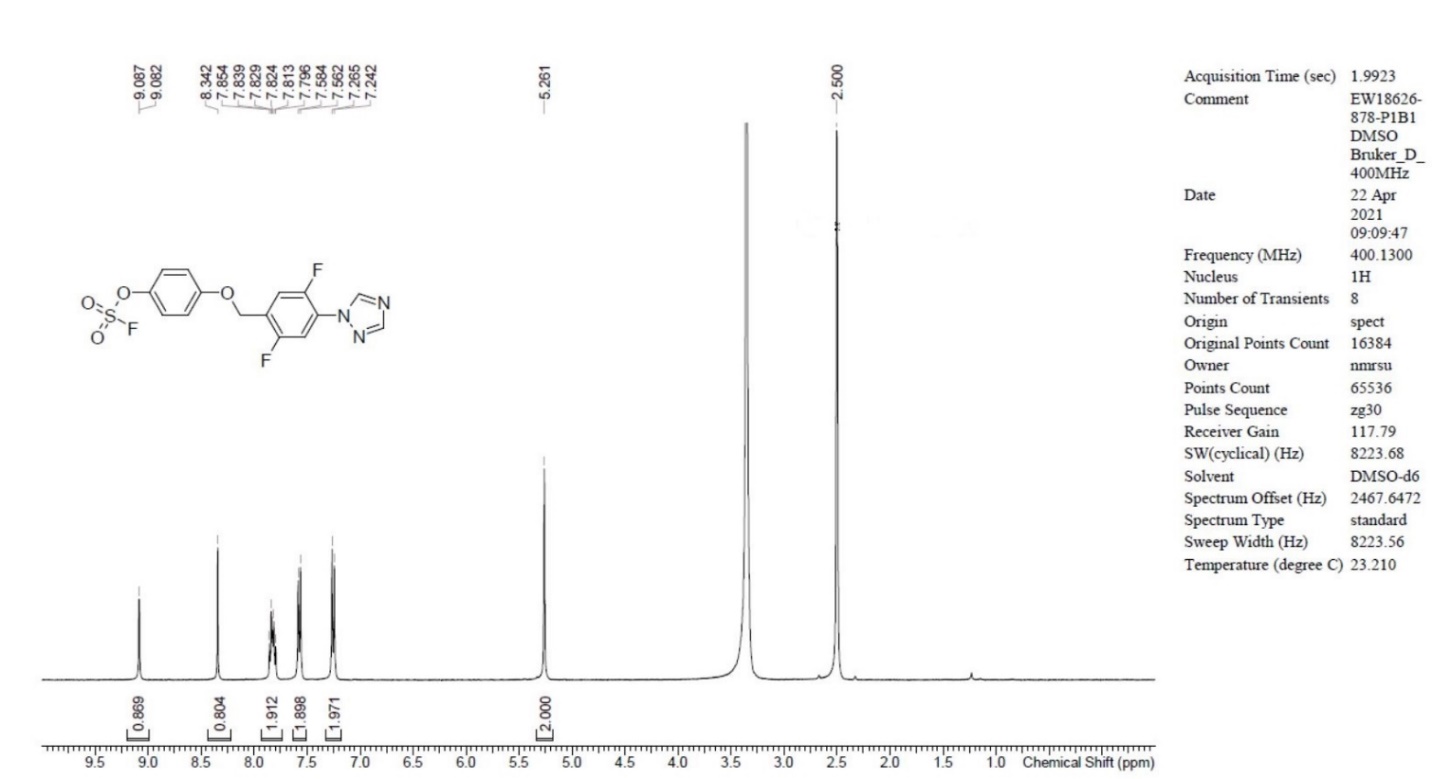


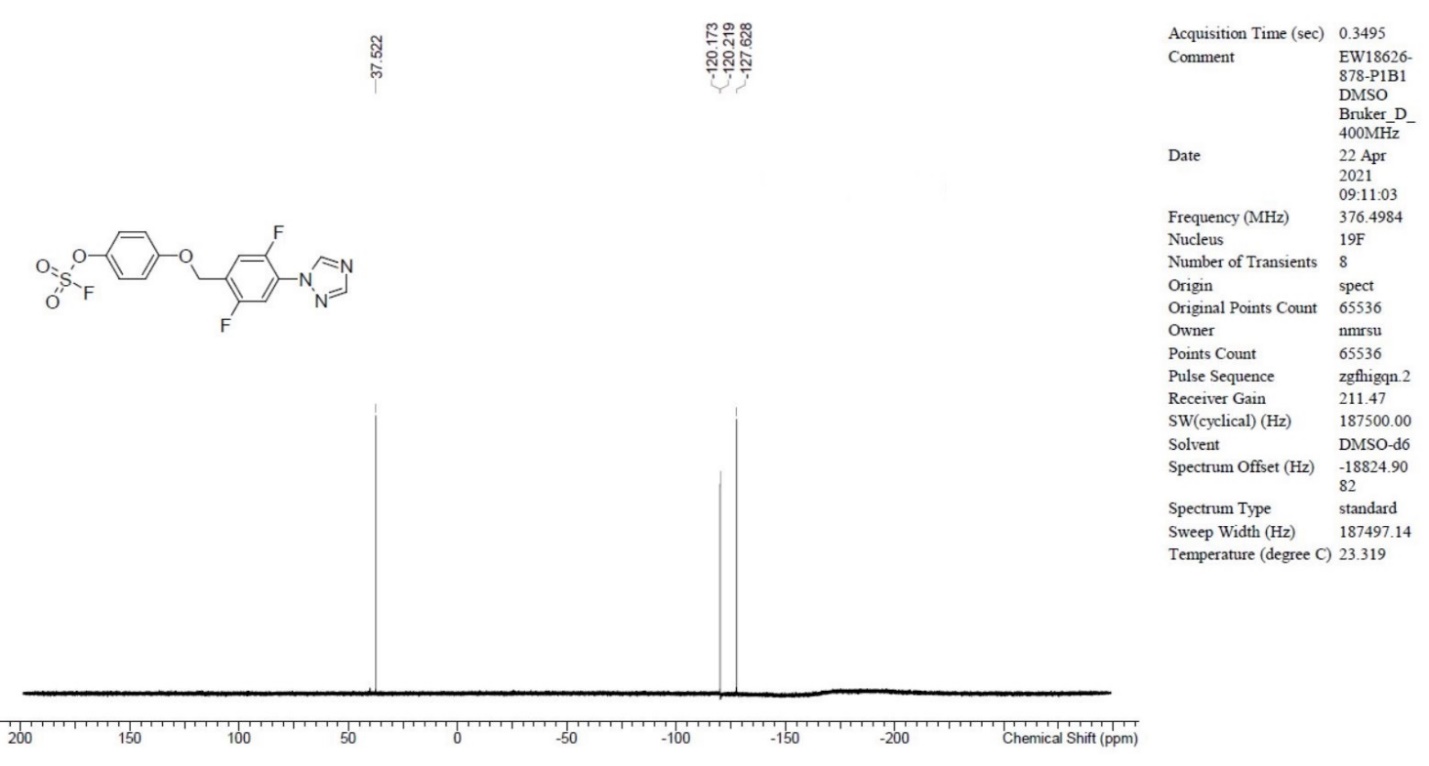


^1^H and ^19^F NMR of compound **3i**


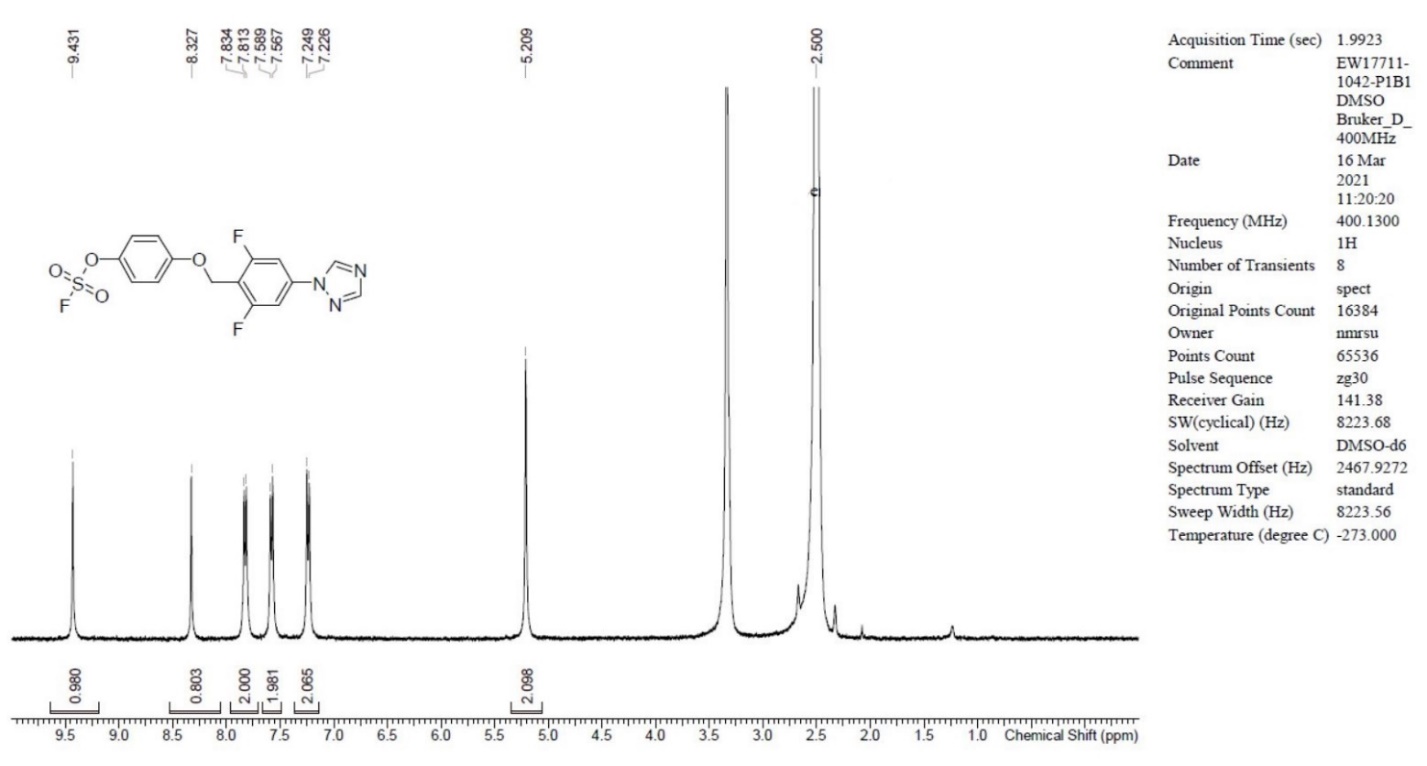


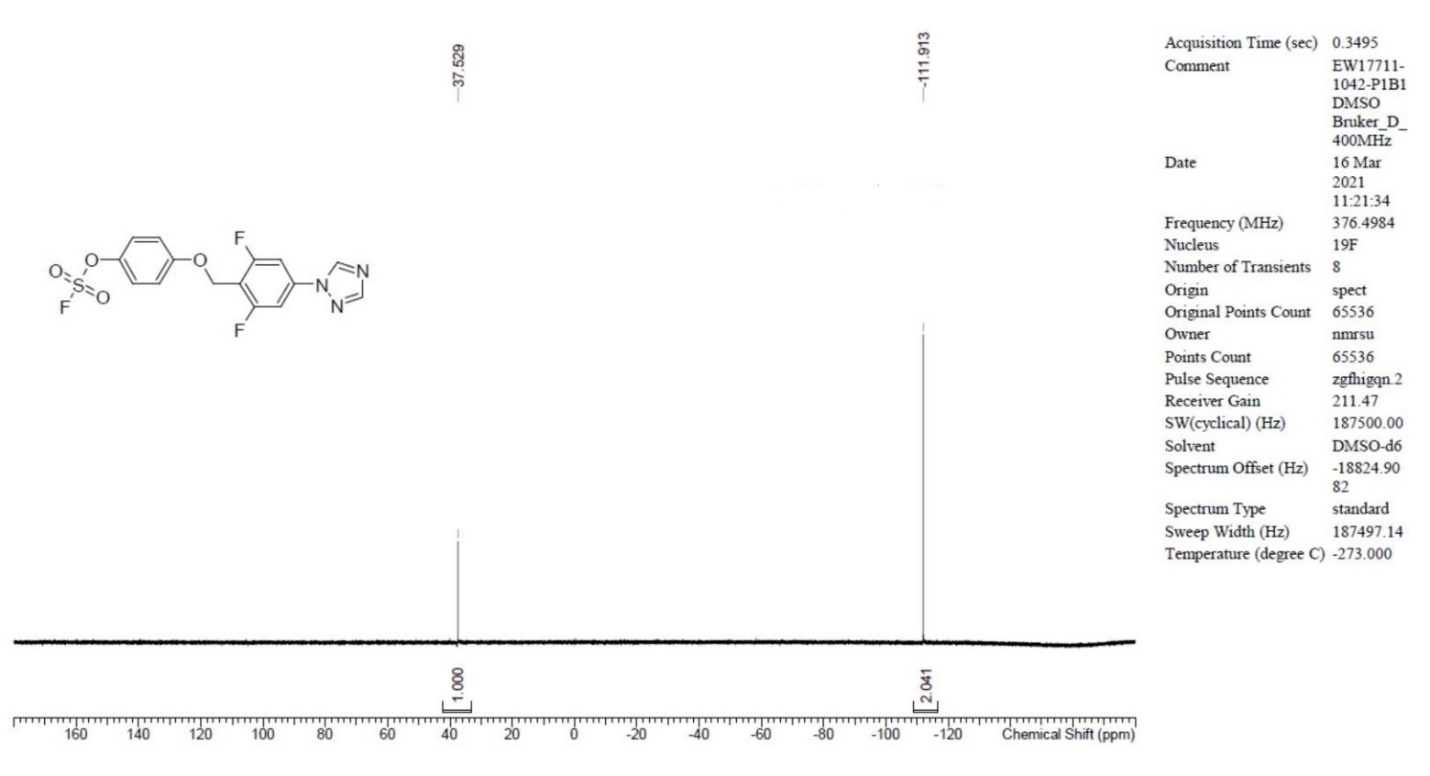


^1^H and ^19^F NMR of compound **3j**


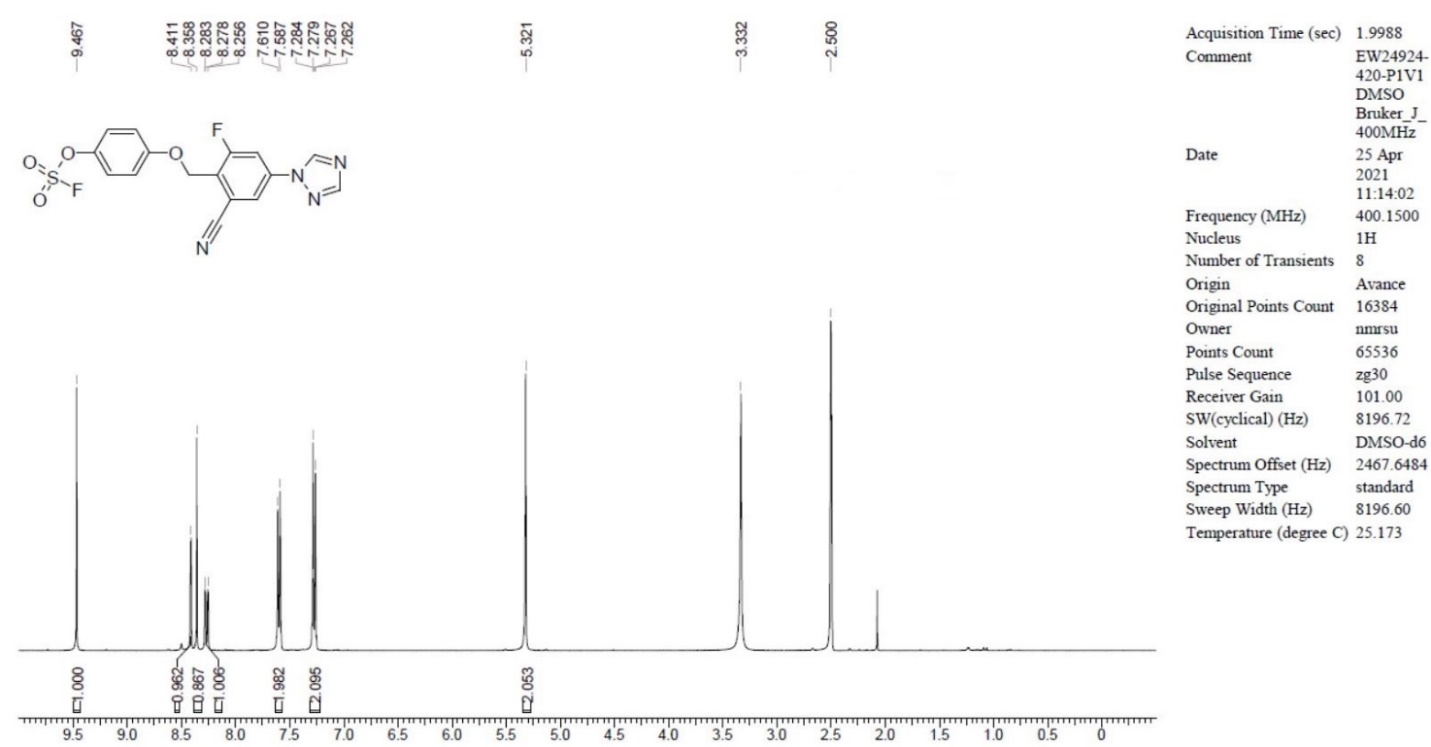


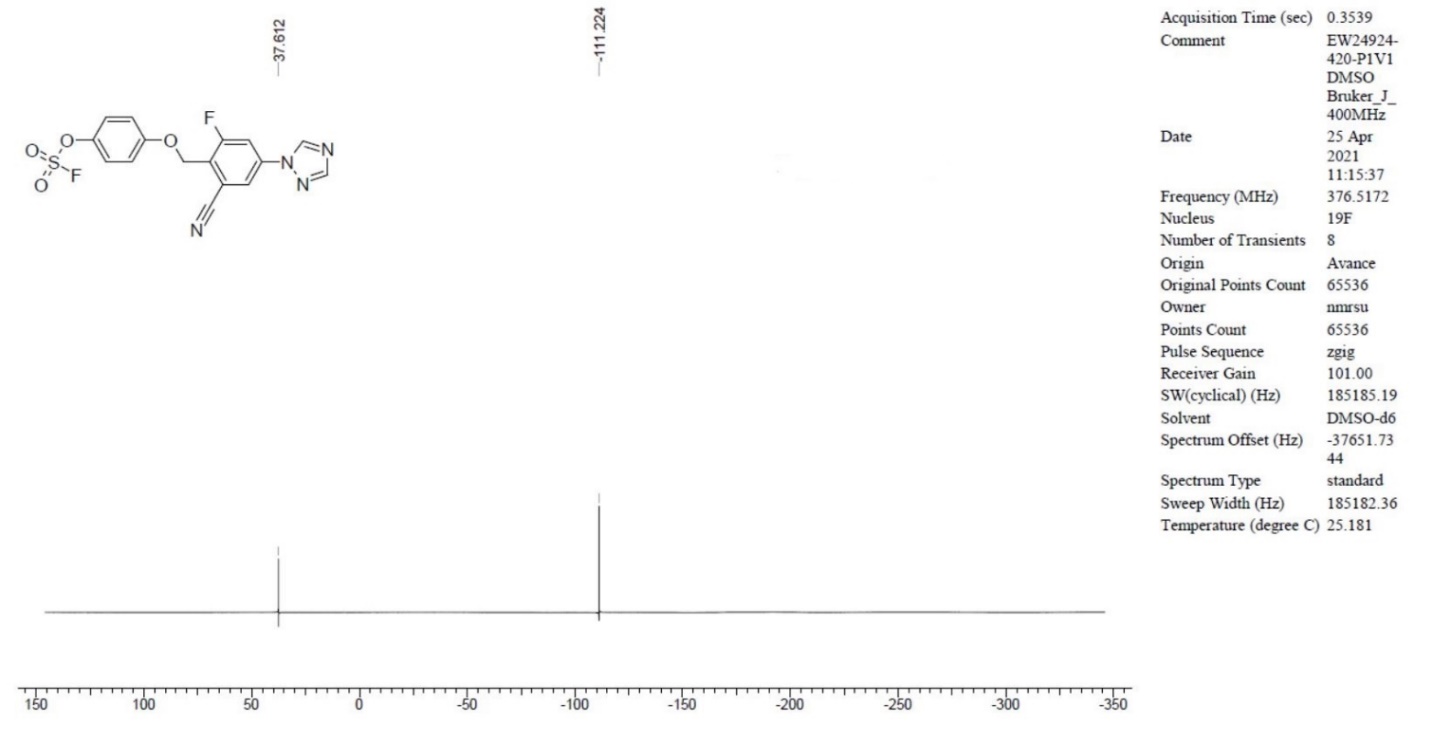


^1^H and ^19^F NMR of compound **3k**


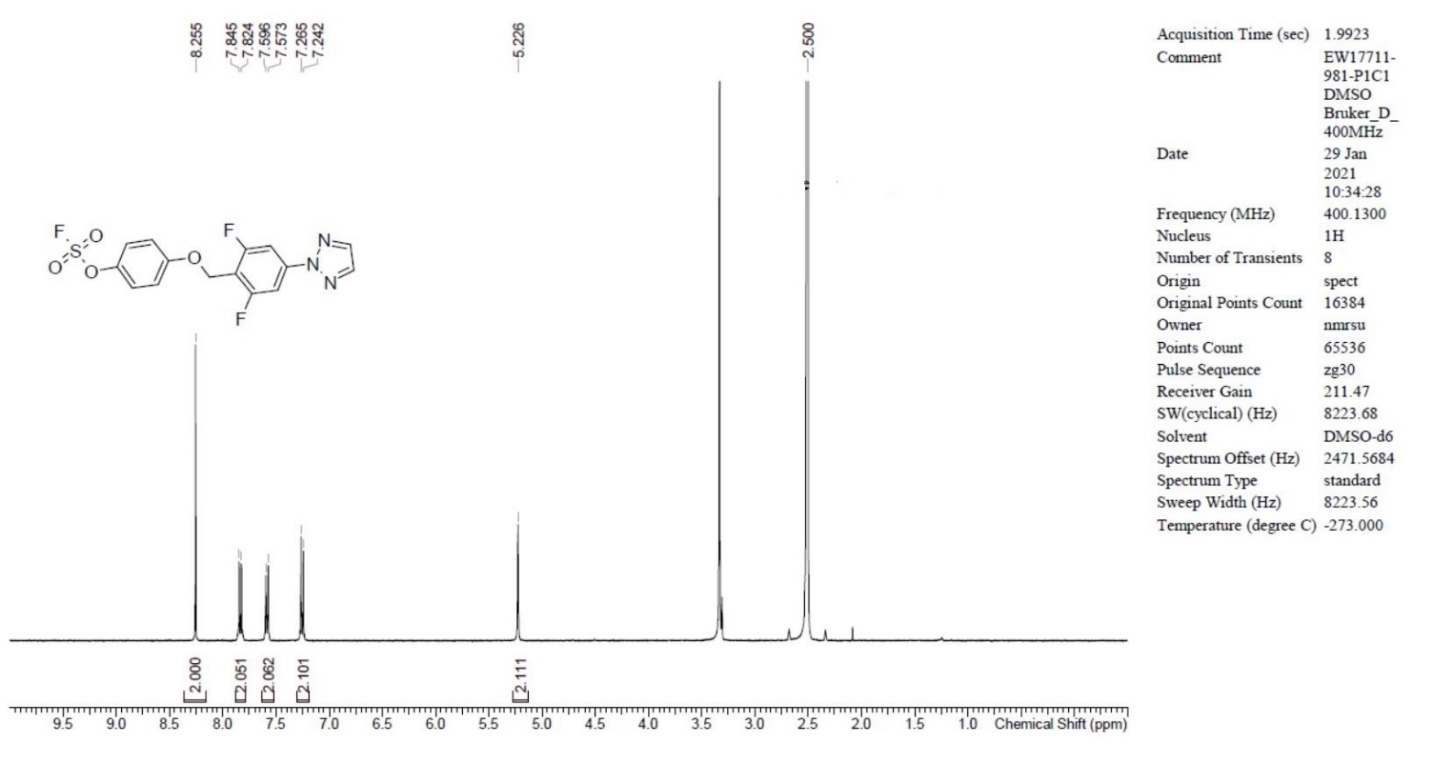


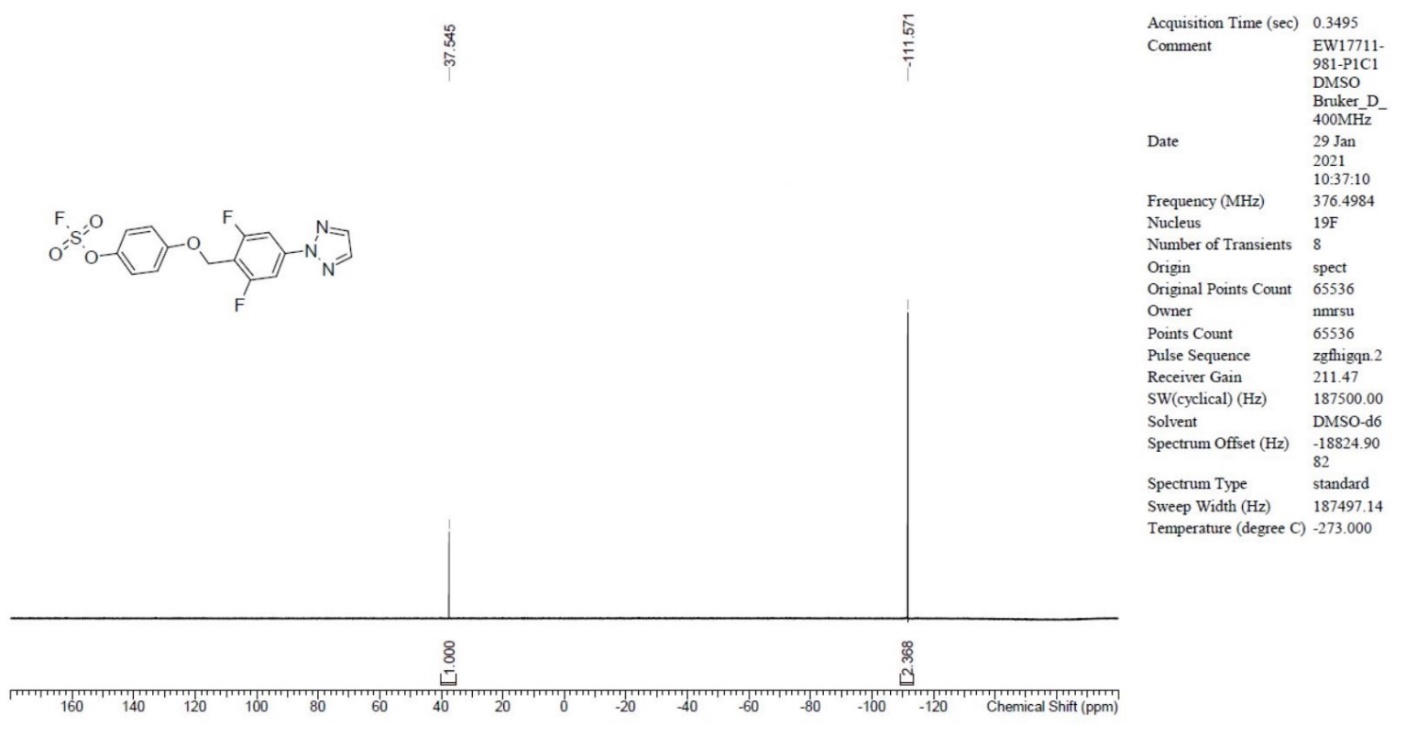


^1^H and ^19^F NMR of compound **3l**


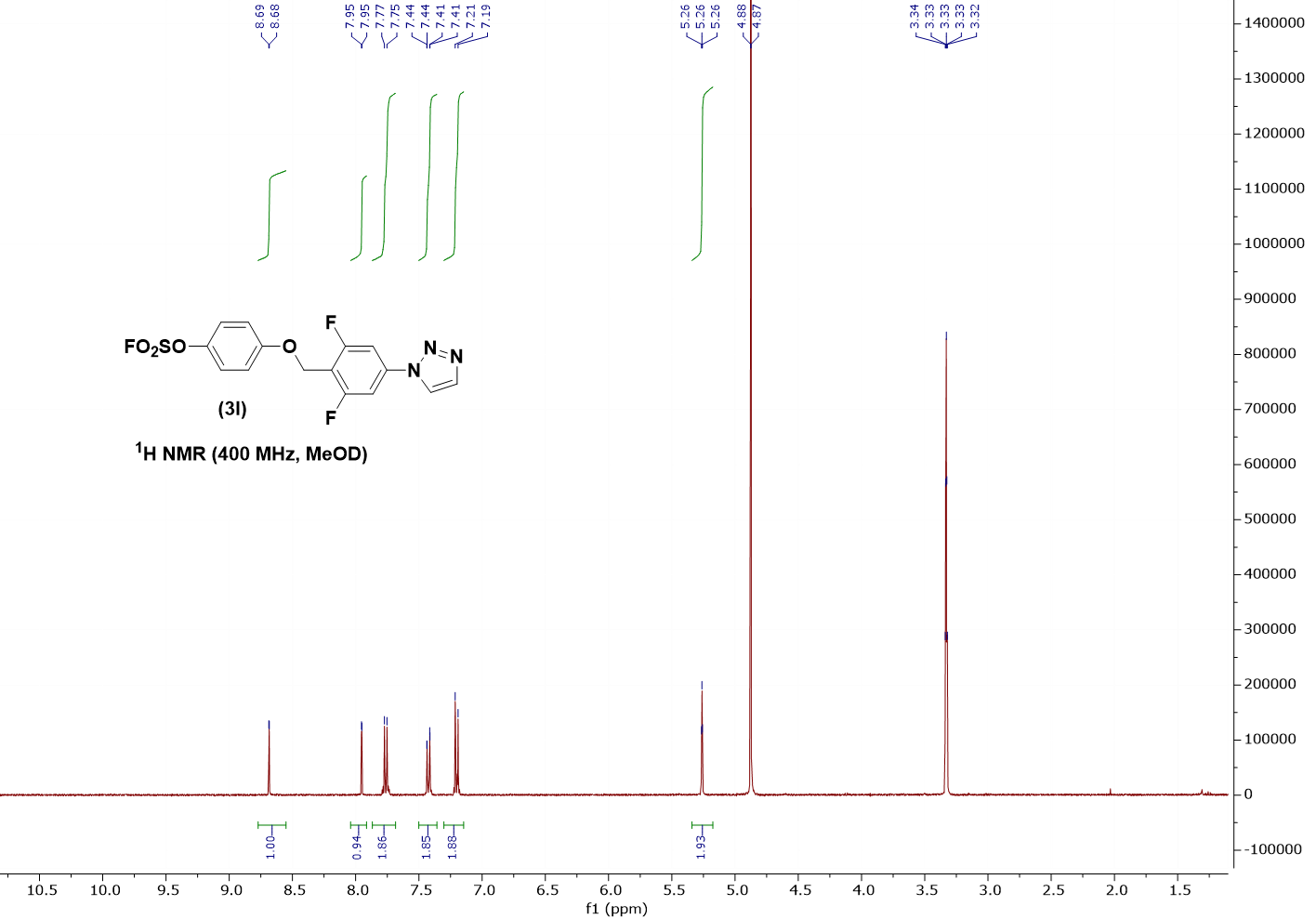


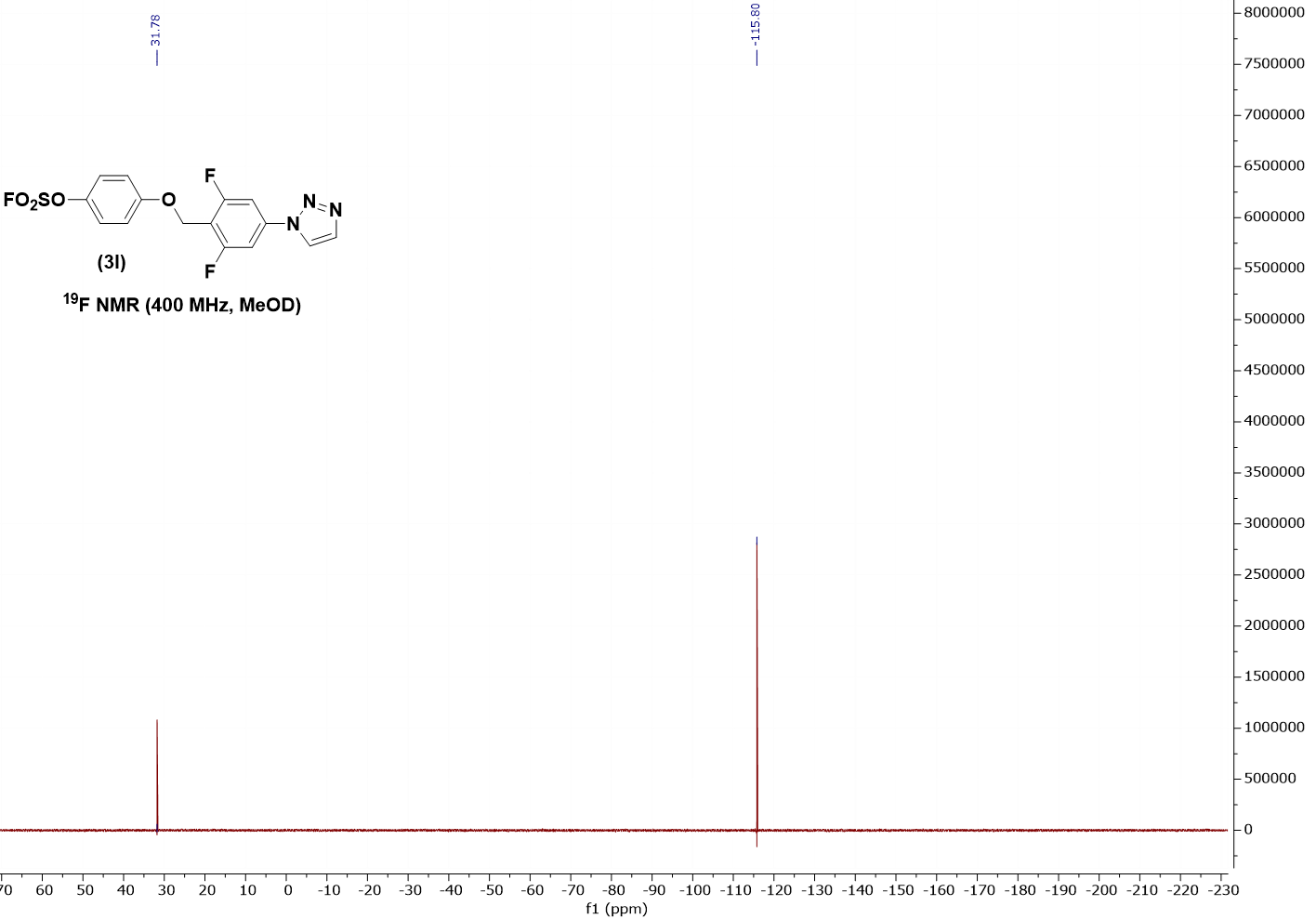


^1^H and ^19^F NMR of compound **3m**


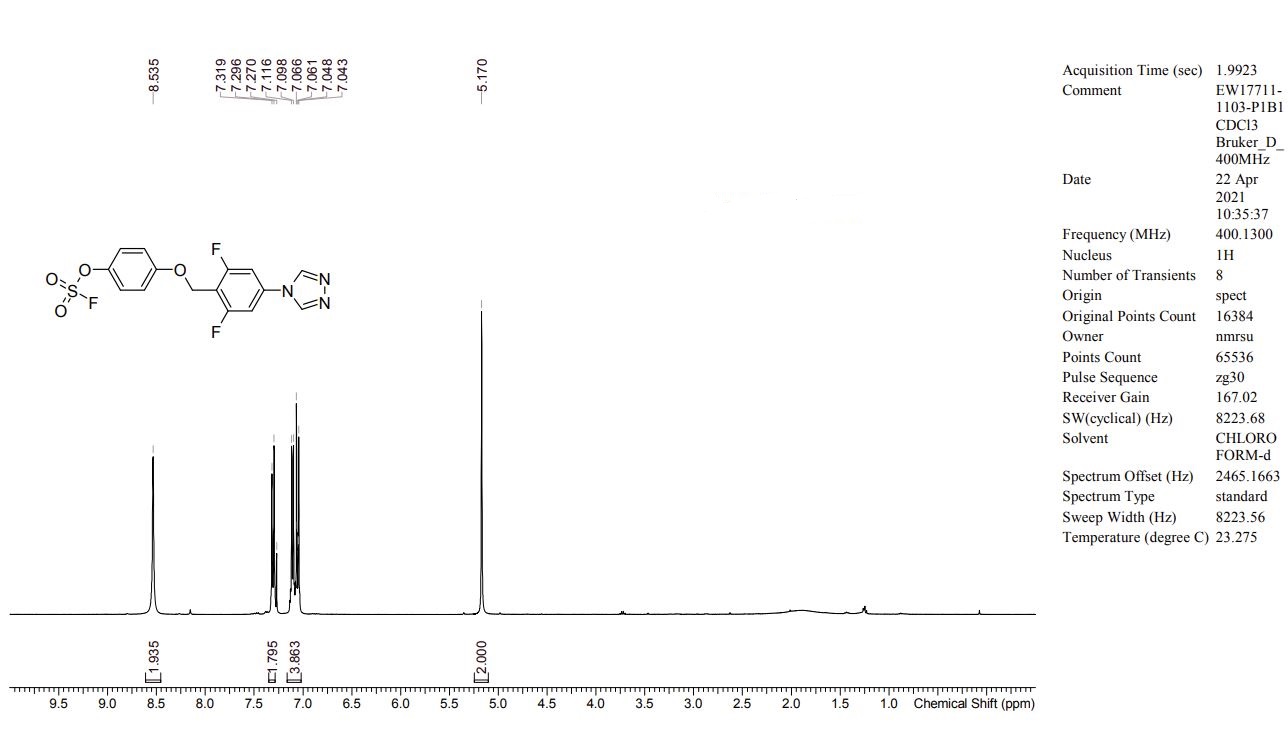


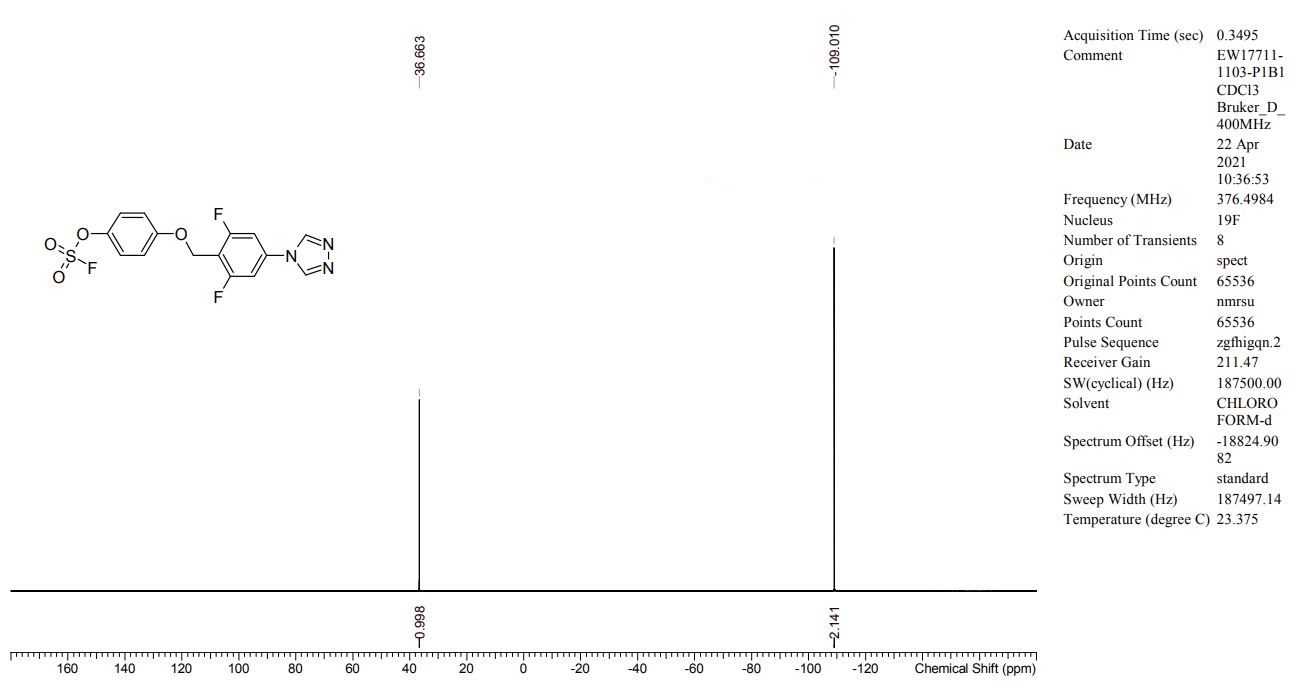


^1^H and ^19^F NMR of compound **19a**


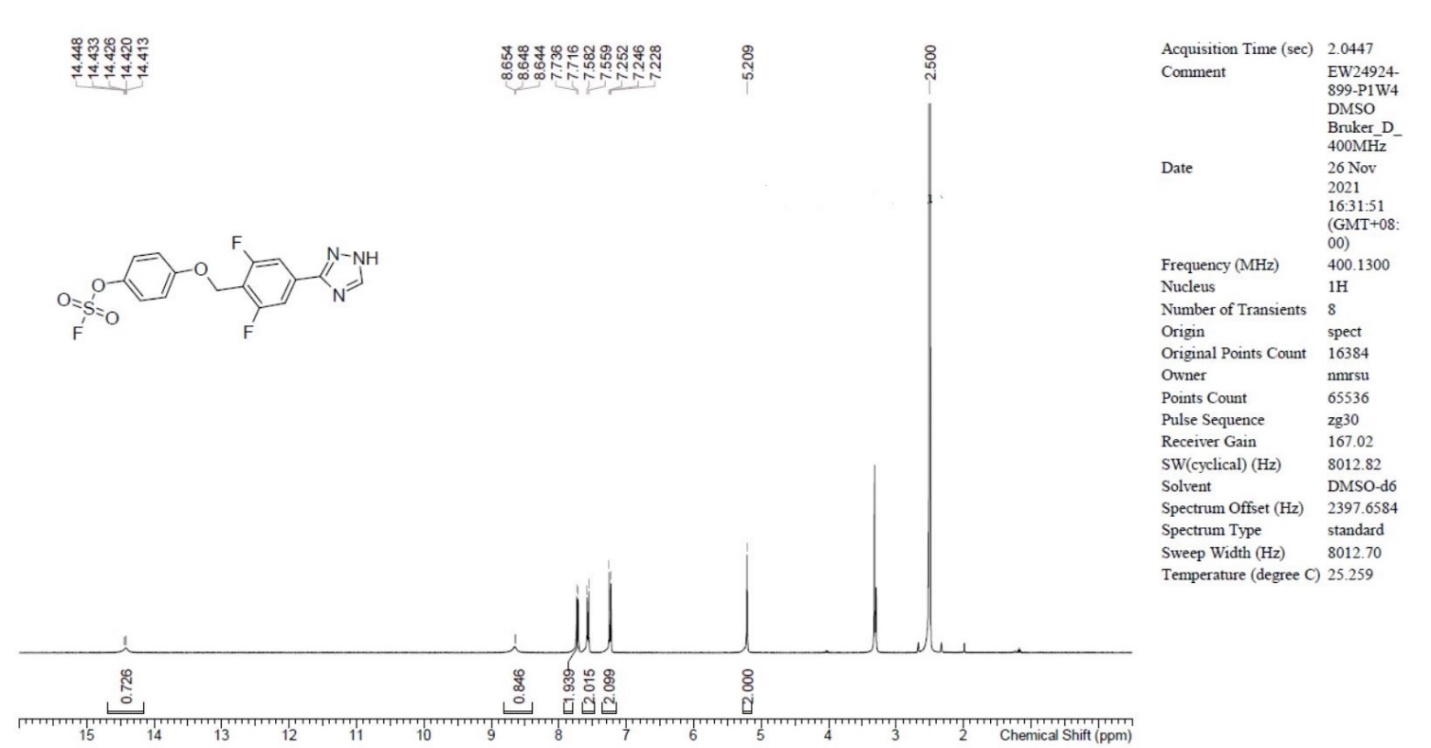


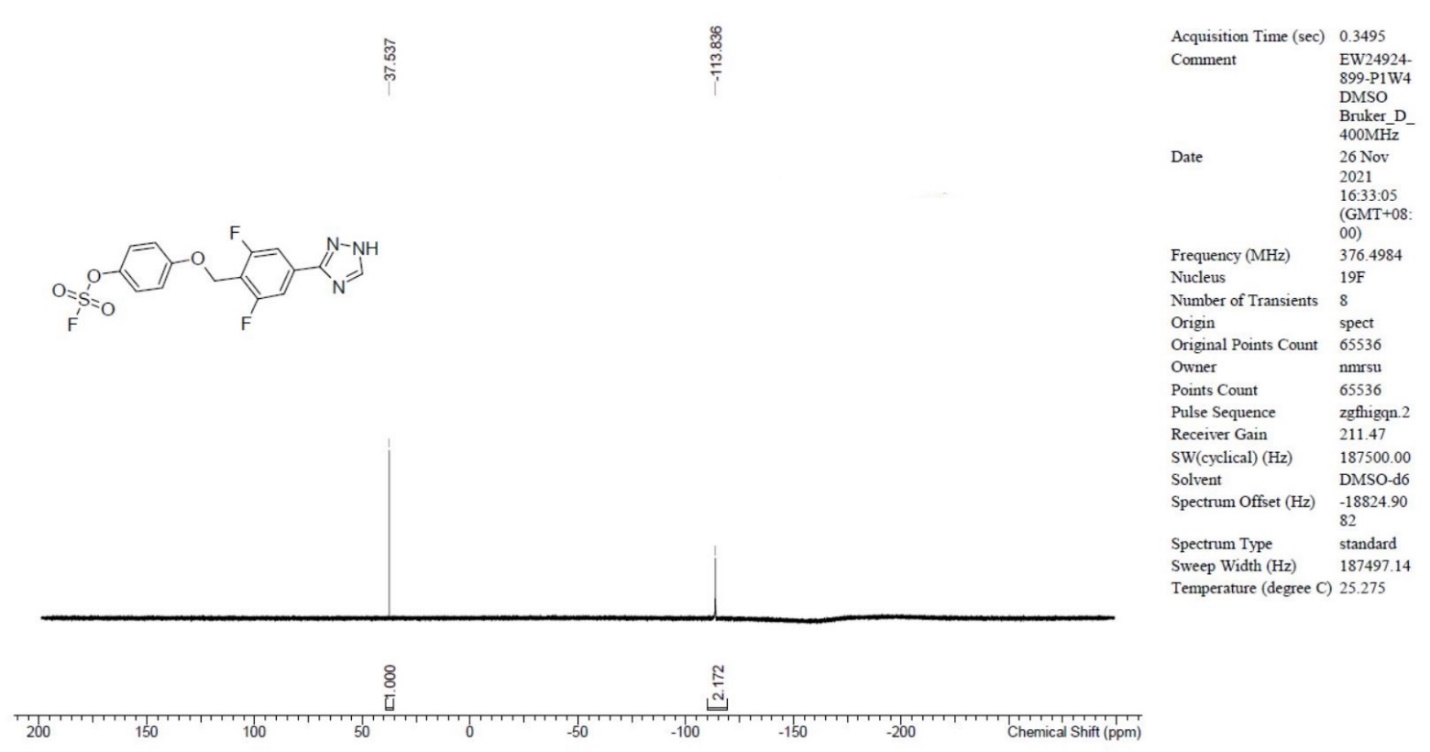


^1^H NMR of compound **19a**


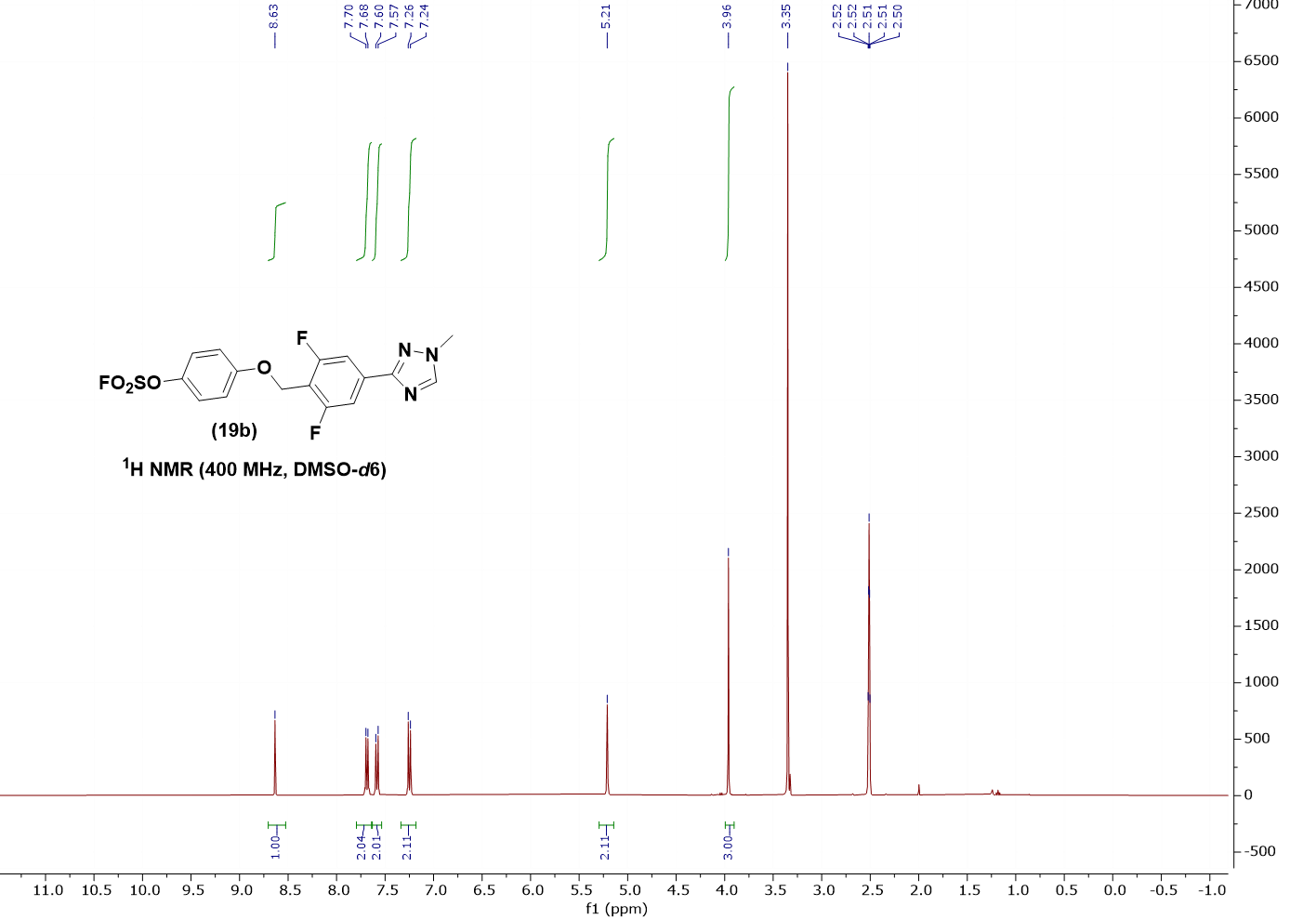


^1^H and ^19^F NMR of compound **19c**


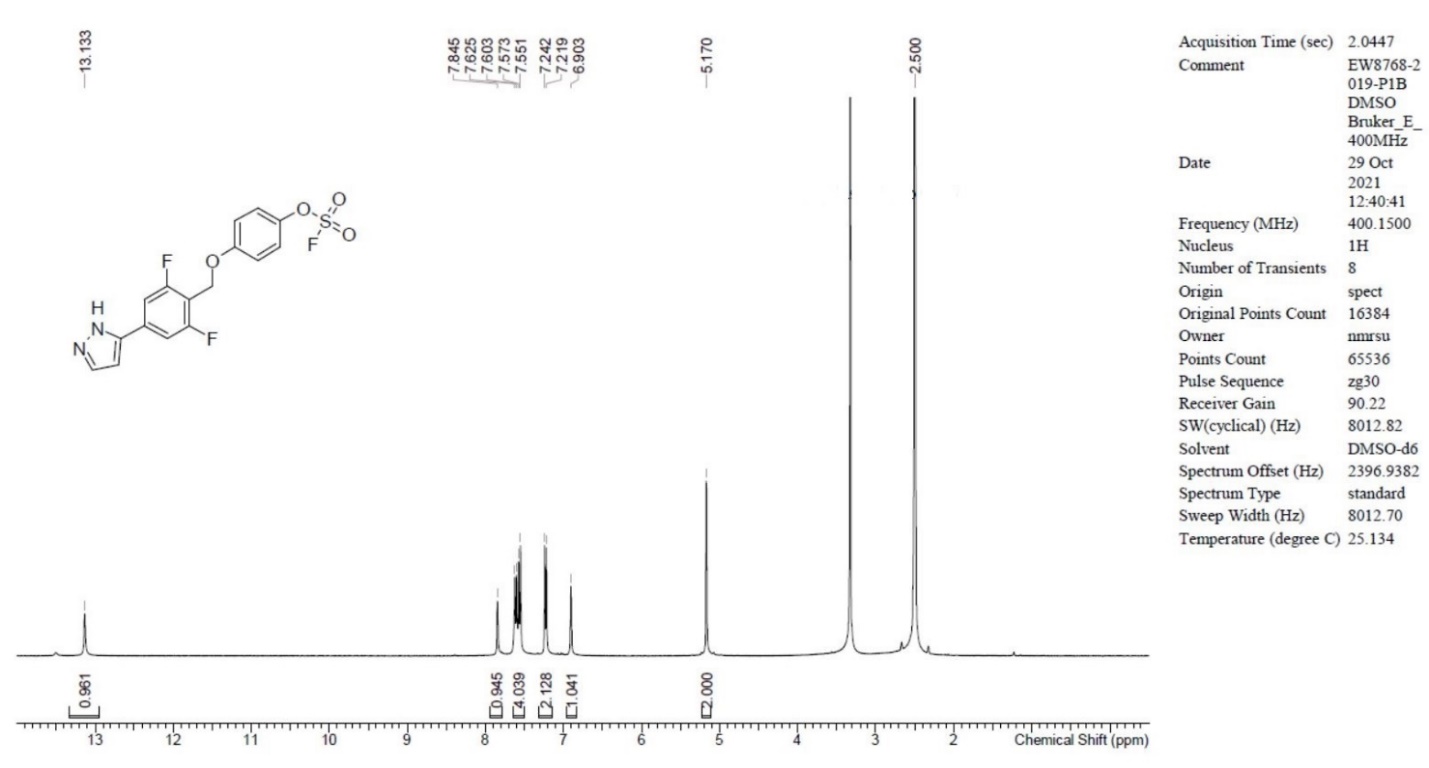


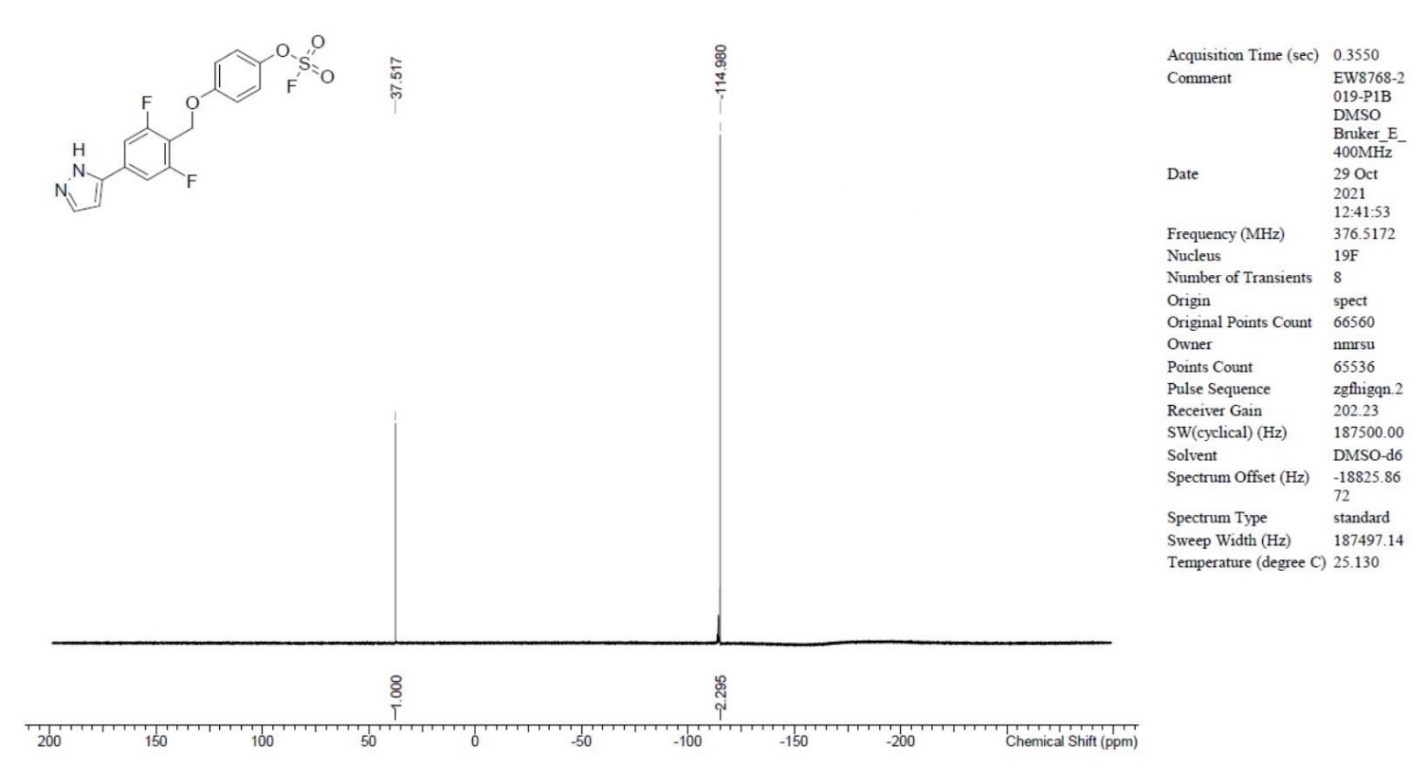


^1^H and ^19^F NMR of compound **19d**


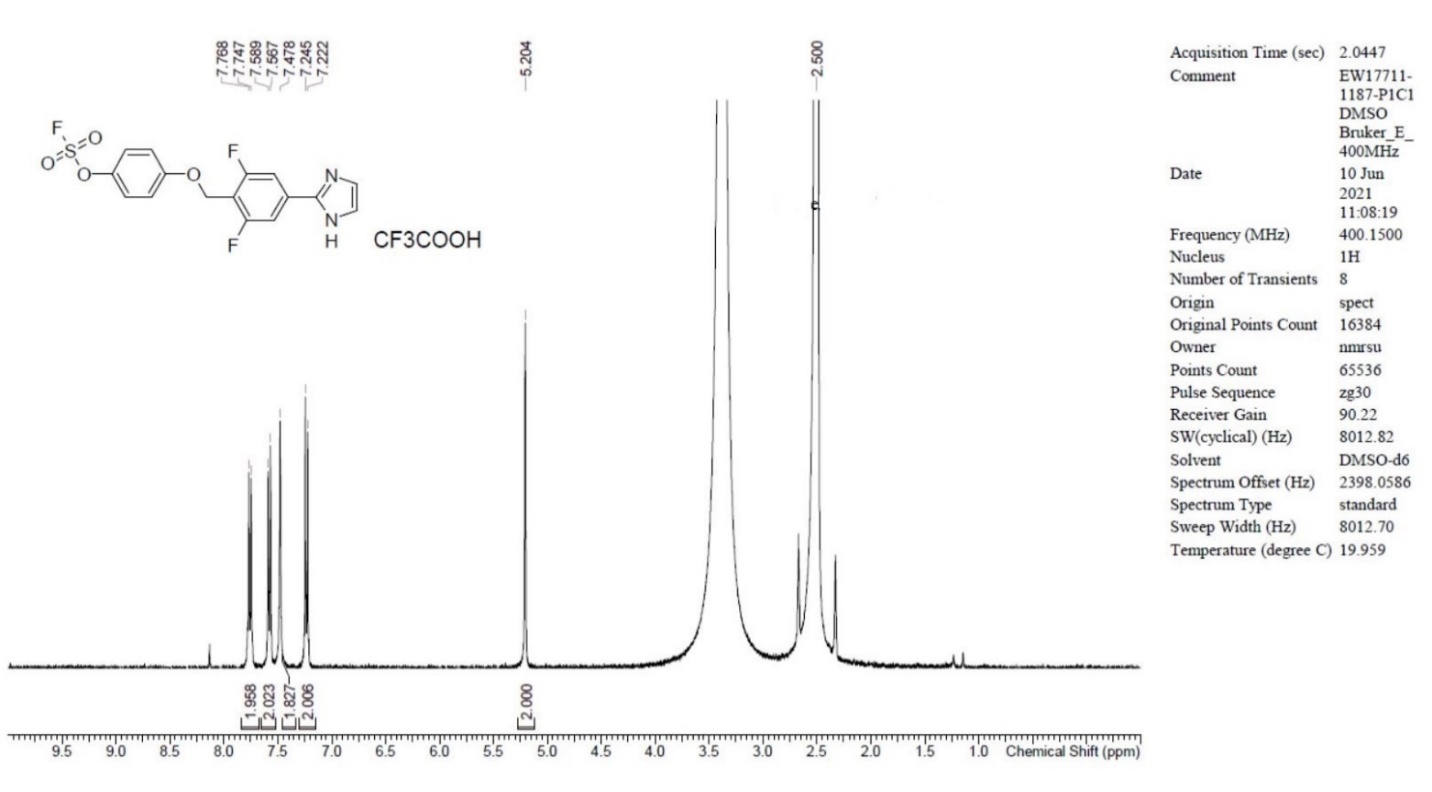


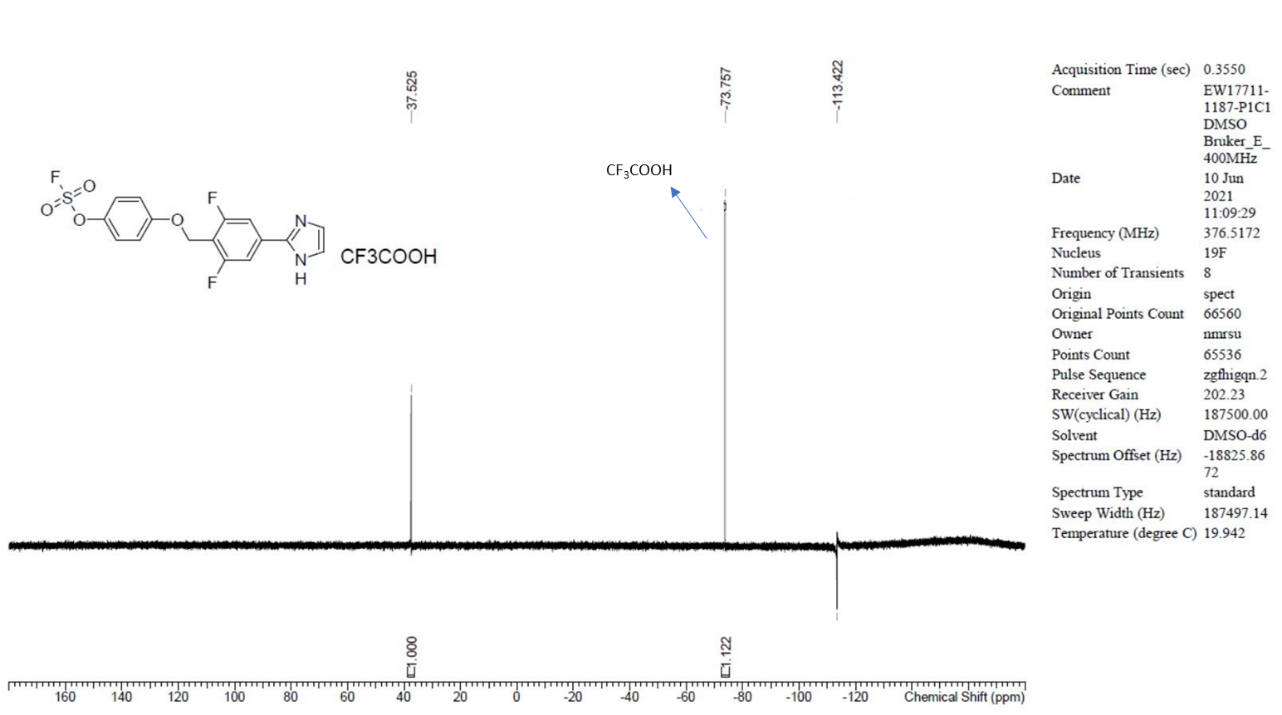


^1^H and ^19^F NMR of compound **19e**


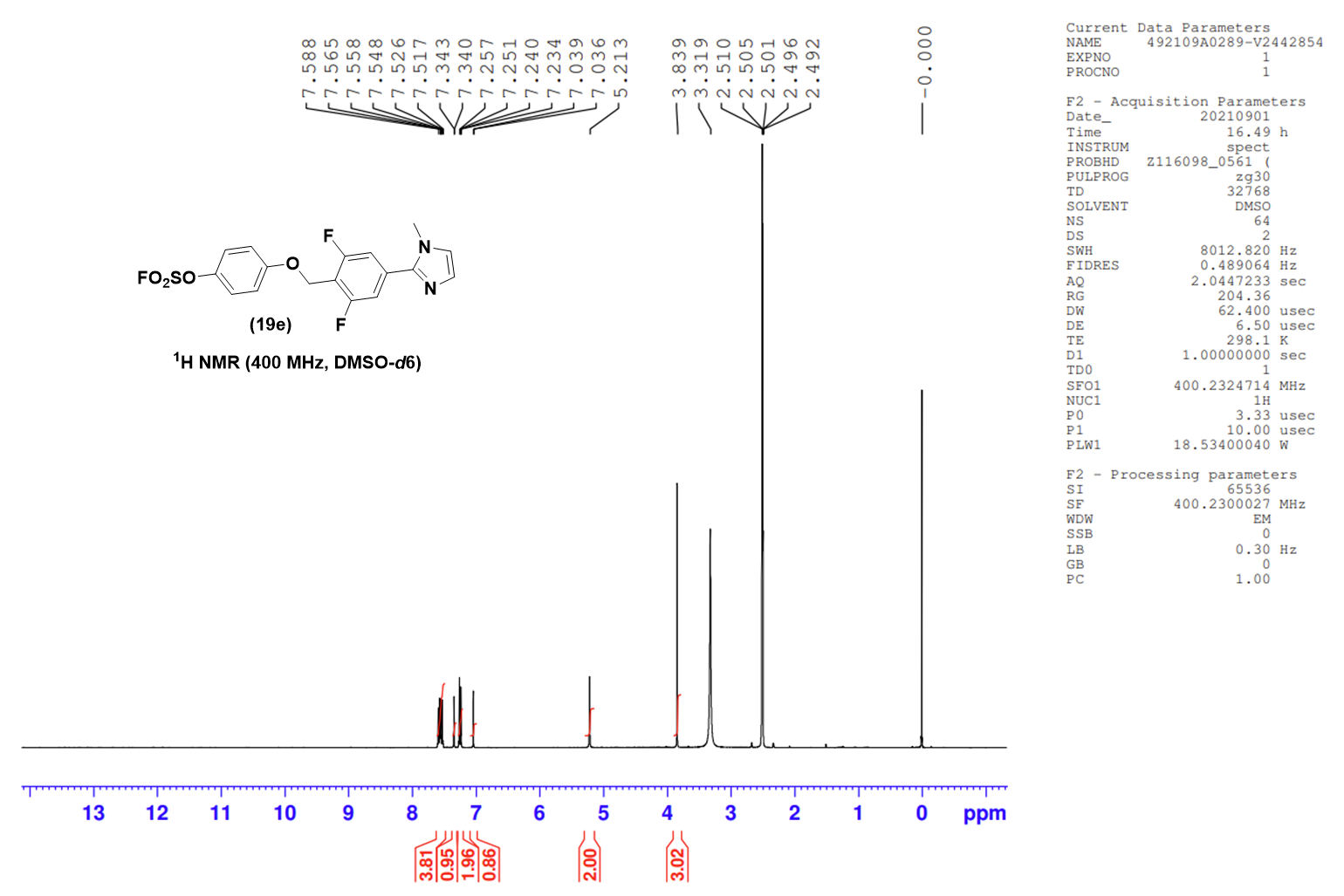


**
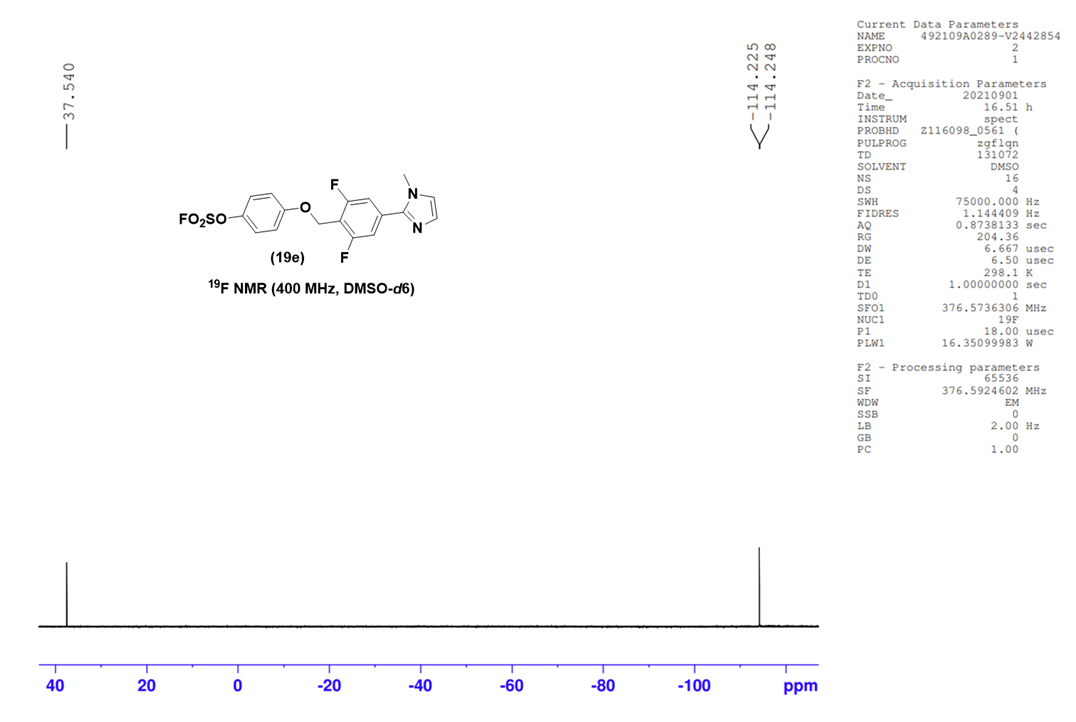
**

^1^H and ^19^F NMR of compound **19f**


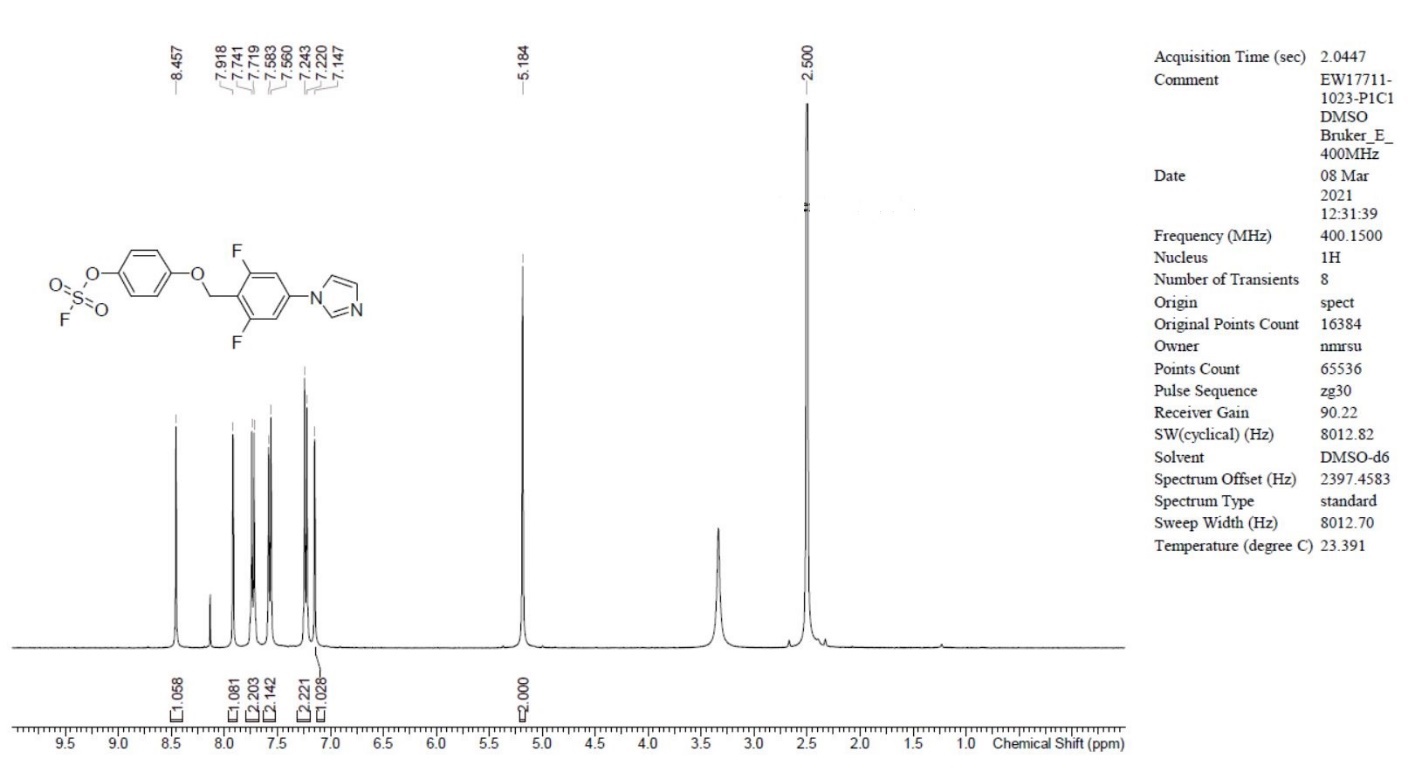


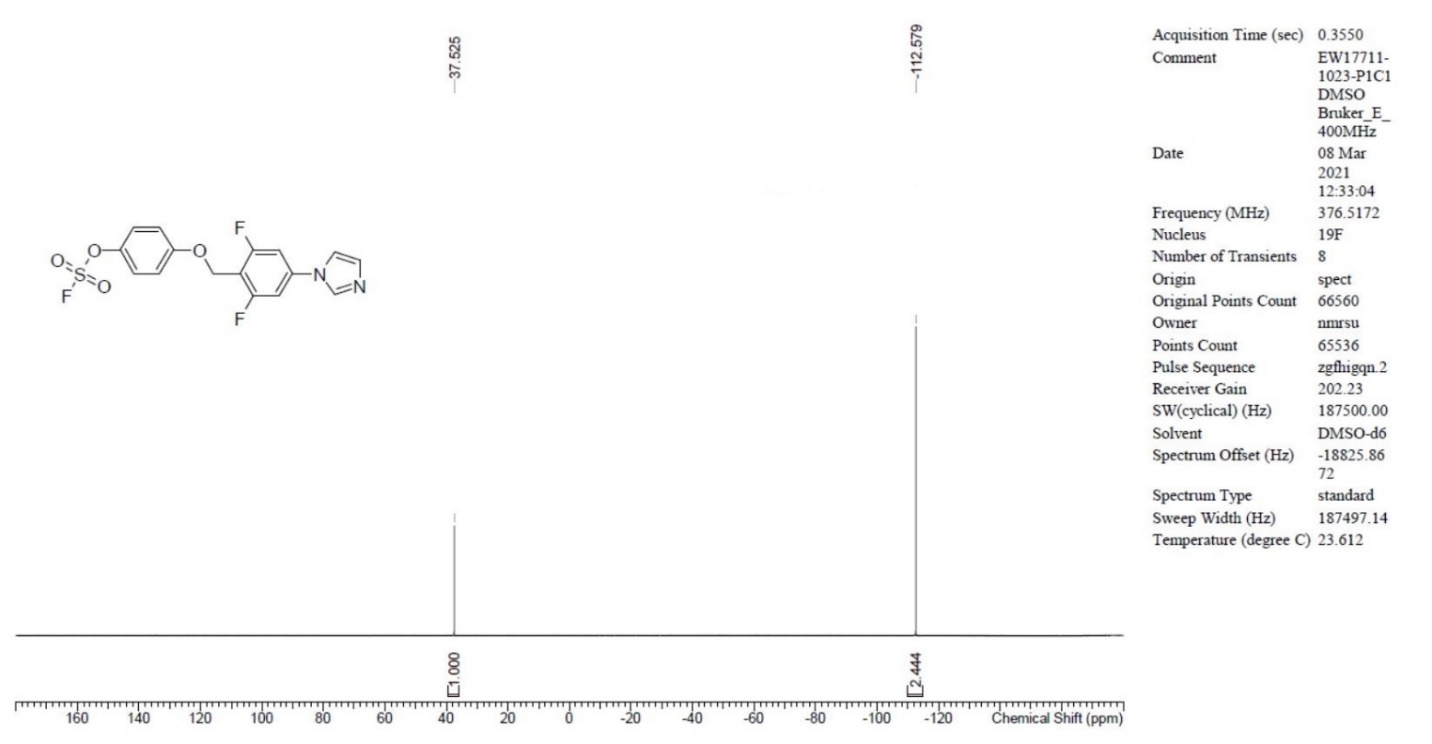


^1^H and ^19^F NMR of compound **19g**


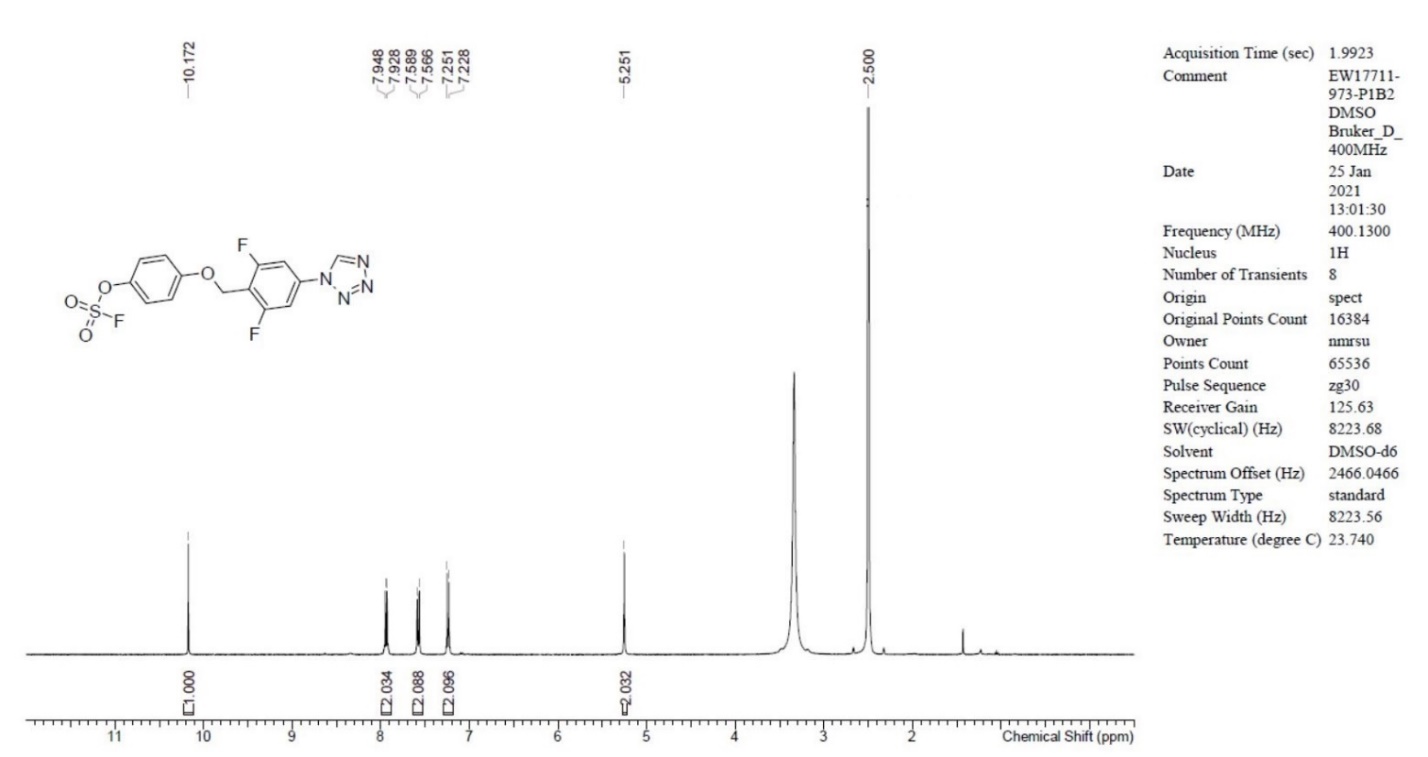


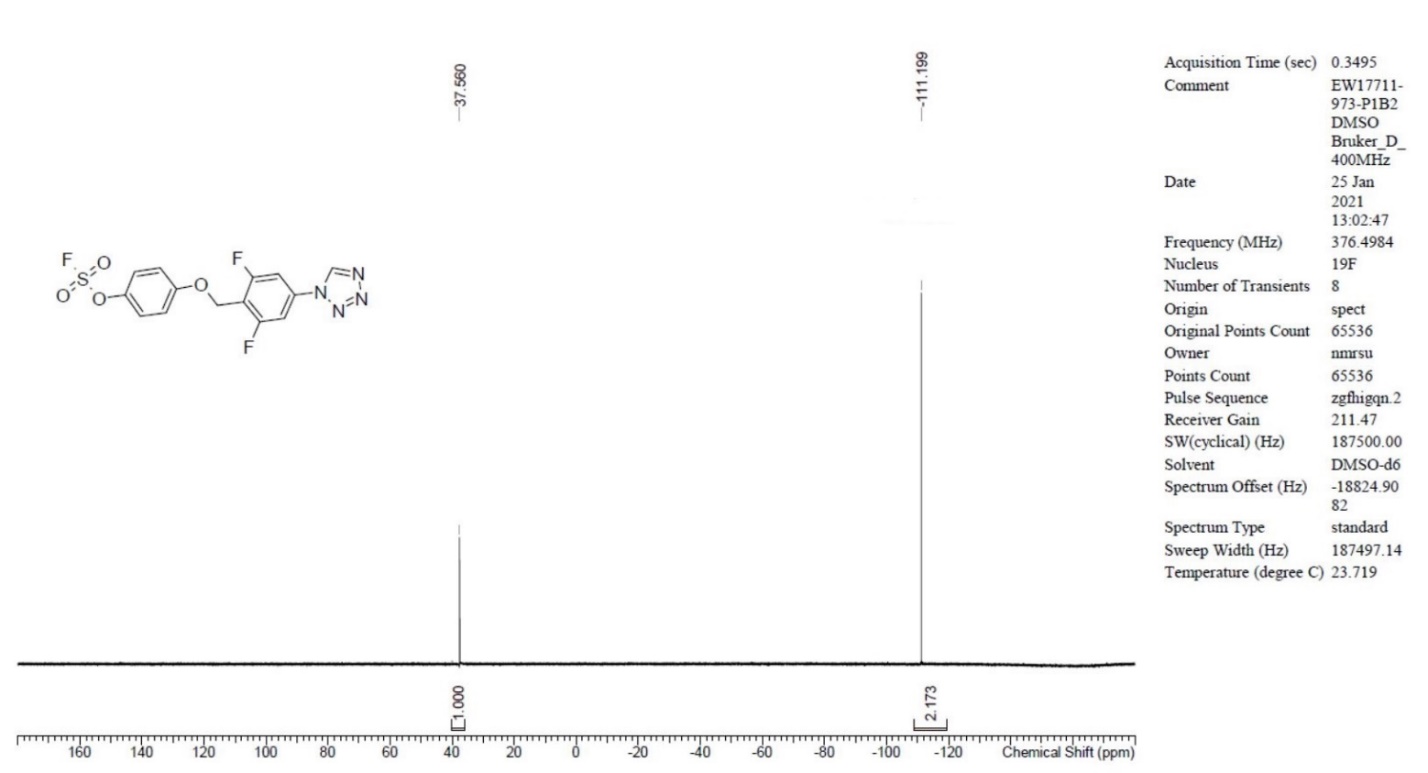


^1^H and ^19^F NMR of compound **19h**


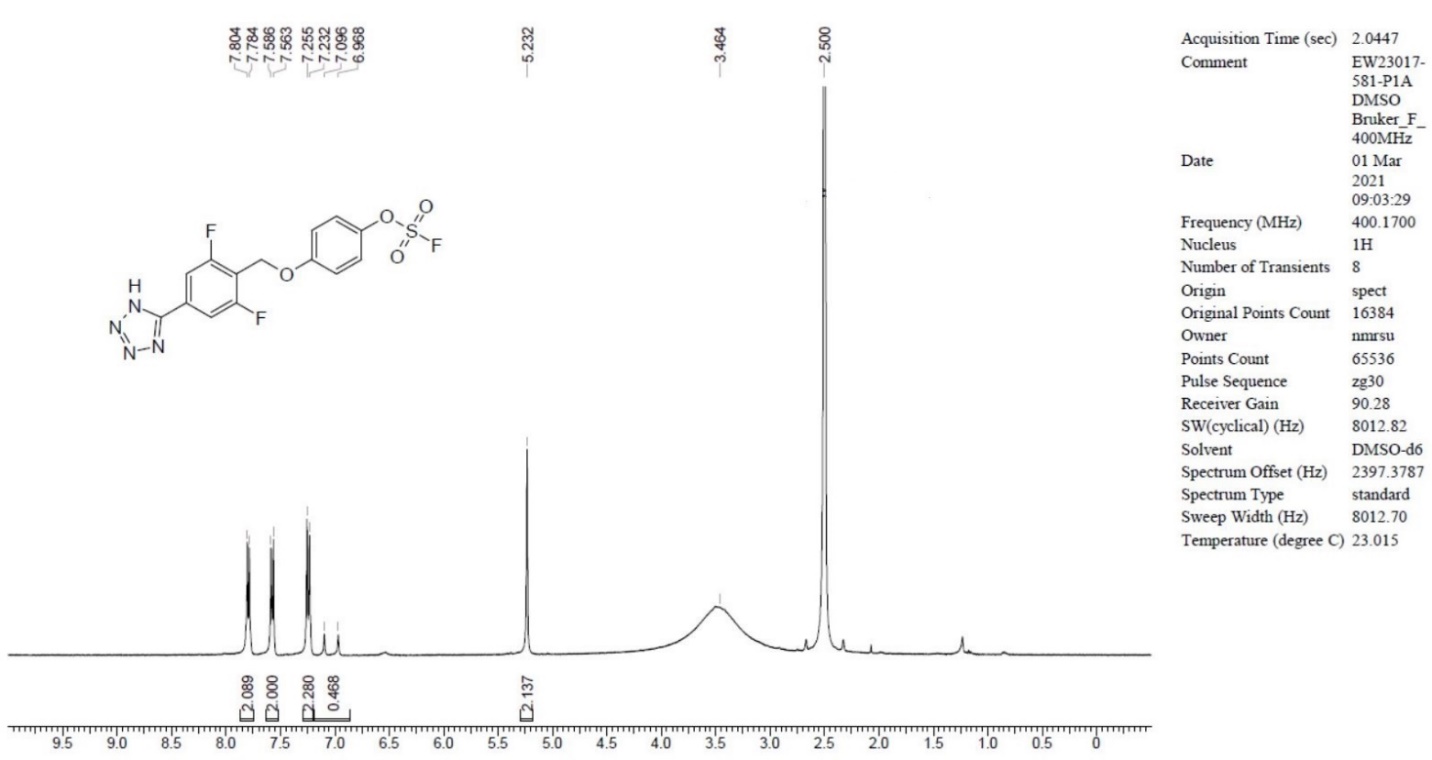


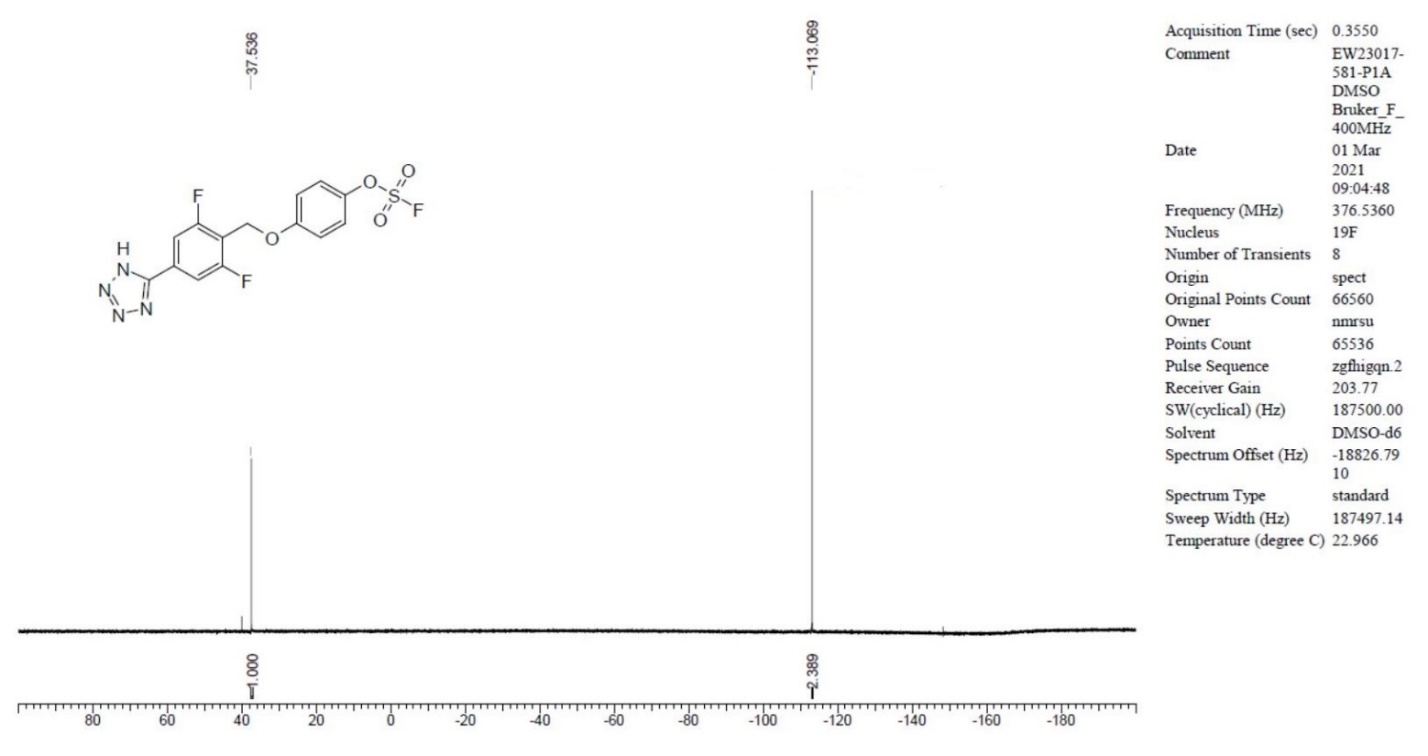


^1^H and ^19^F NMR of compound **19i**


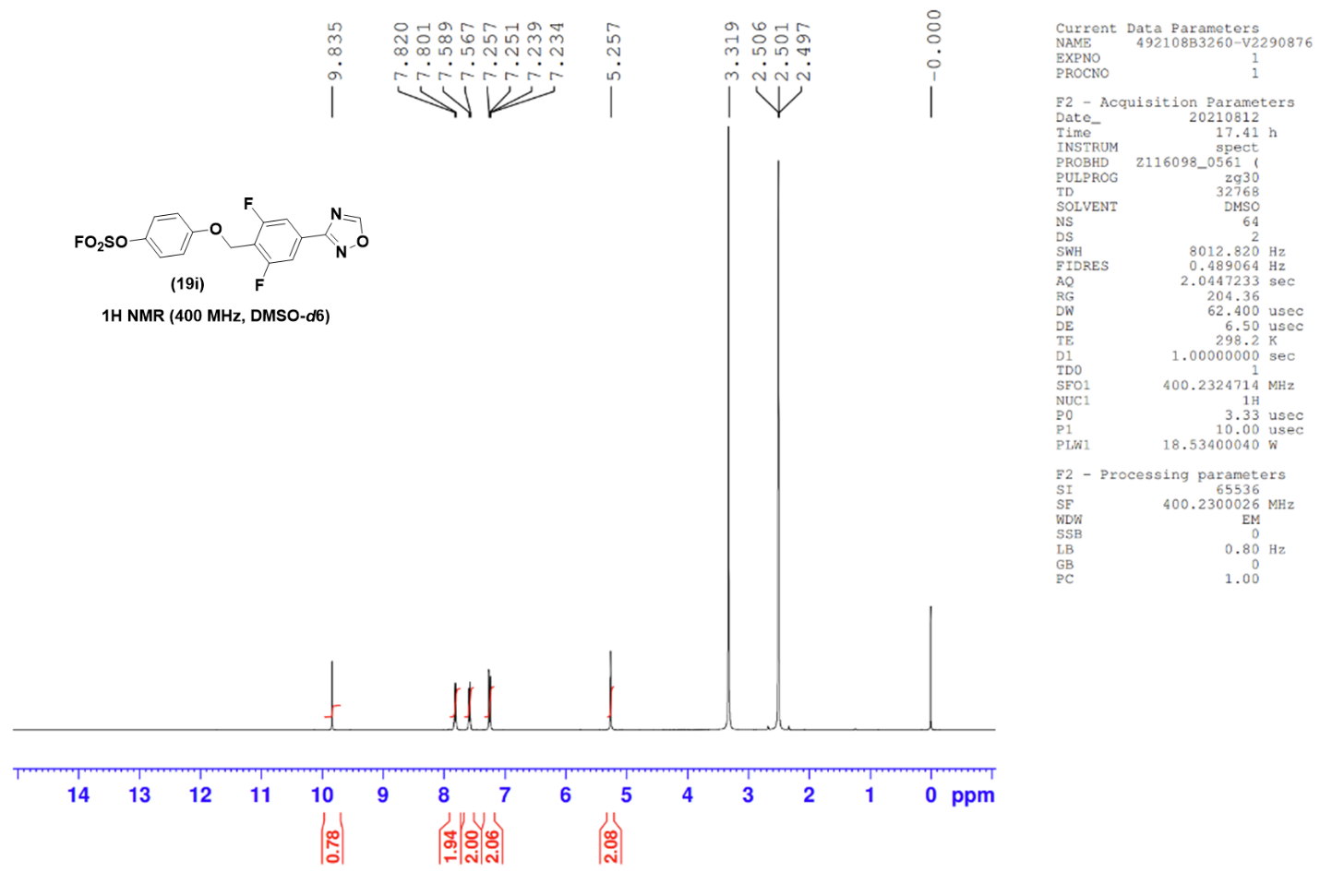


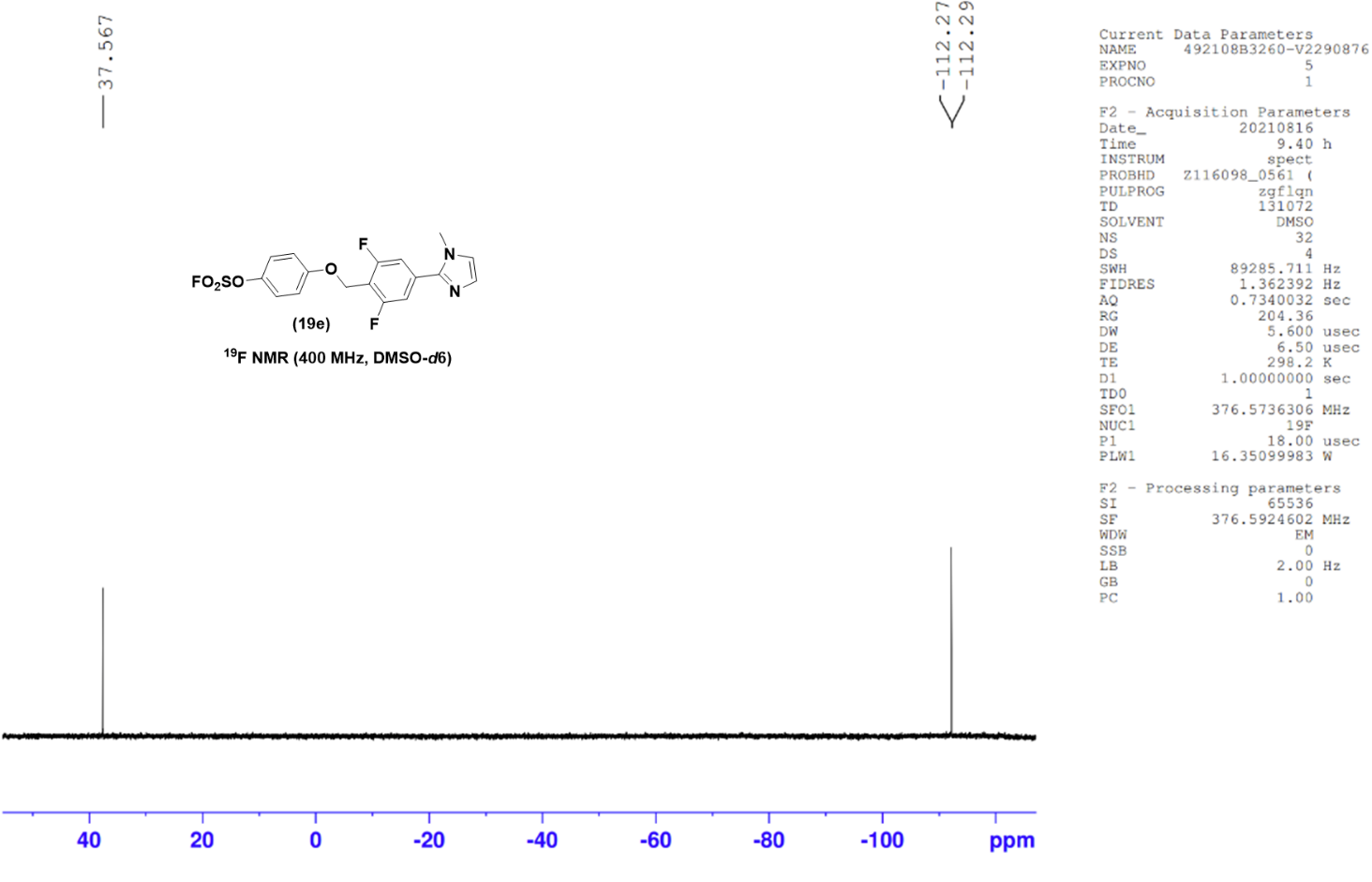


^1^H and ^19^F NMR of compound **19j**

**
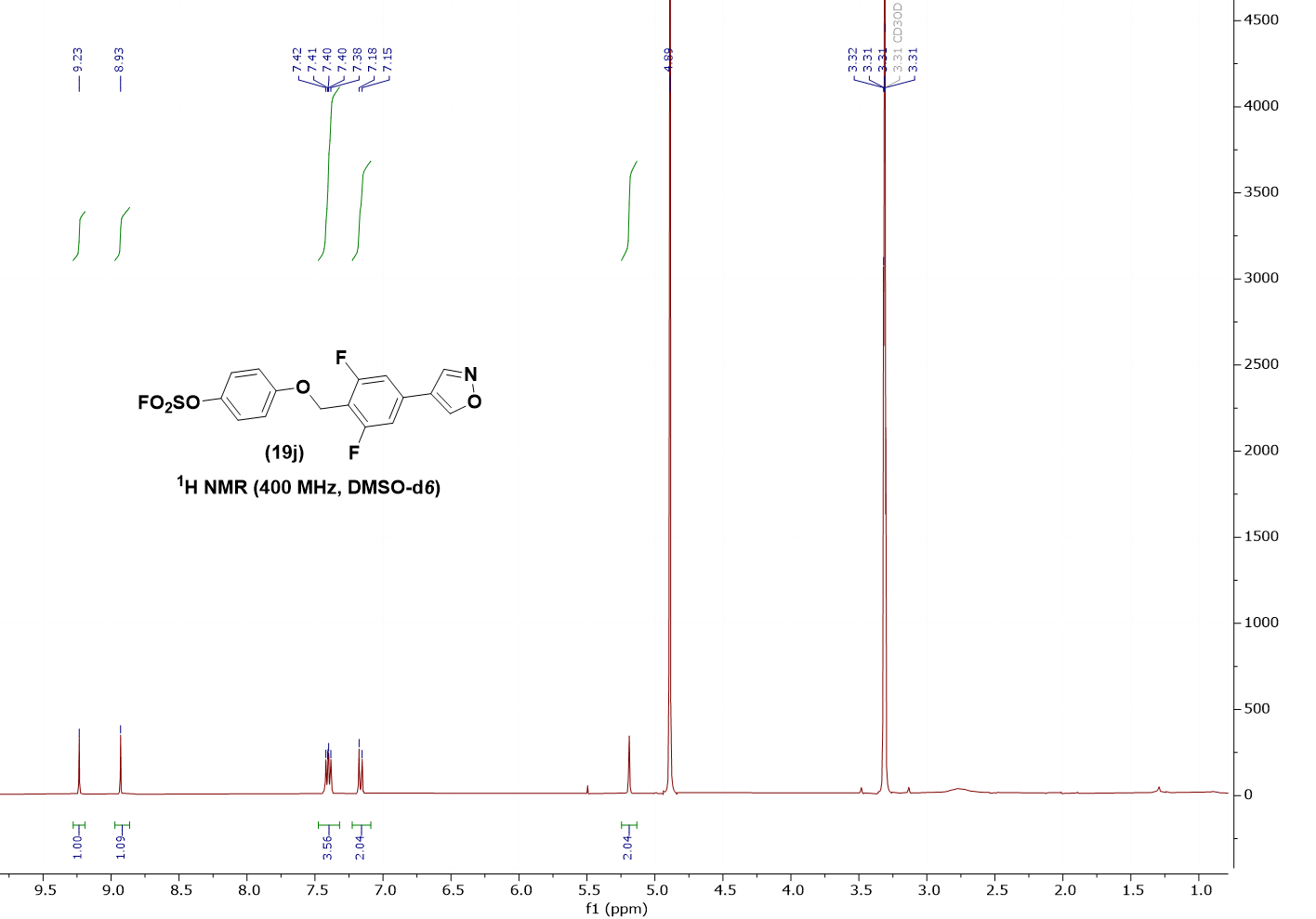
**

**
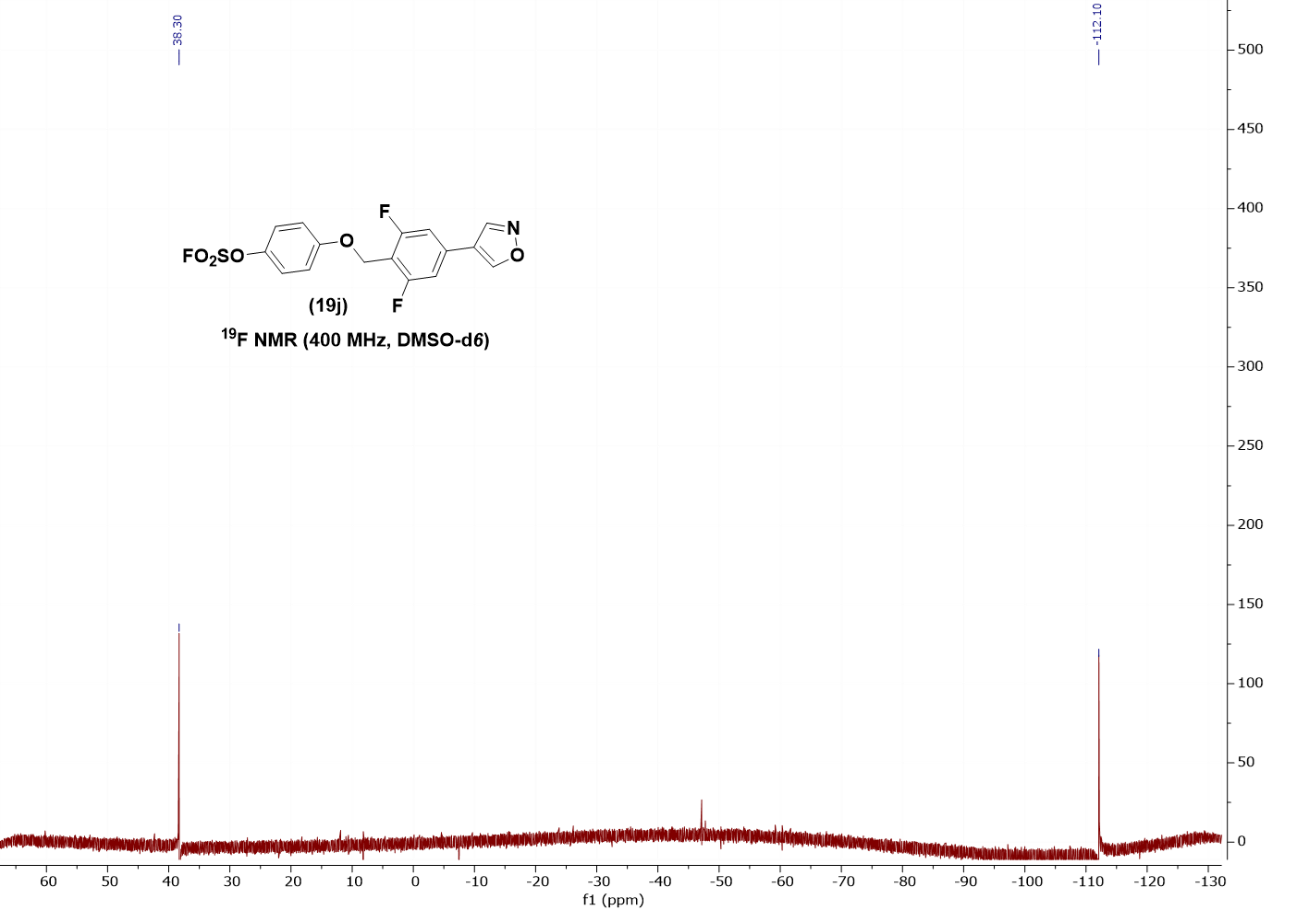
**

^1^H and ^19^F NMR of compound **20a**


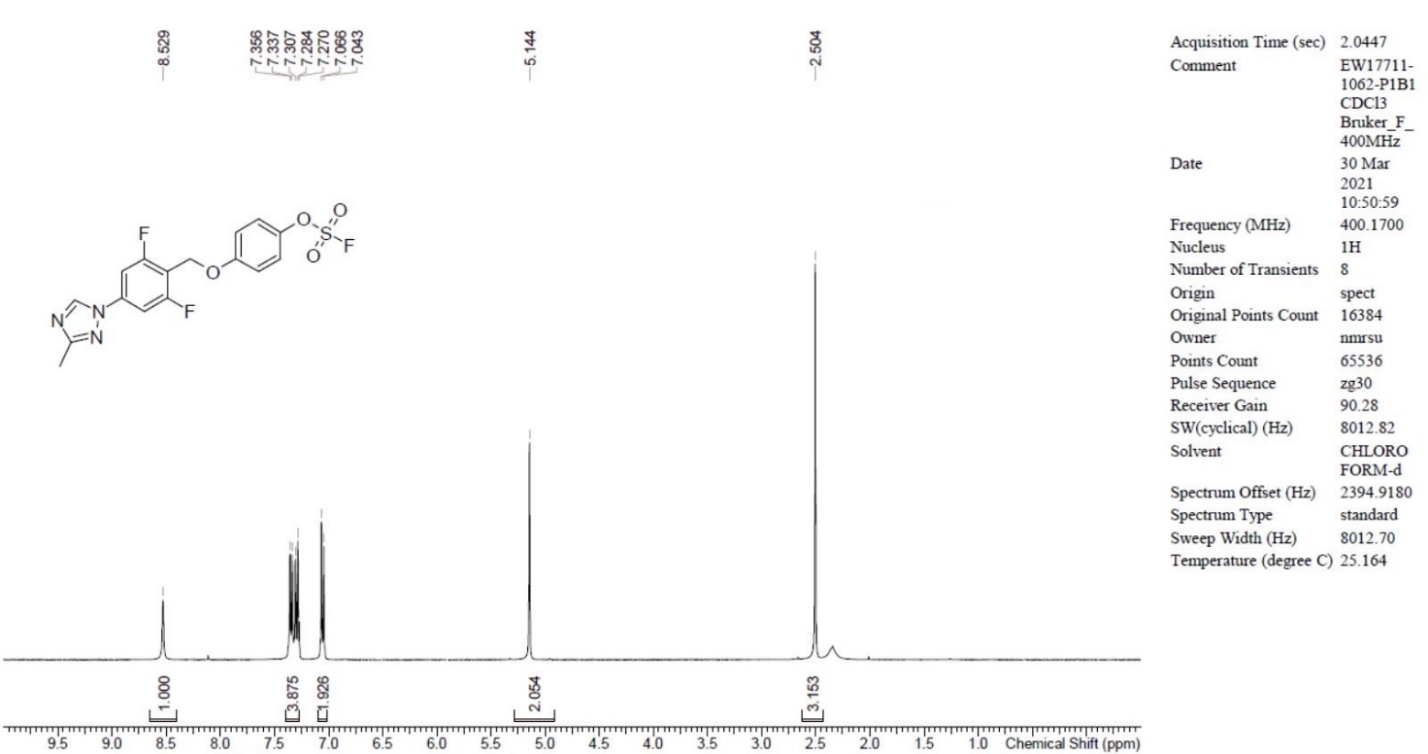


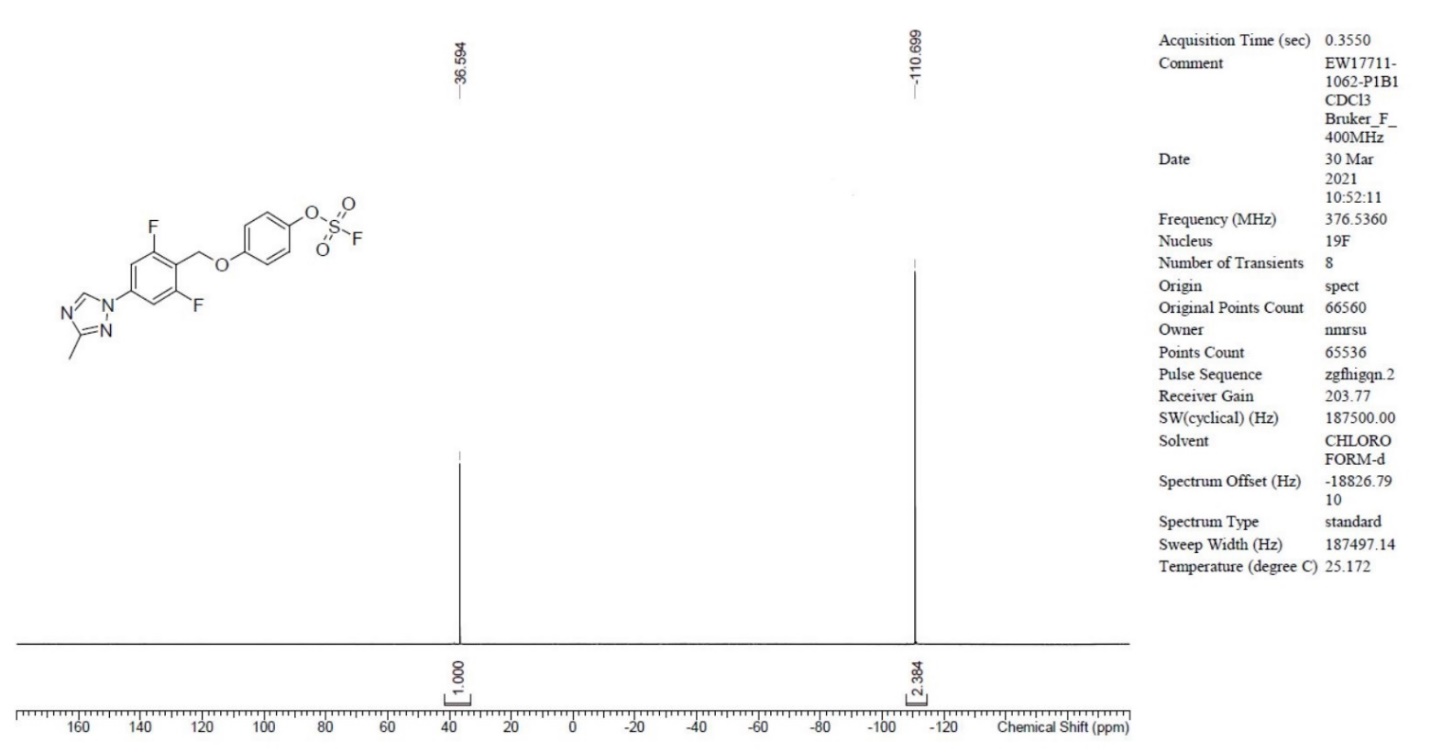


^1^H NMR of compound **20b**


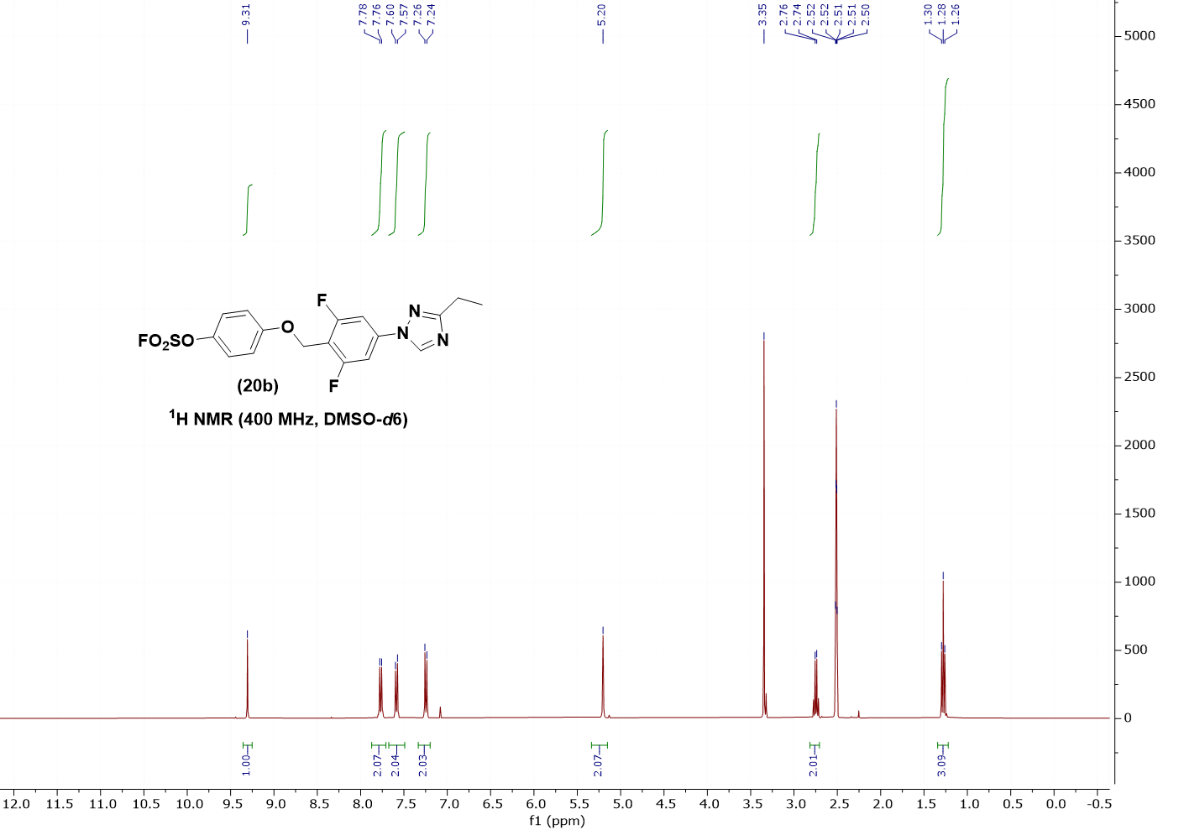


^1^H NMR of compound **20c**

**
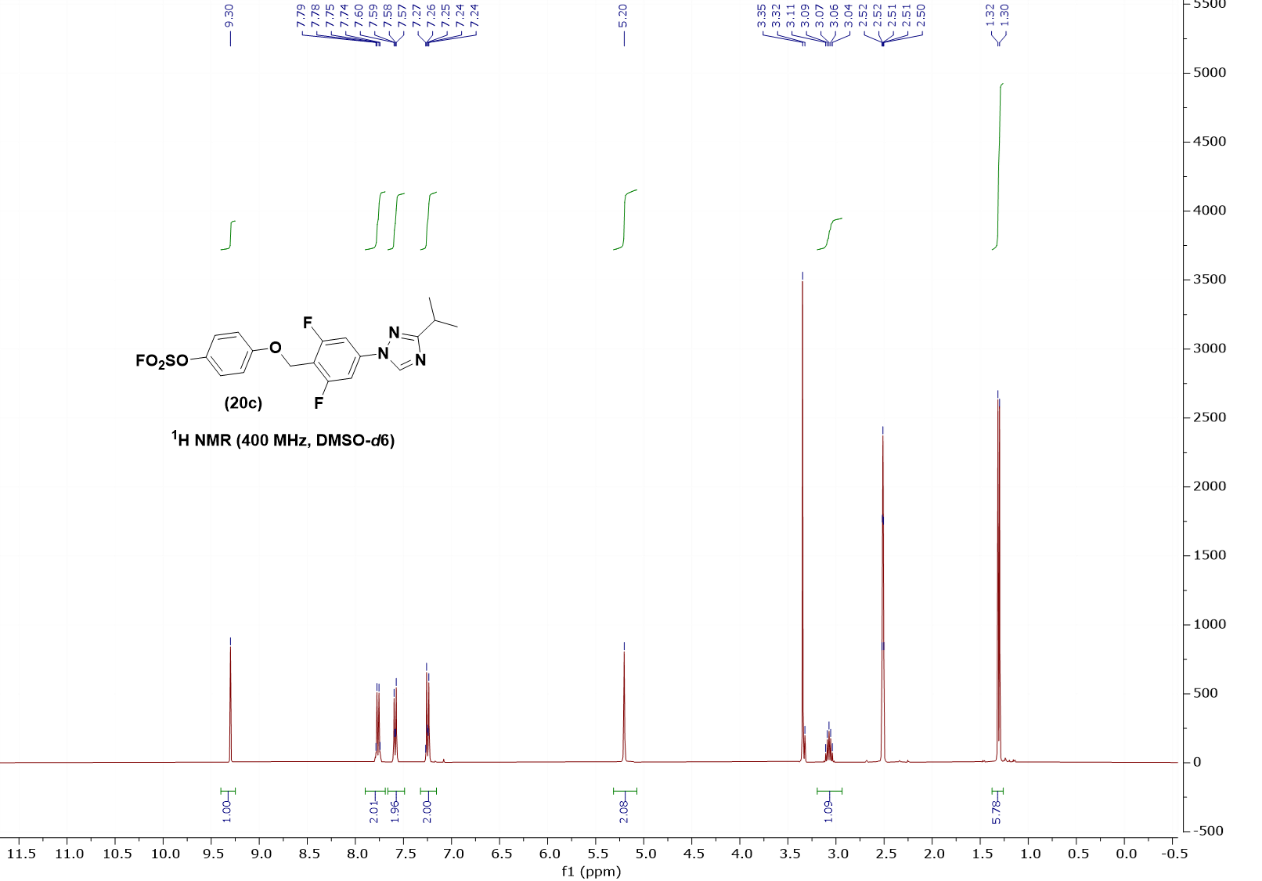
**

^1^H NMR of compound **20d**

**
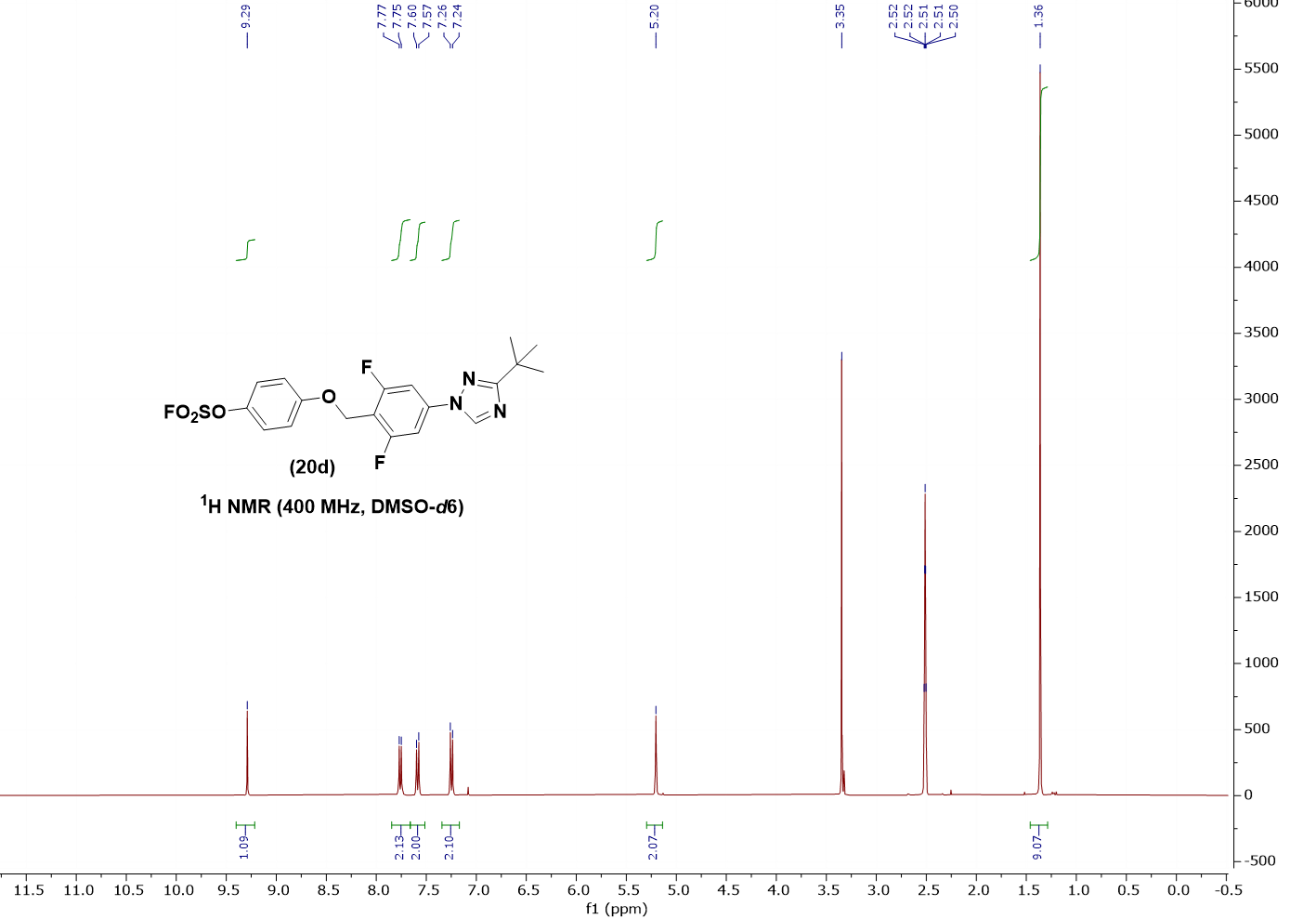
**

^1^H and ^19^F NMR of compound **20e**


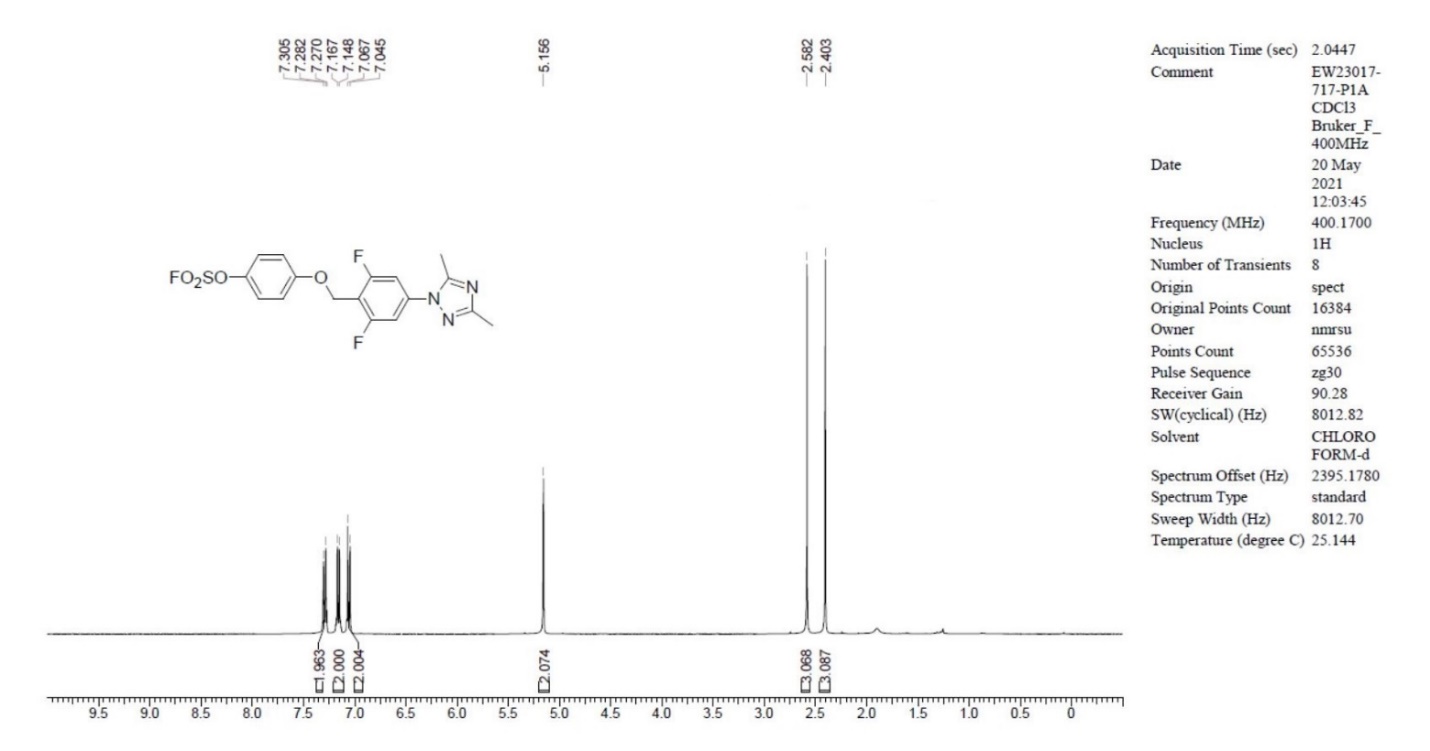


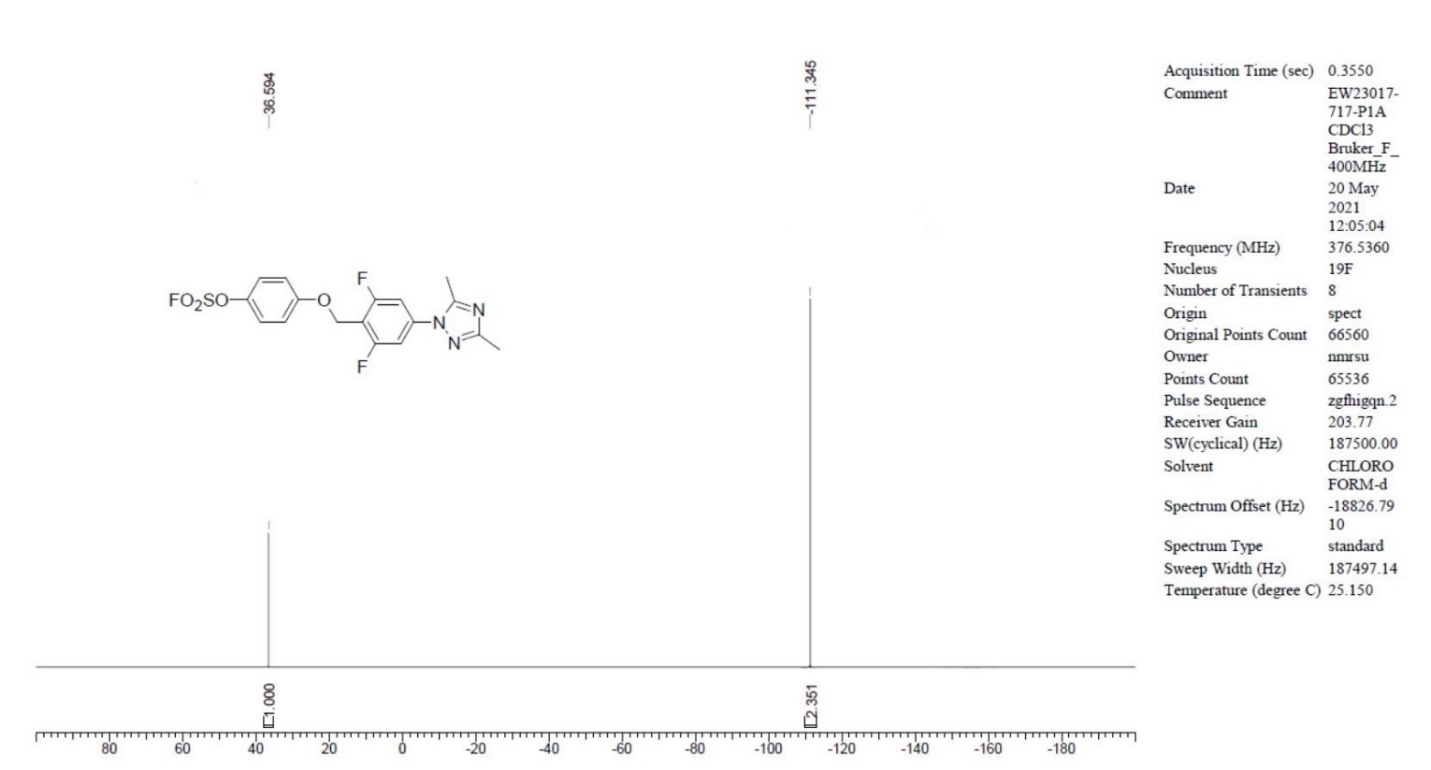


^1^H and ^19^F NMR of compound **20f**


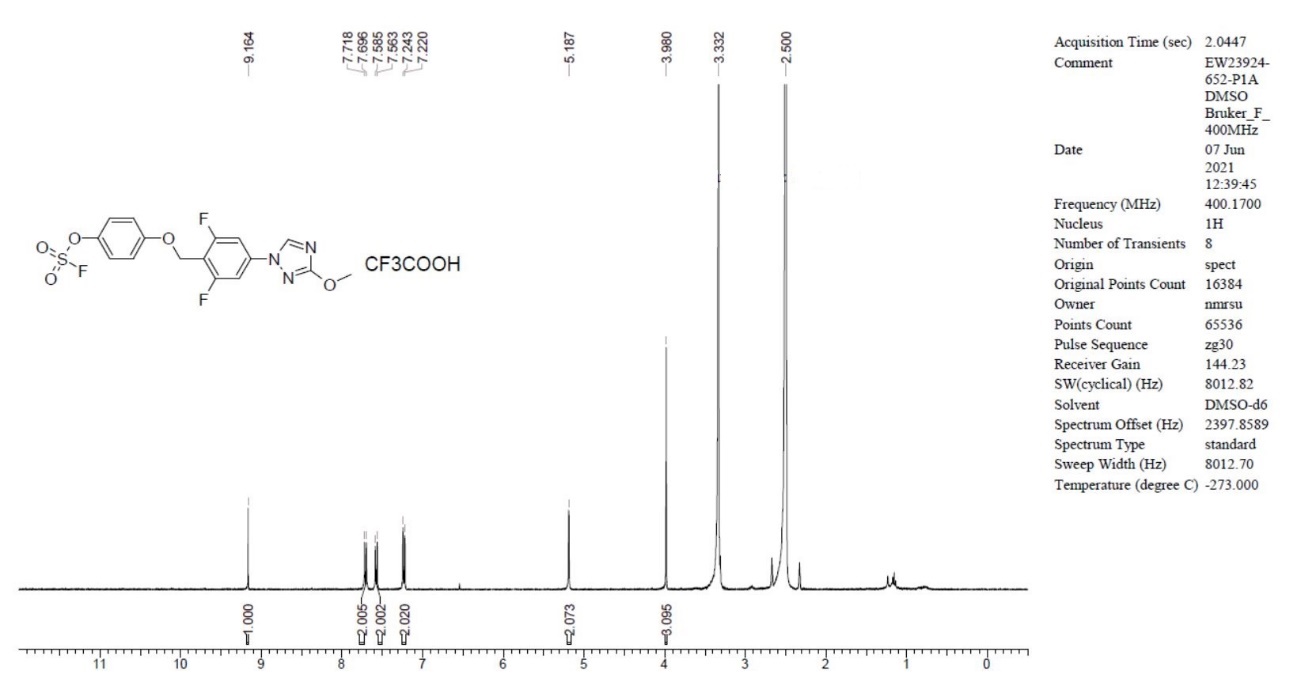


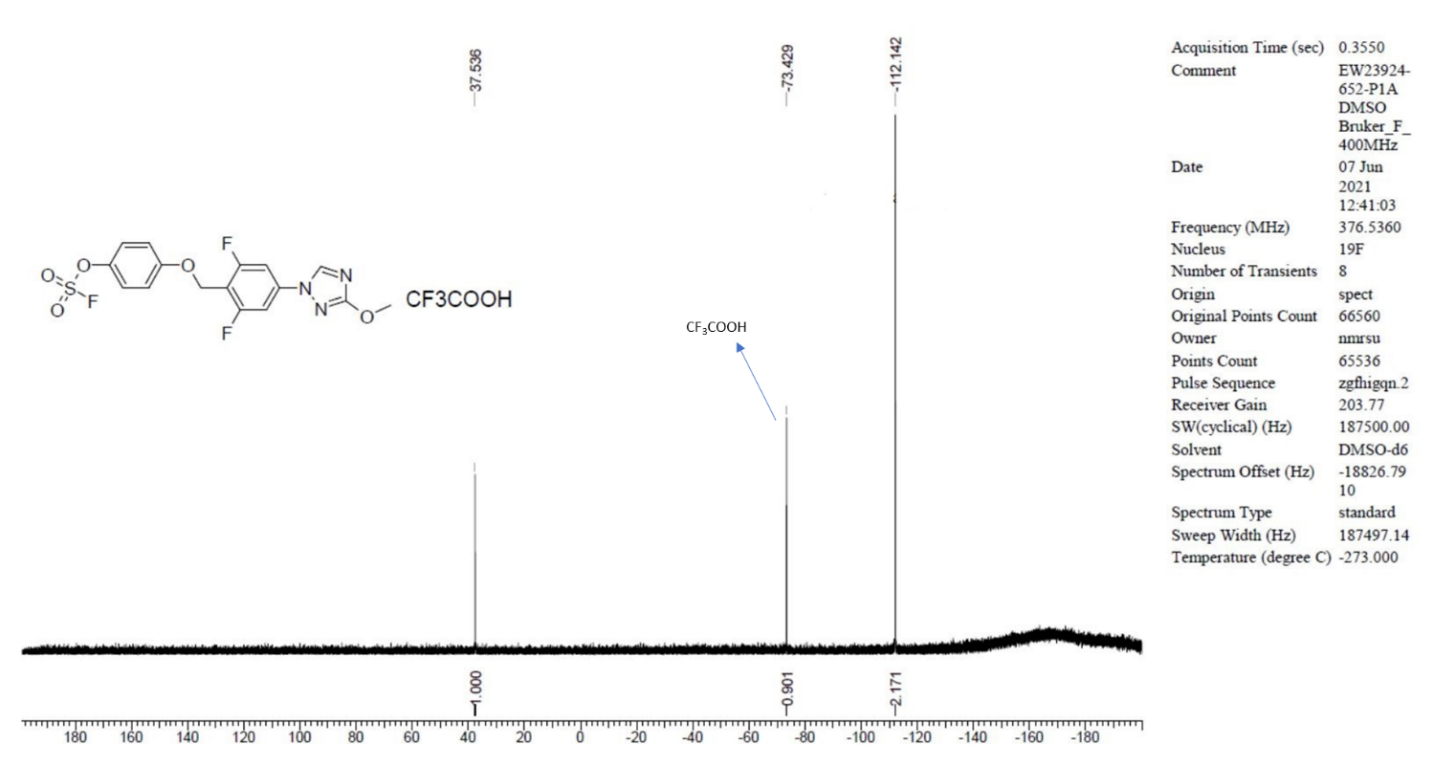


^1^H and ^19^F NMR of compound **20g**


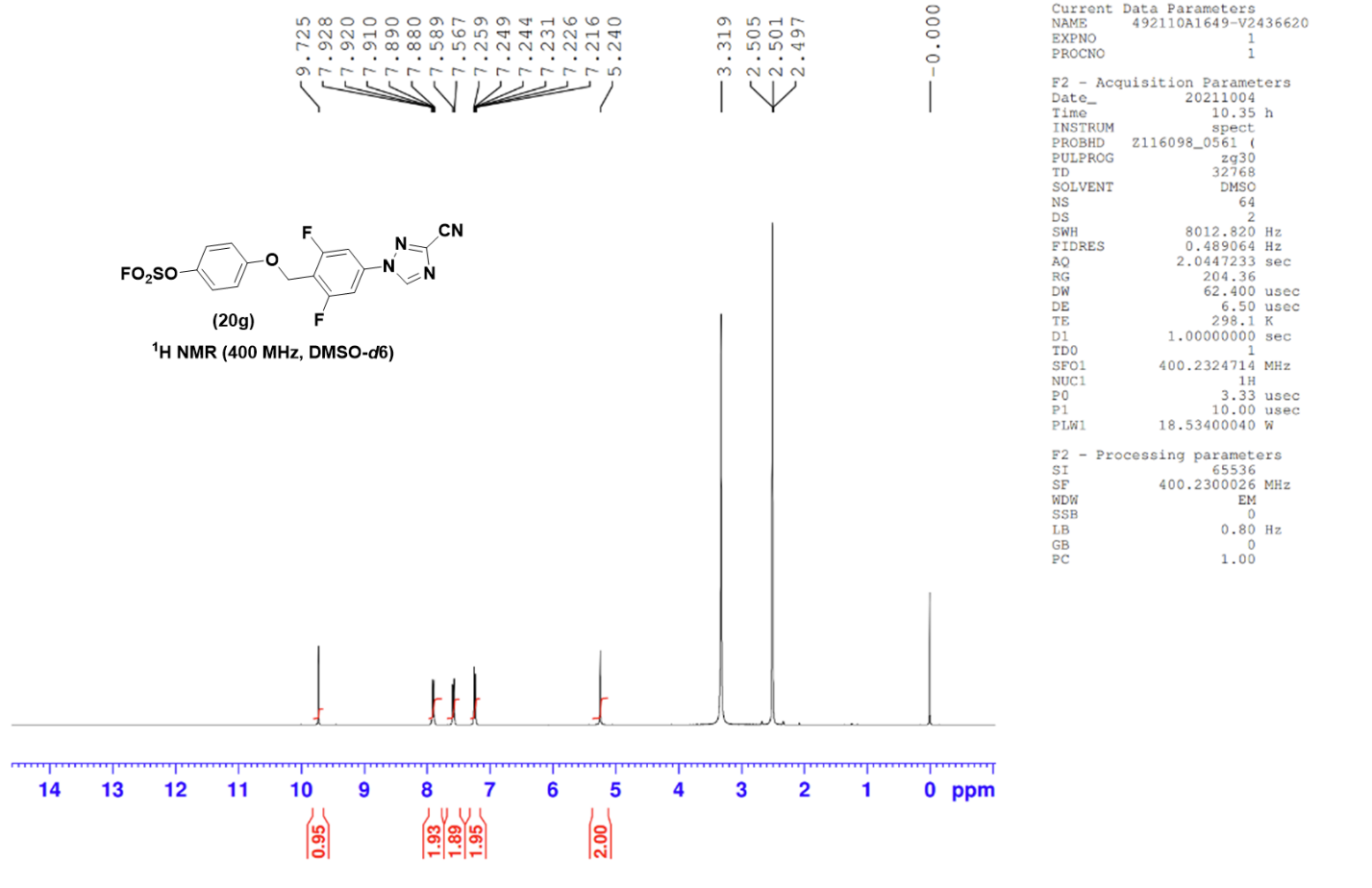


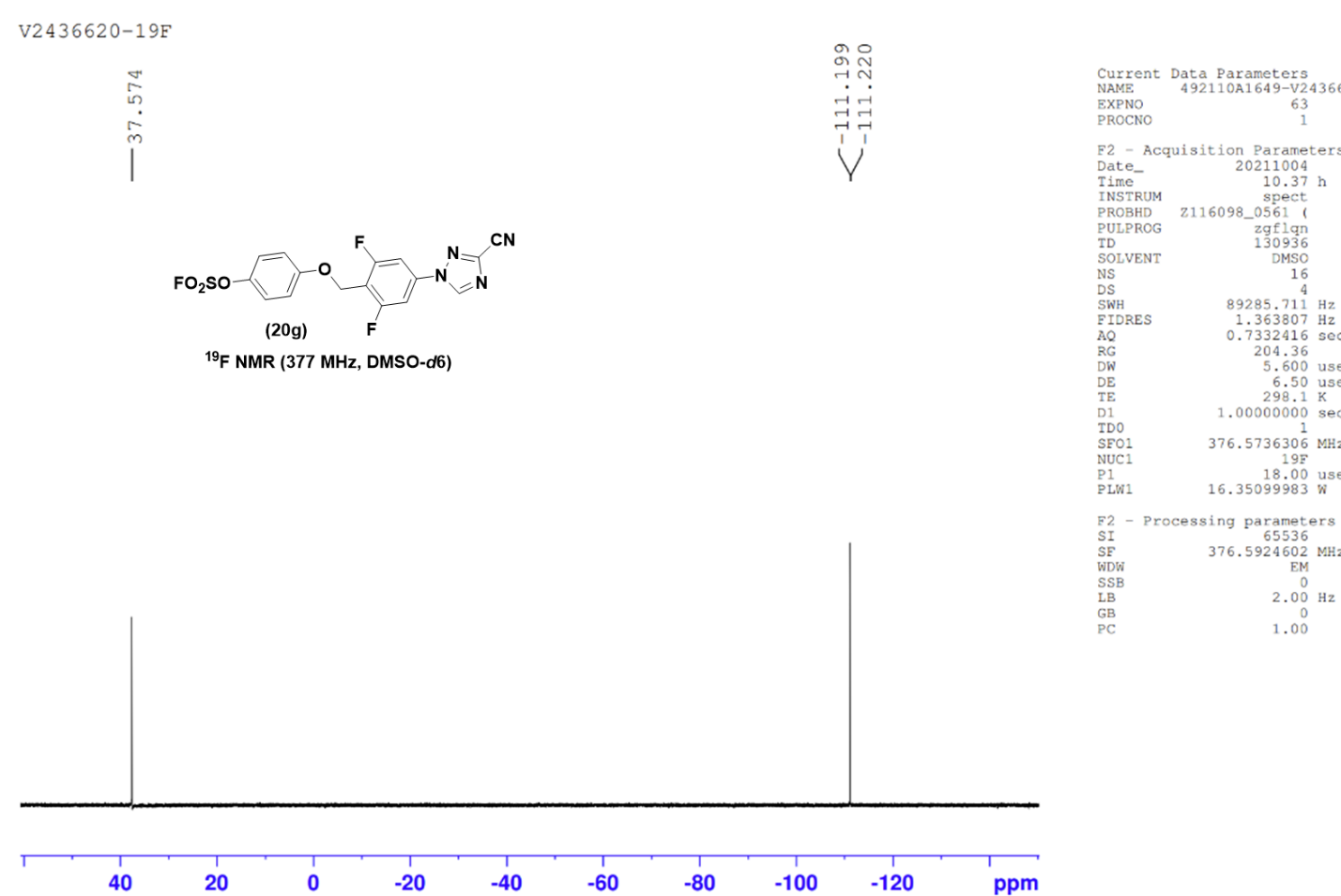


^1^H and ^19^F NMR of compound **20h**


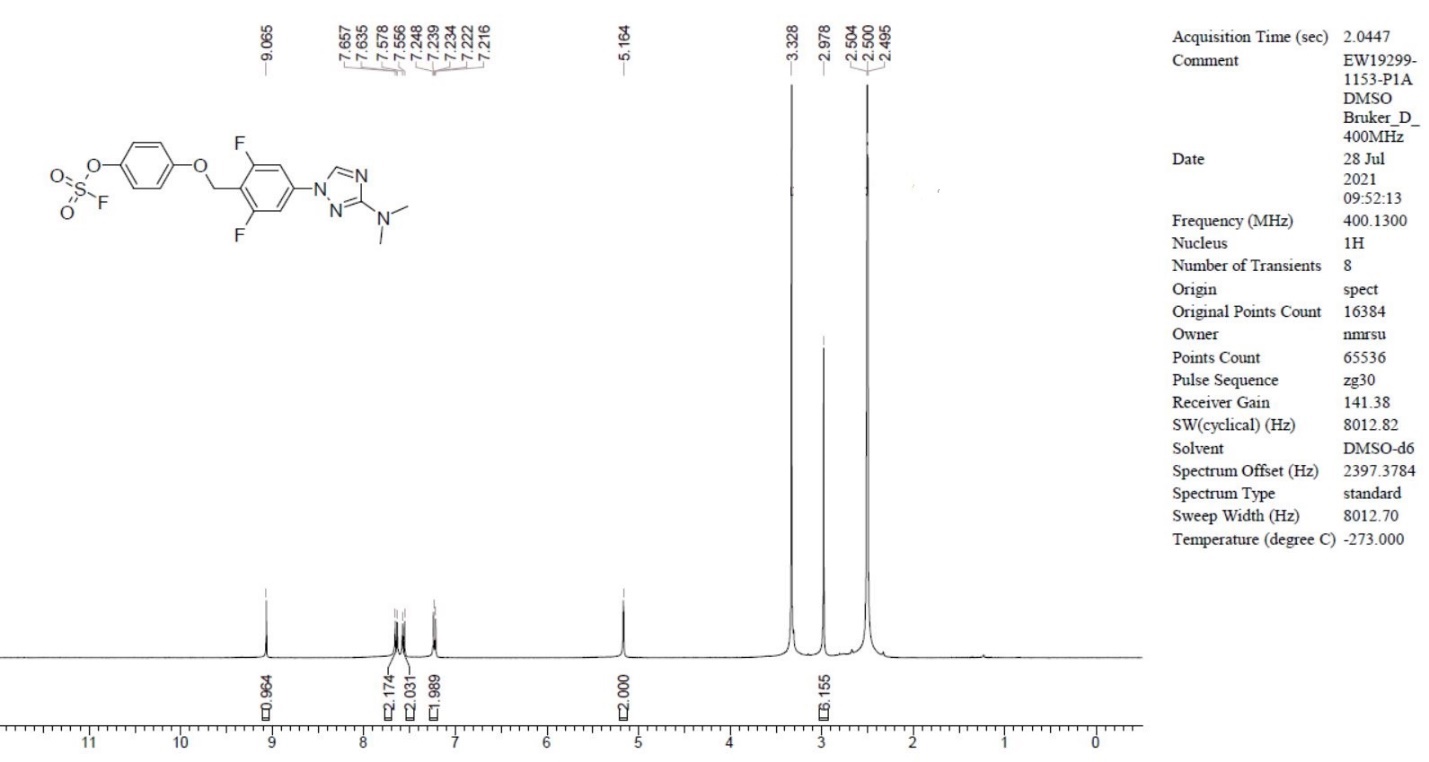


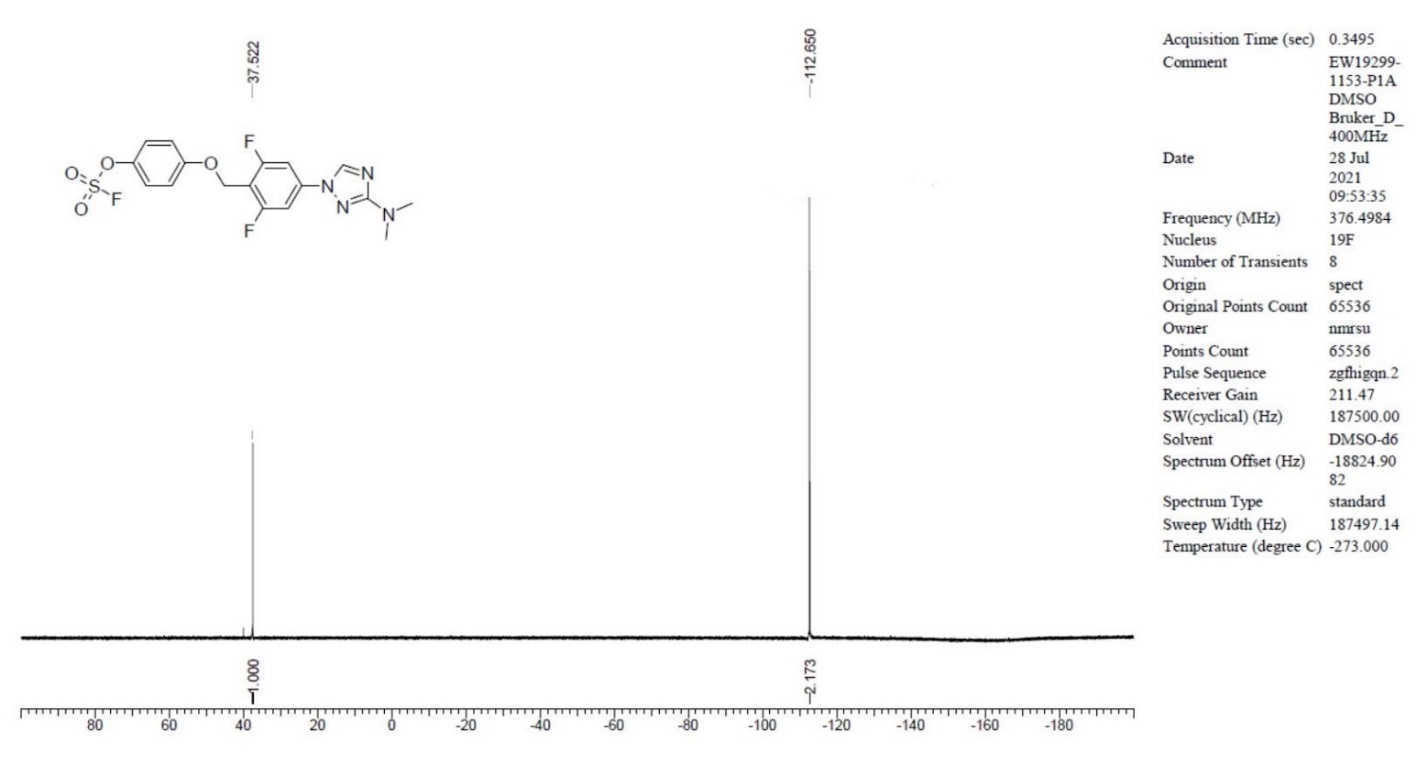


^1^H and ^19^F NMR of compound **20i**


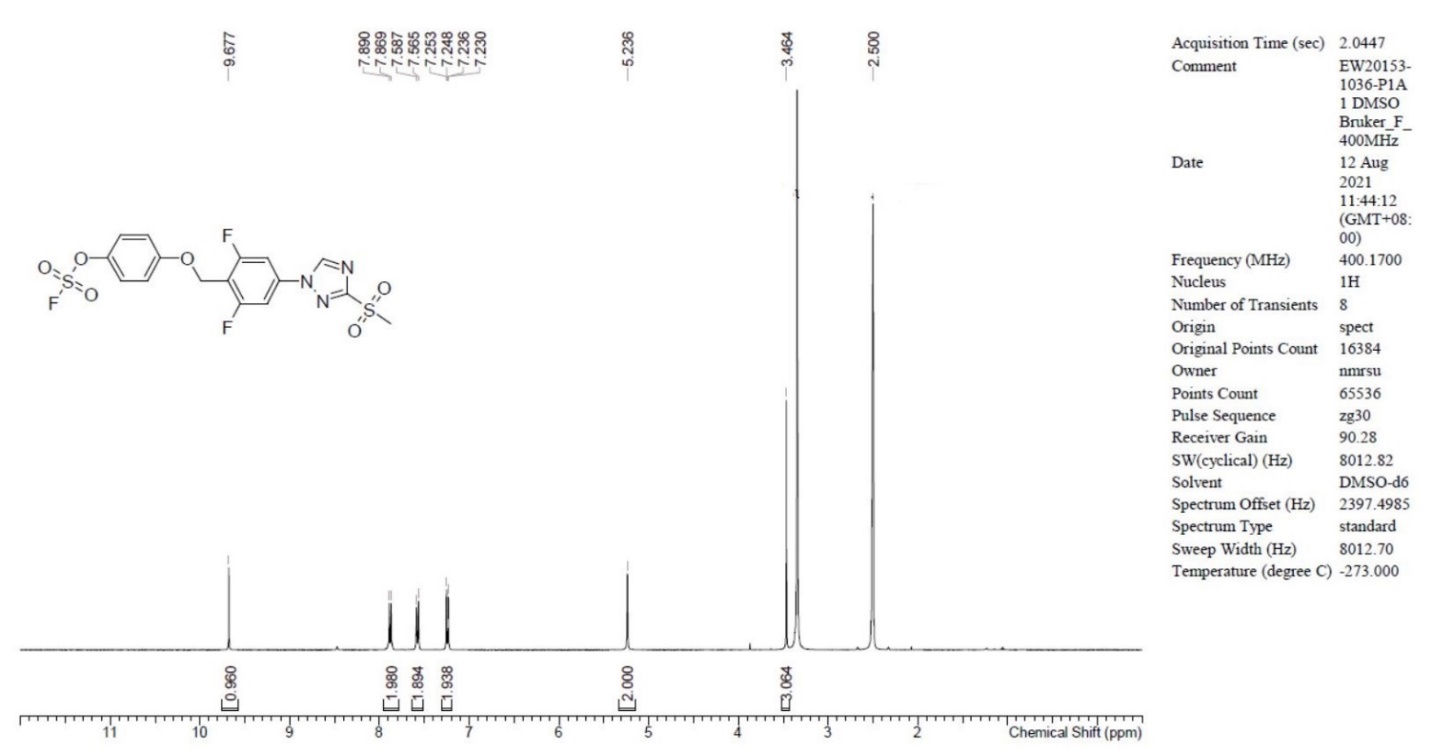


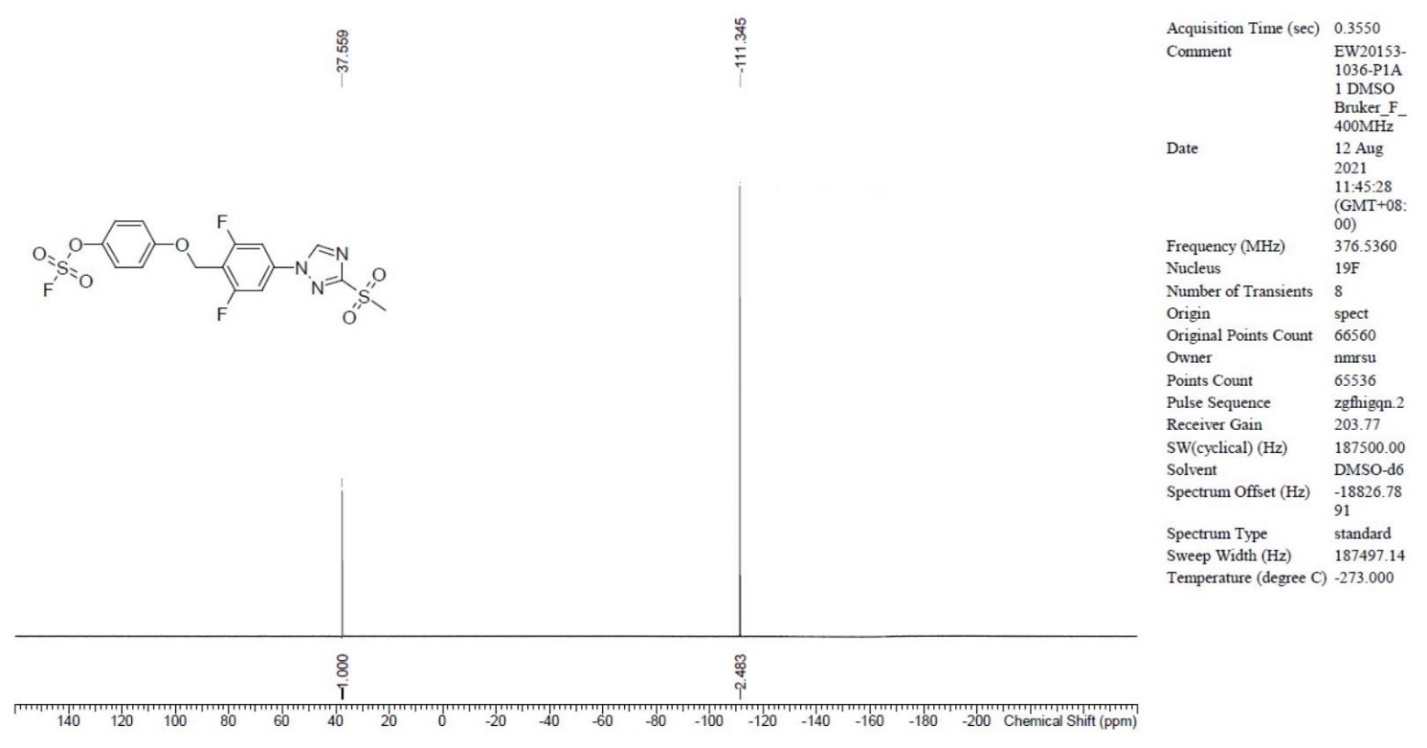


^1^H and ^19^F NMR of compound **20j**


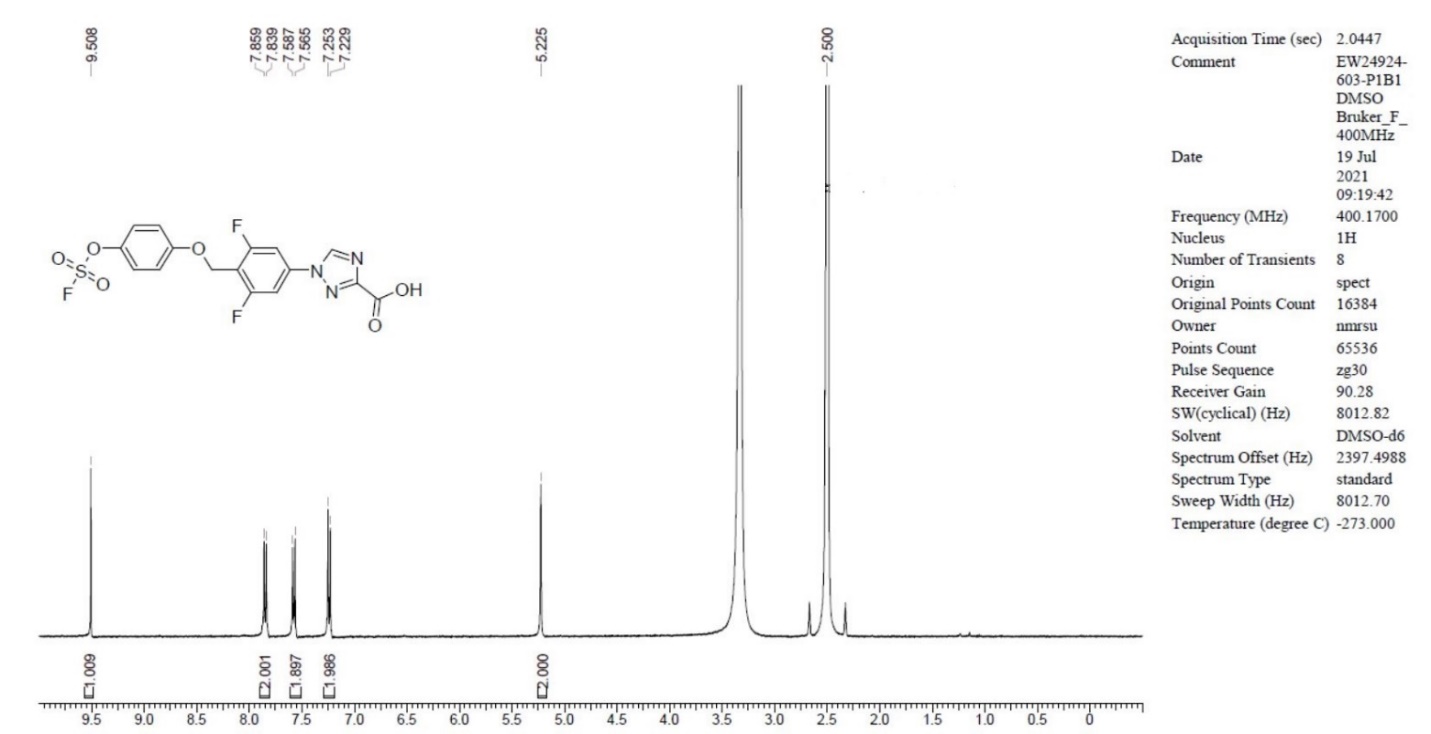


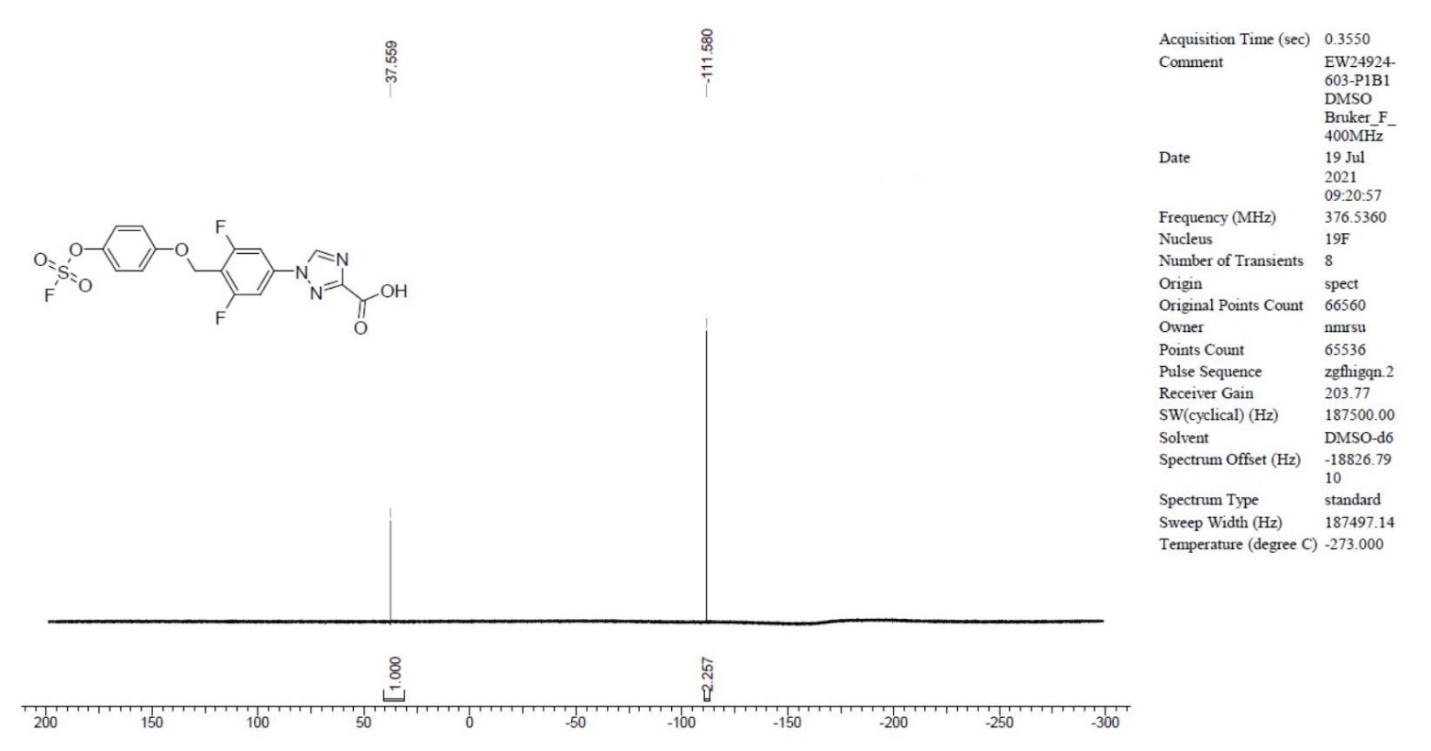


^1^H and ^19^F NMR of compound **20k**


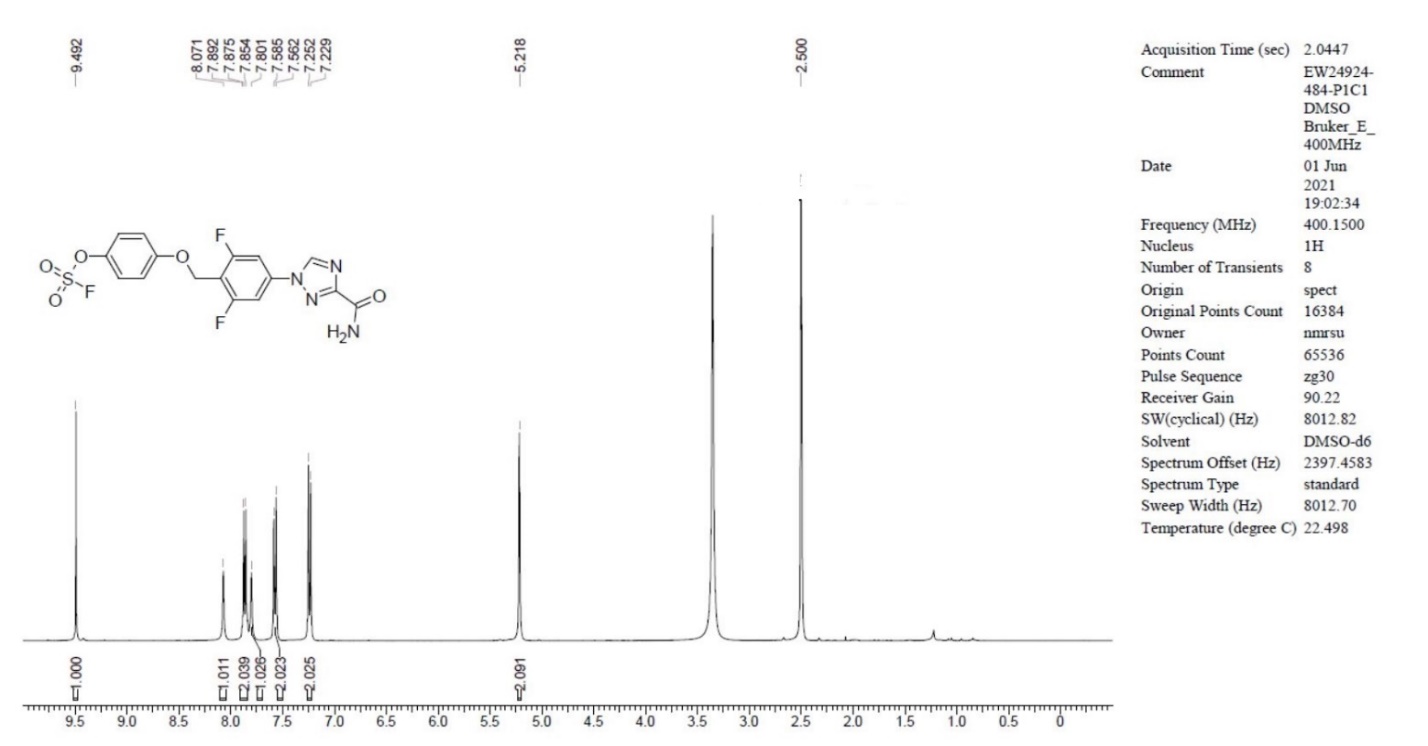


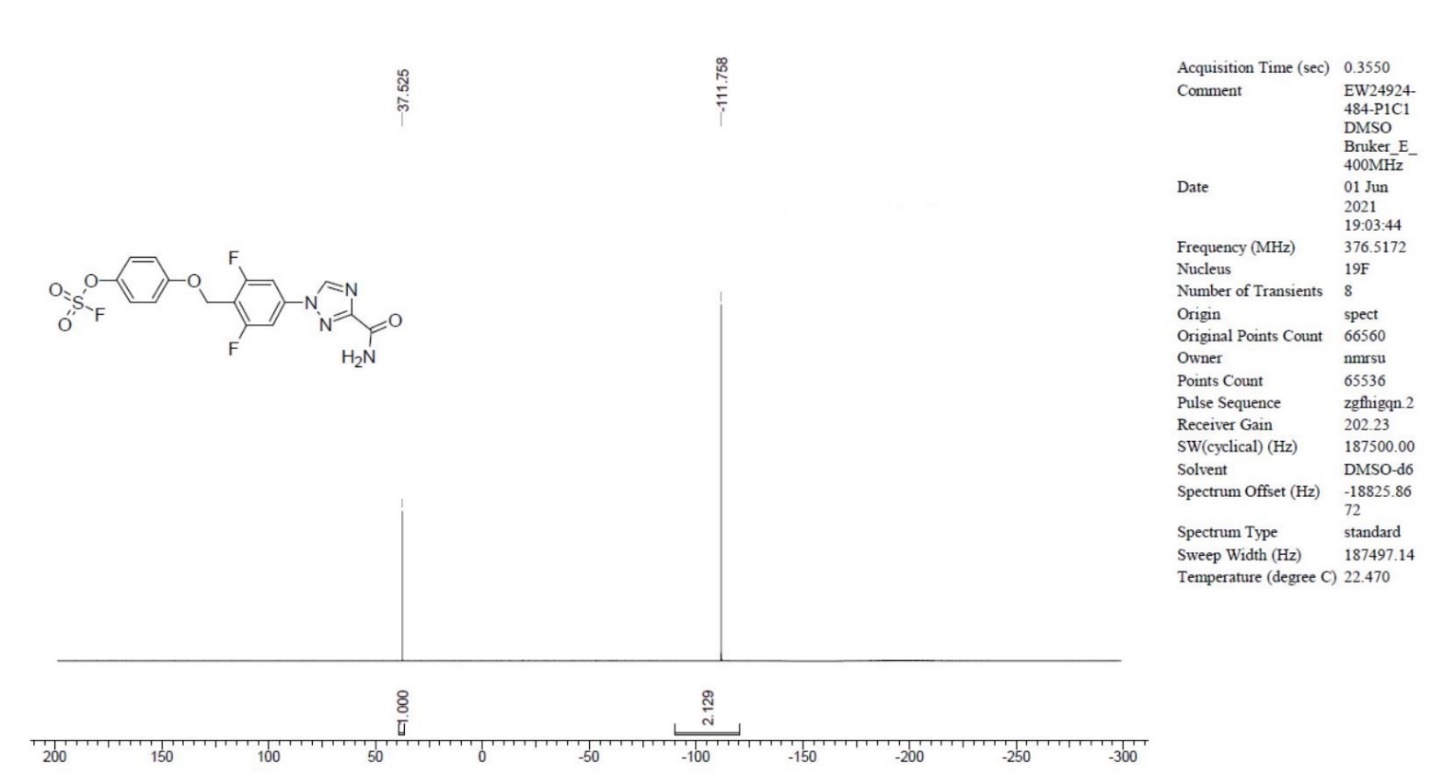


^1^H and ^19^F NMR of compound **21a**


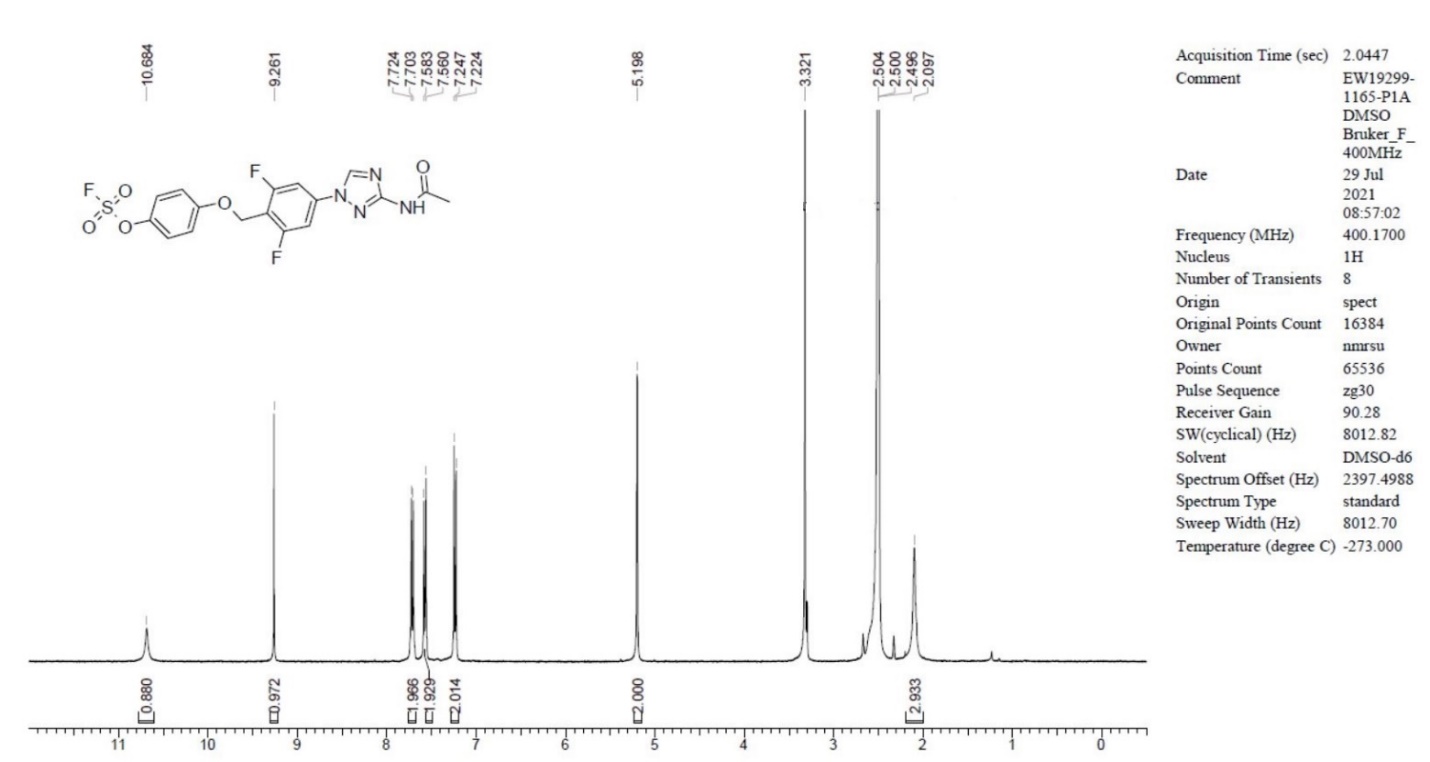


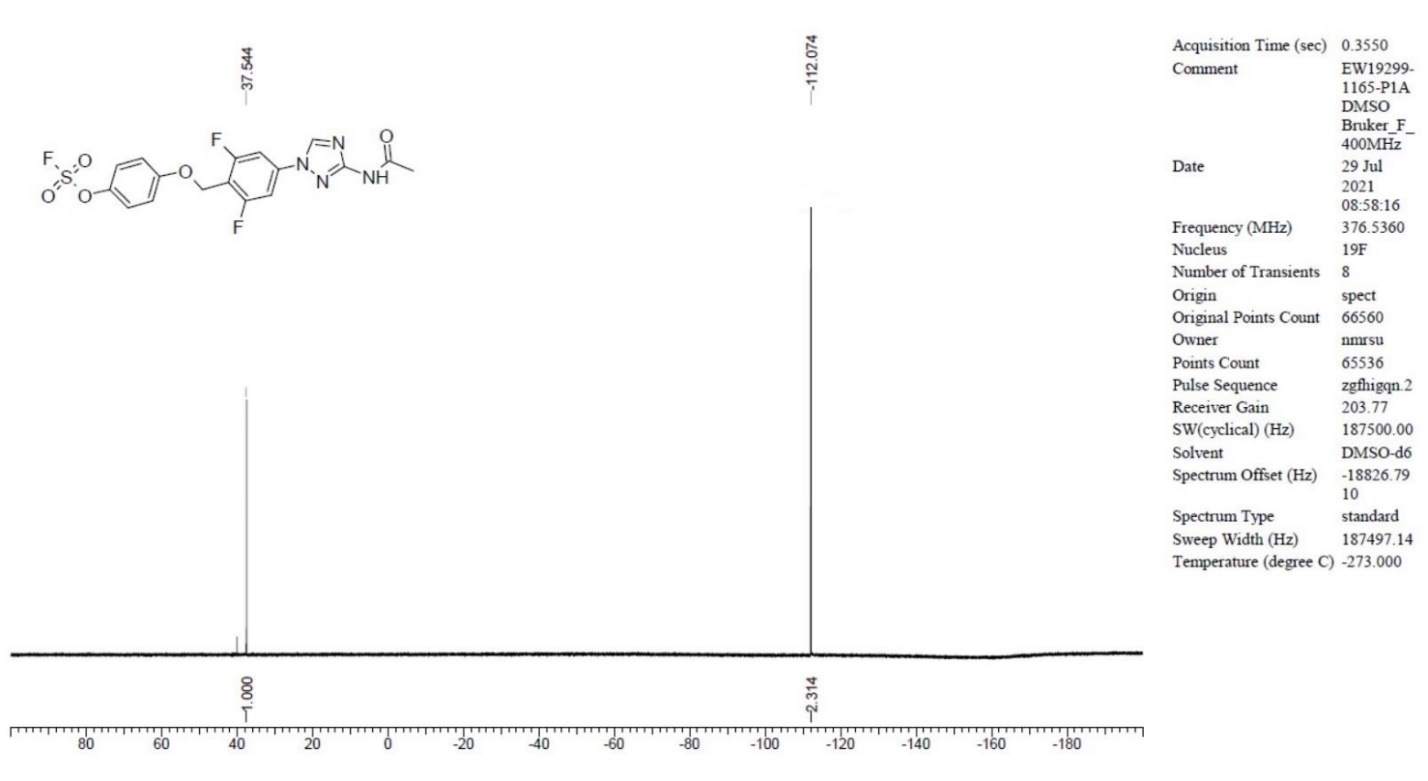


^1^H, 13C and ^19^F NMR of compound **21a**


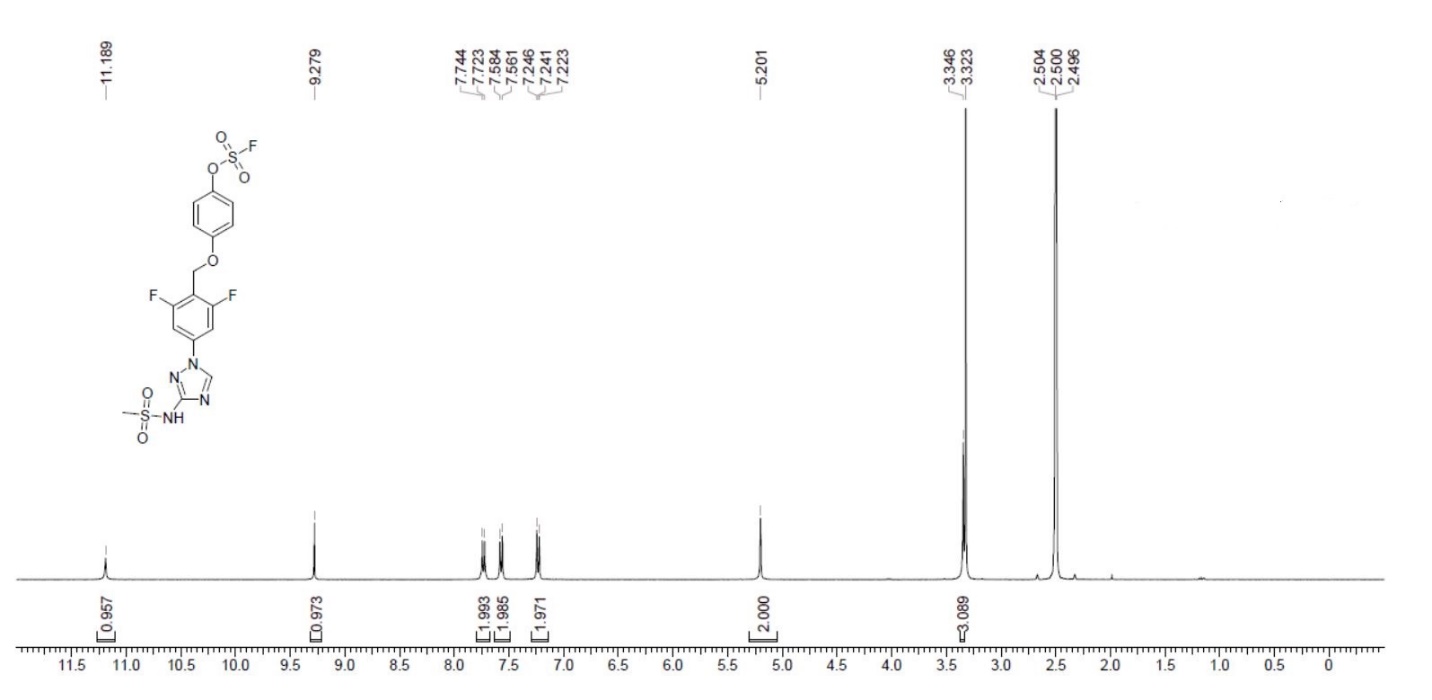


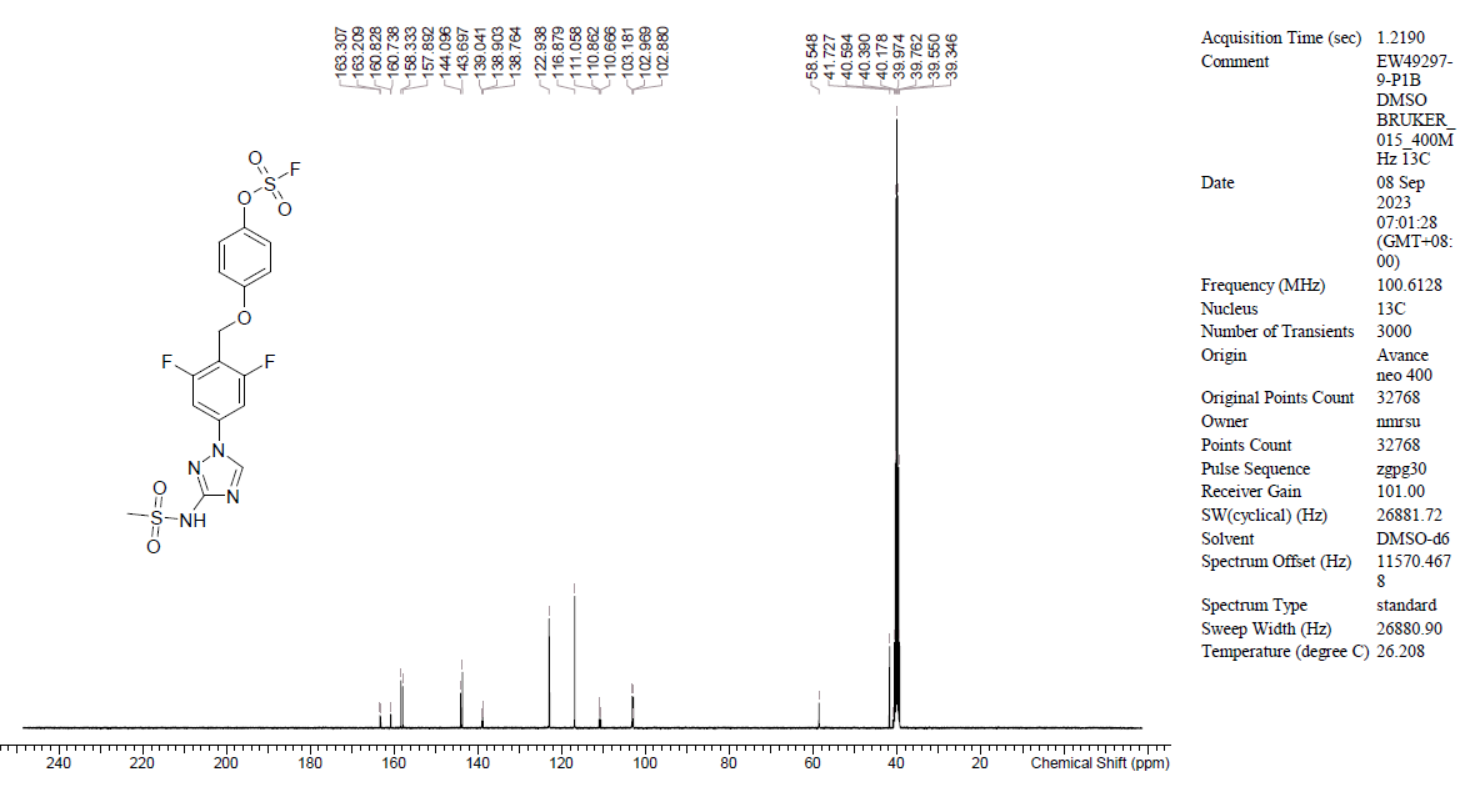


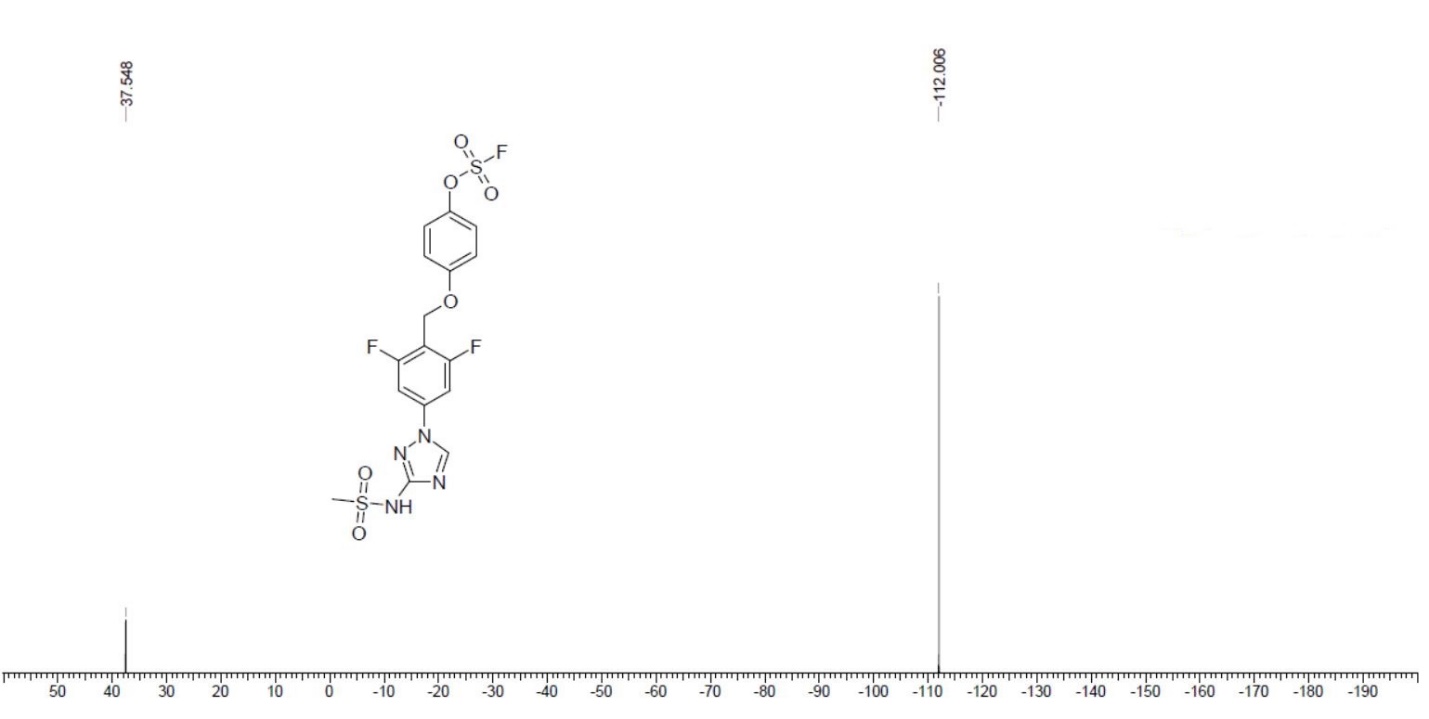


^1^H and ^19^F NMR of compound **20l**


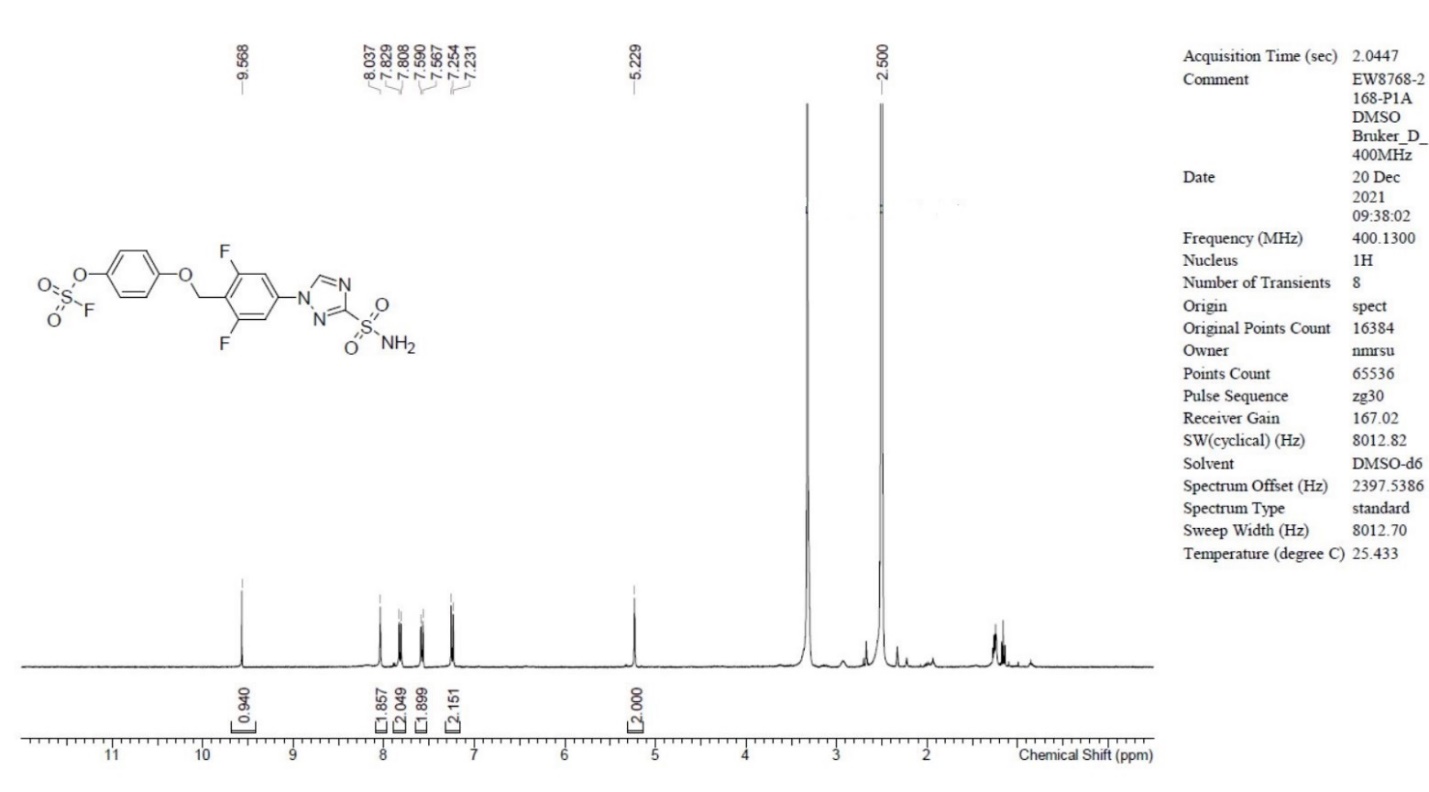


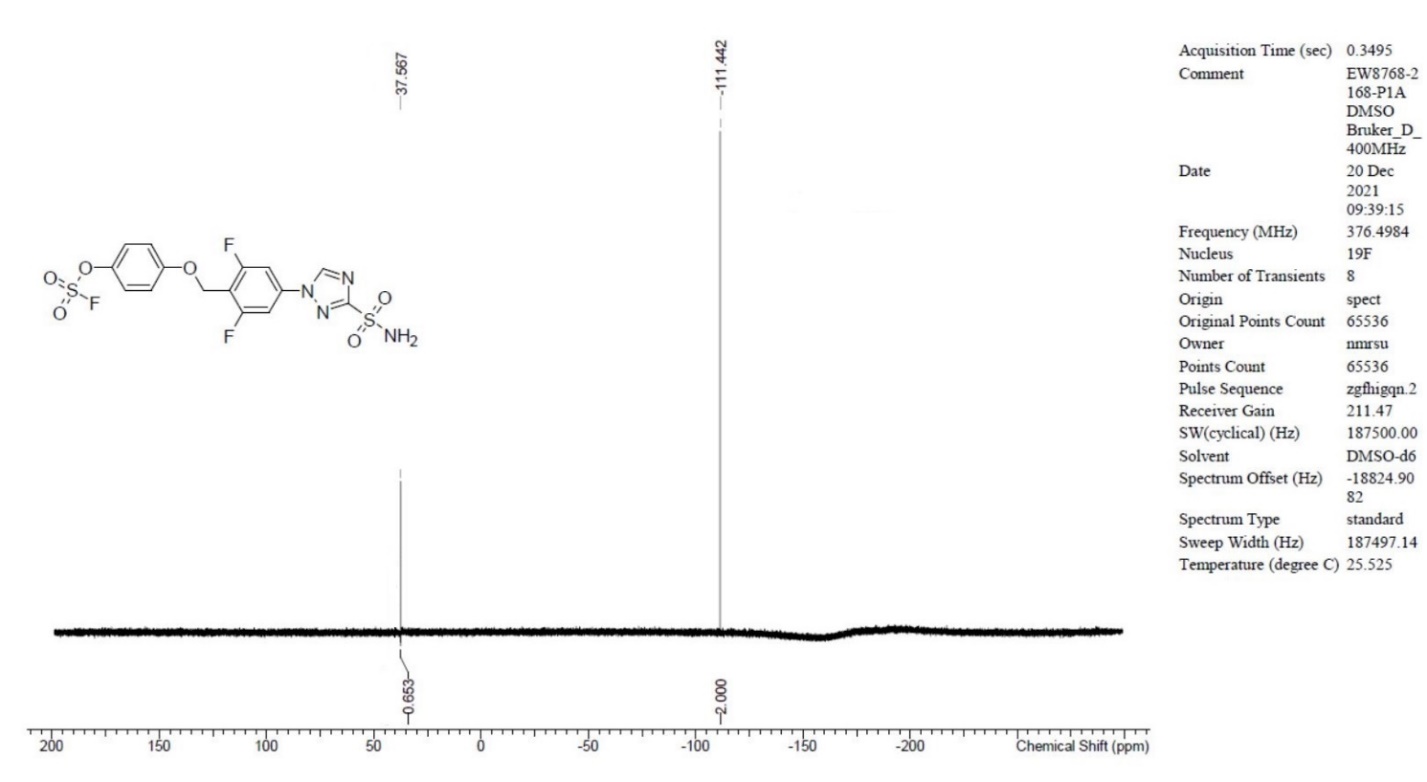


^1^H and ^19^F NMR of compound **21c**


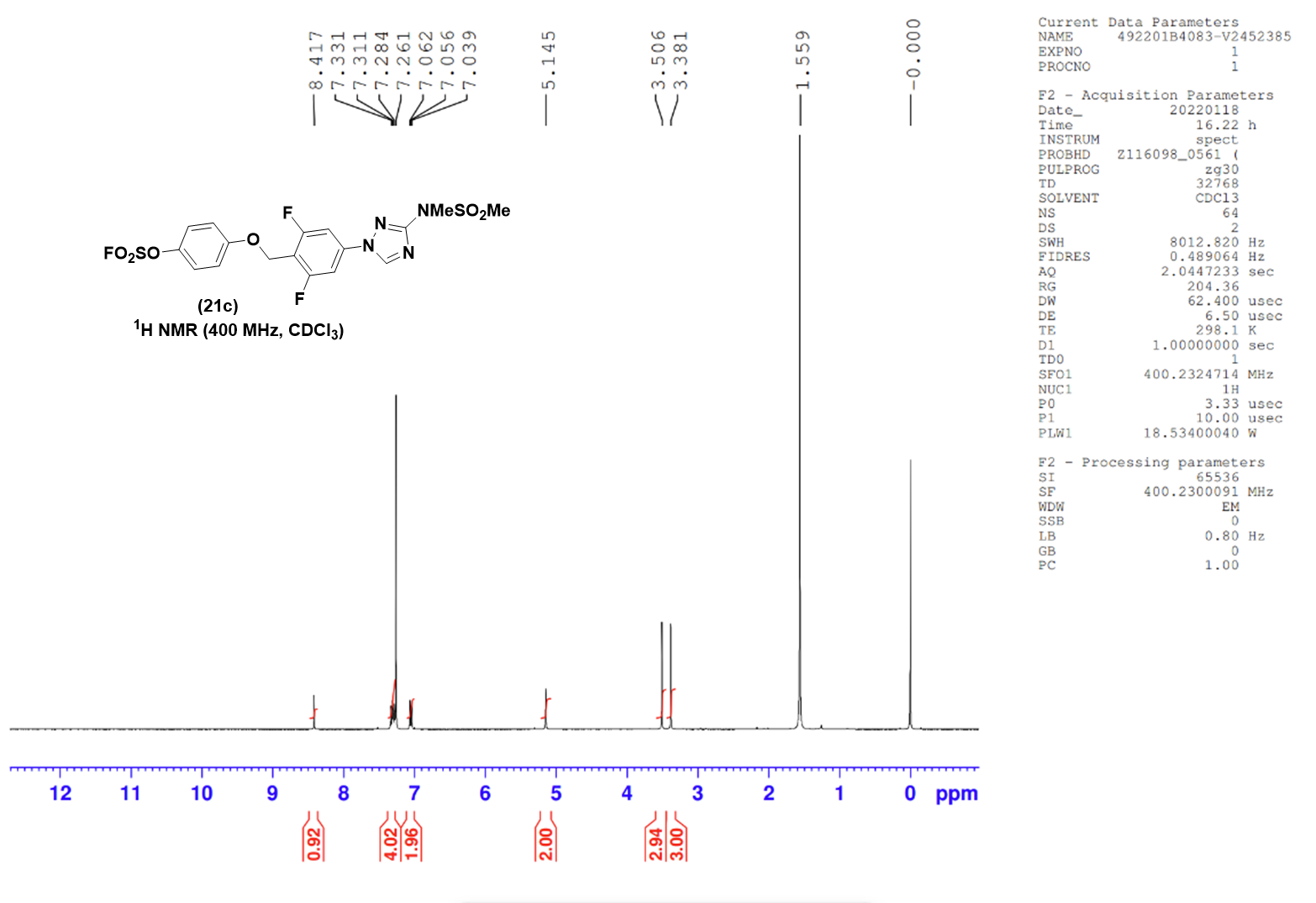


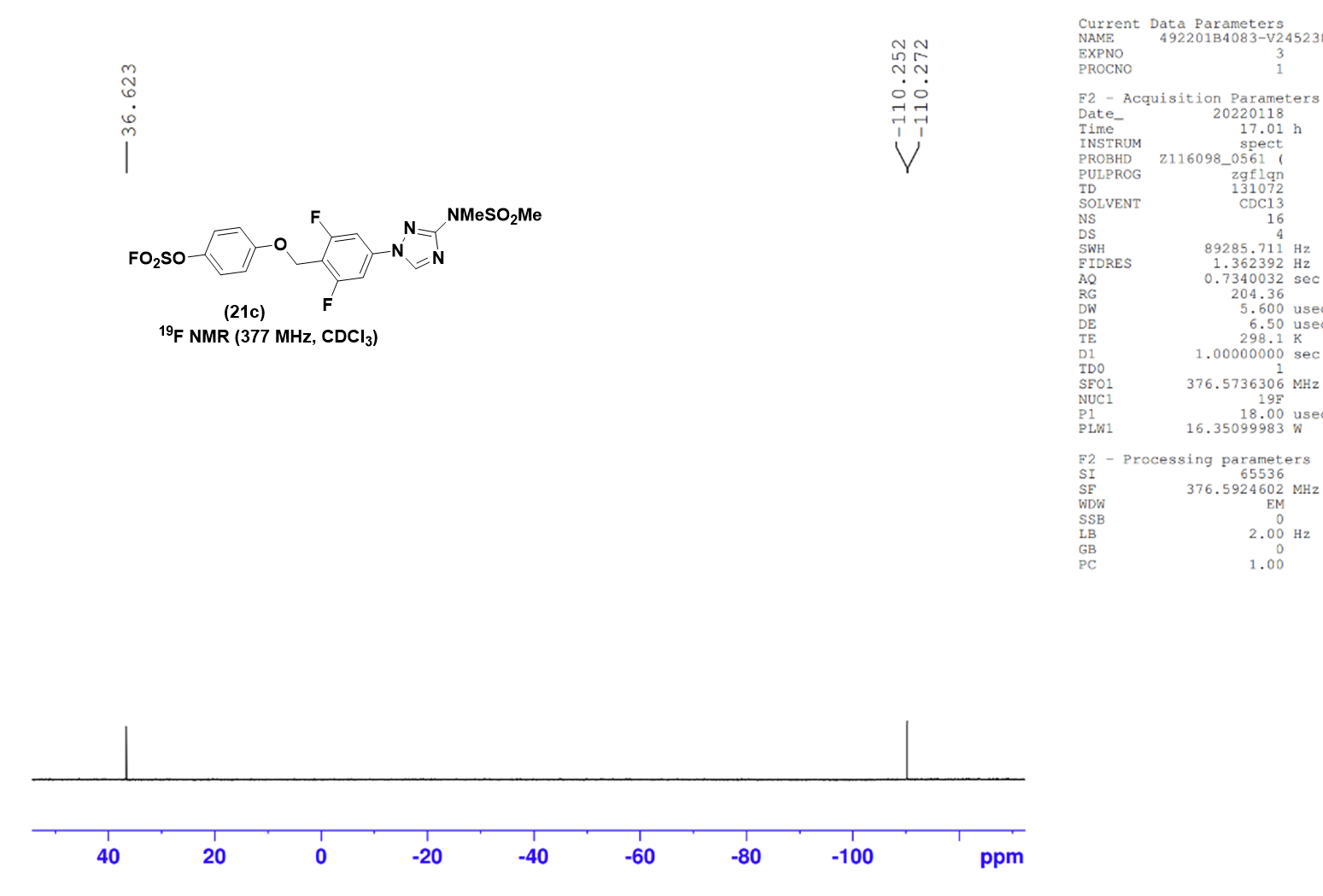


^1^H and ^19^F NMR of compound **21d**


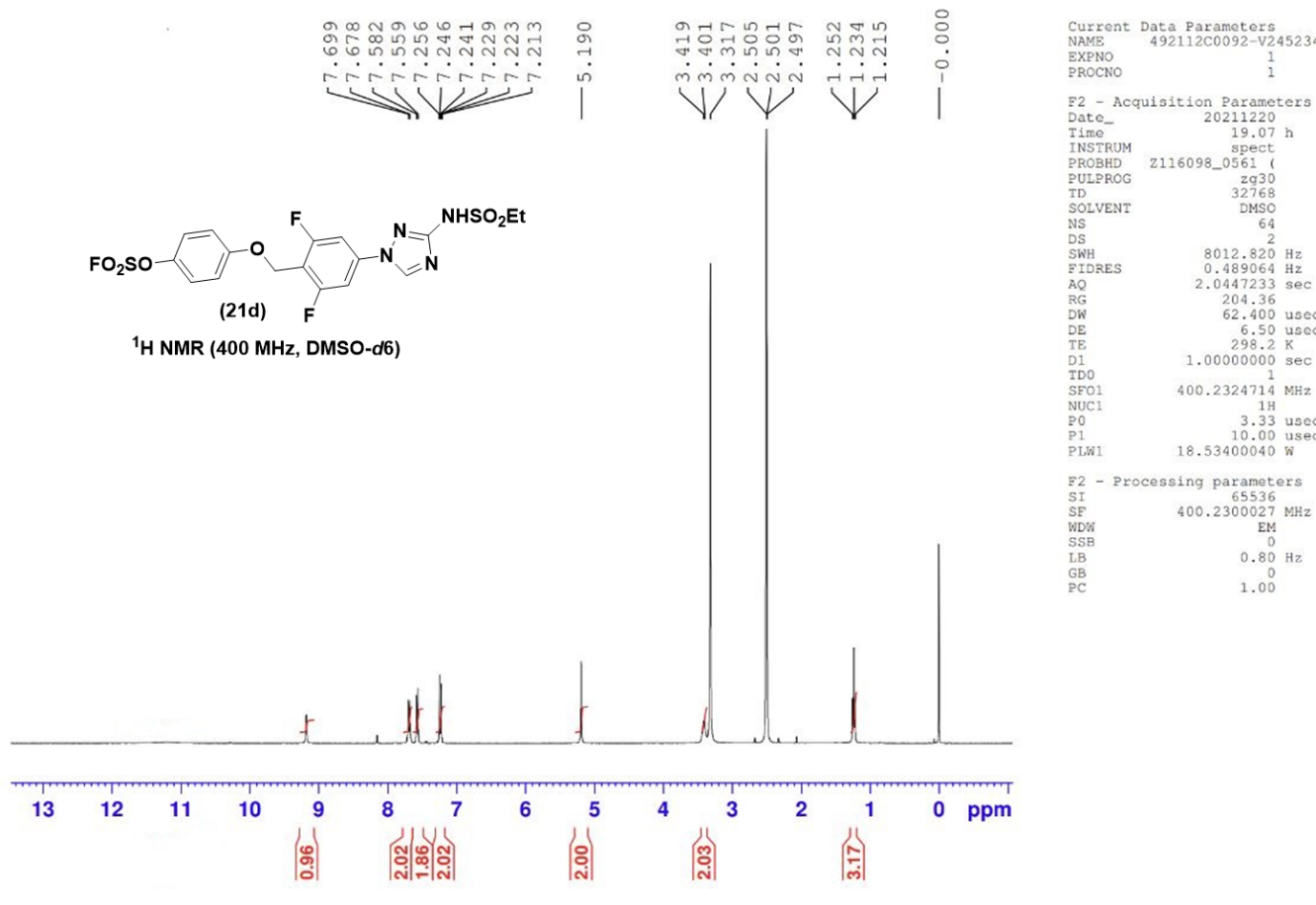


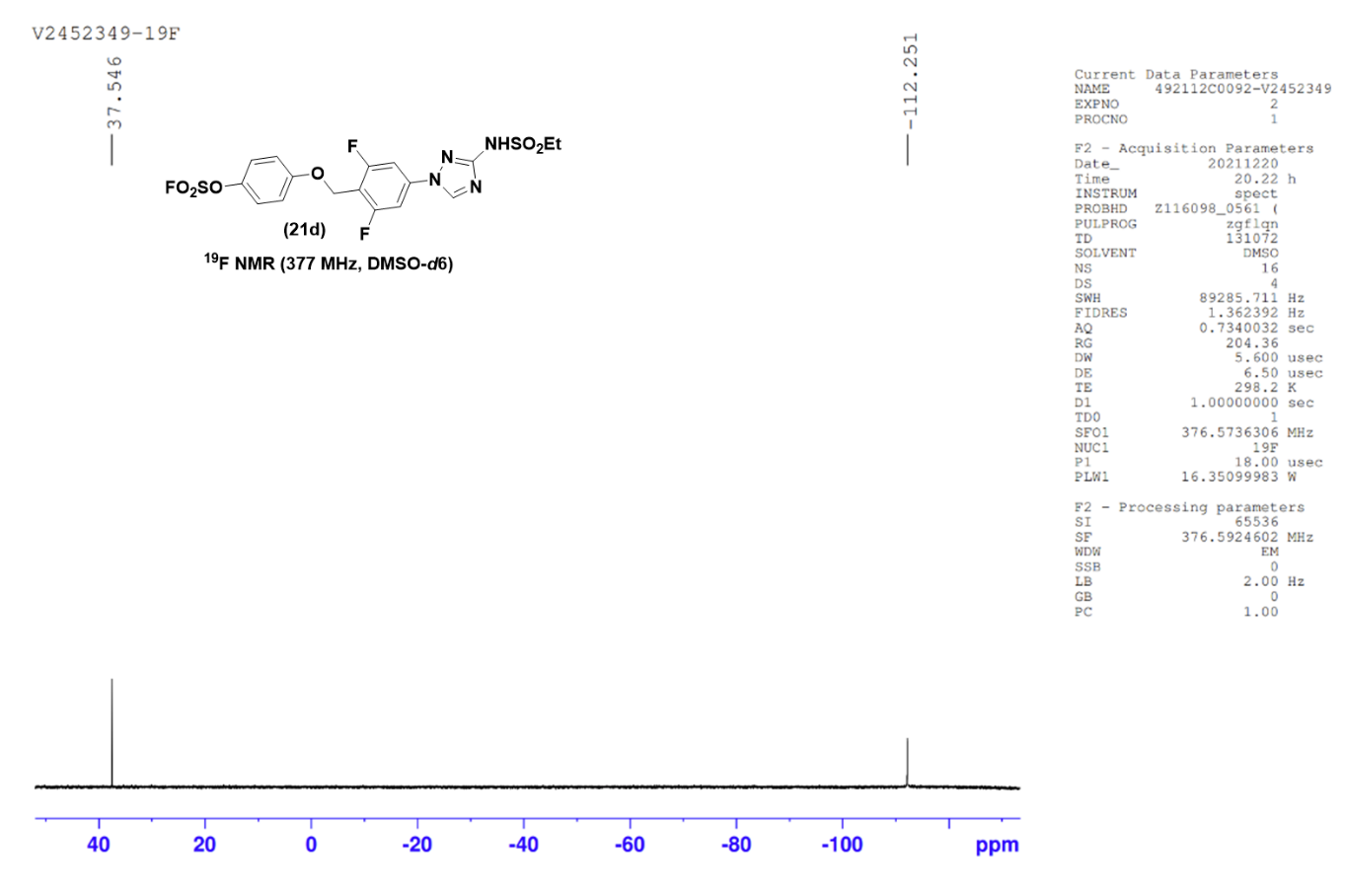


^1^H and ^19^F NMR of compound **21e**

**
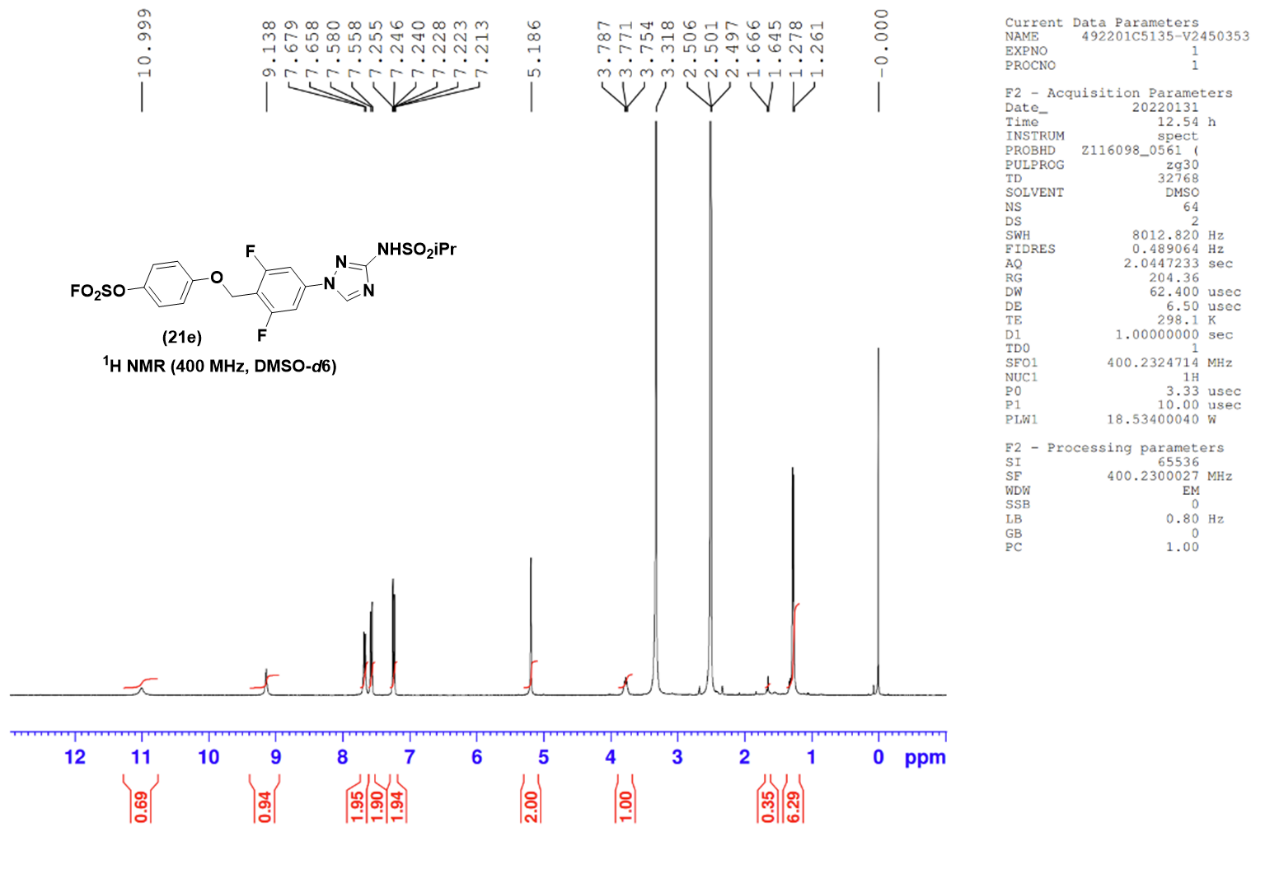
**

**
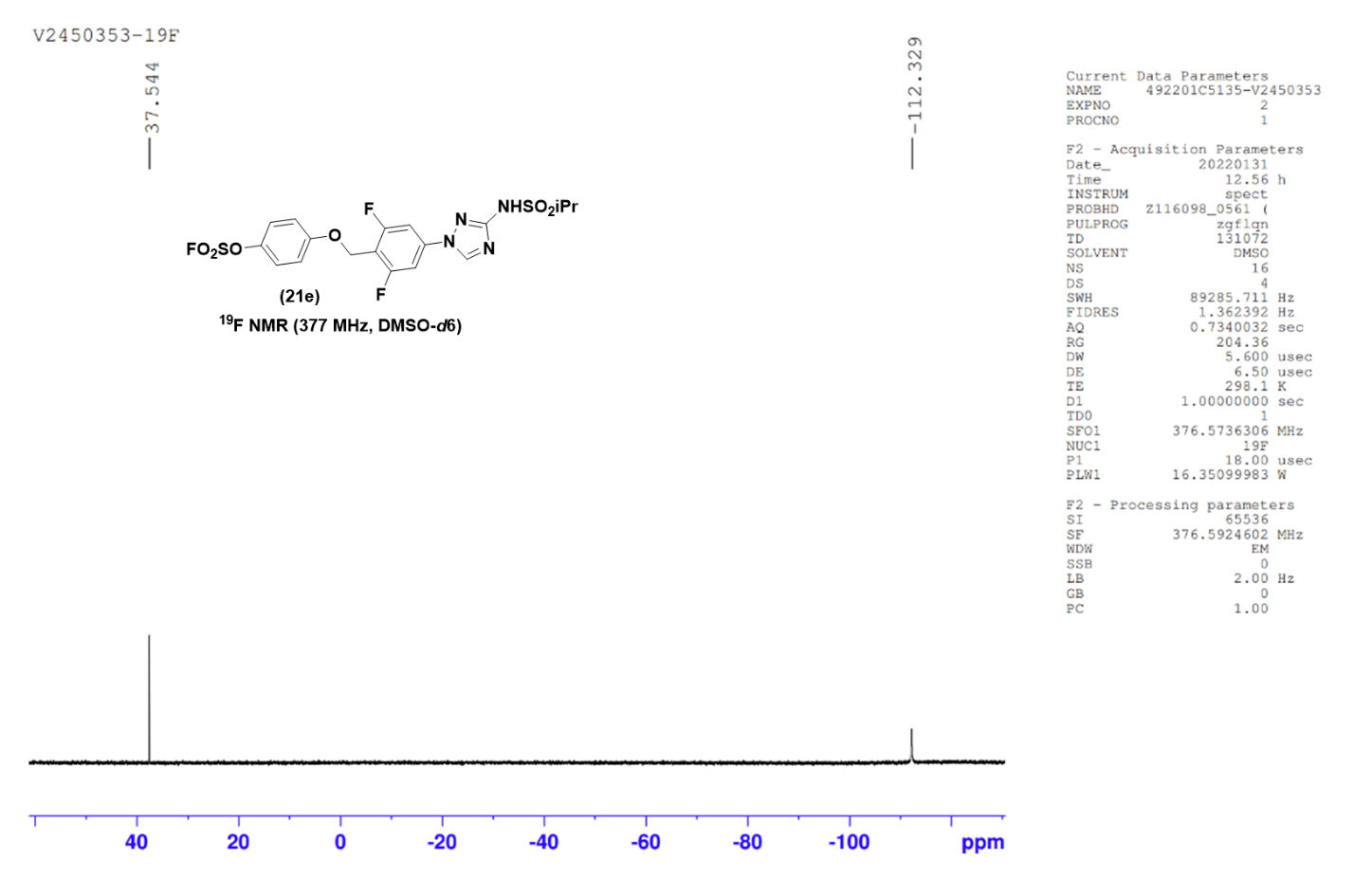
**

^1^H and ^19^F NMR of compound **21f**


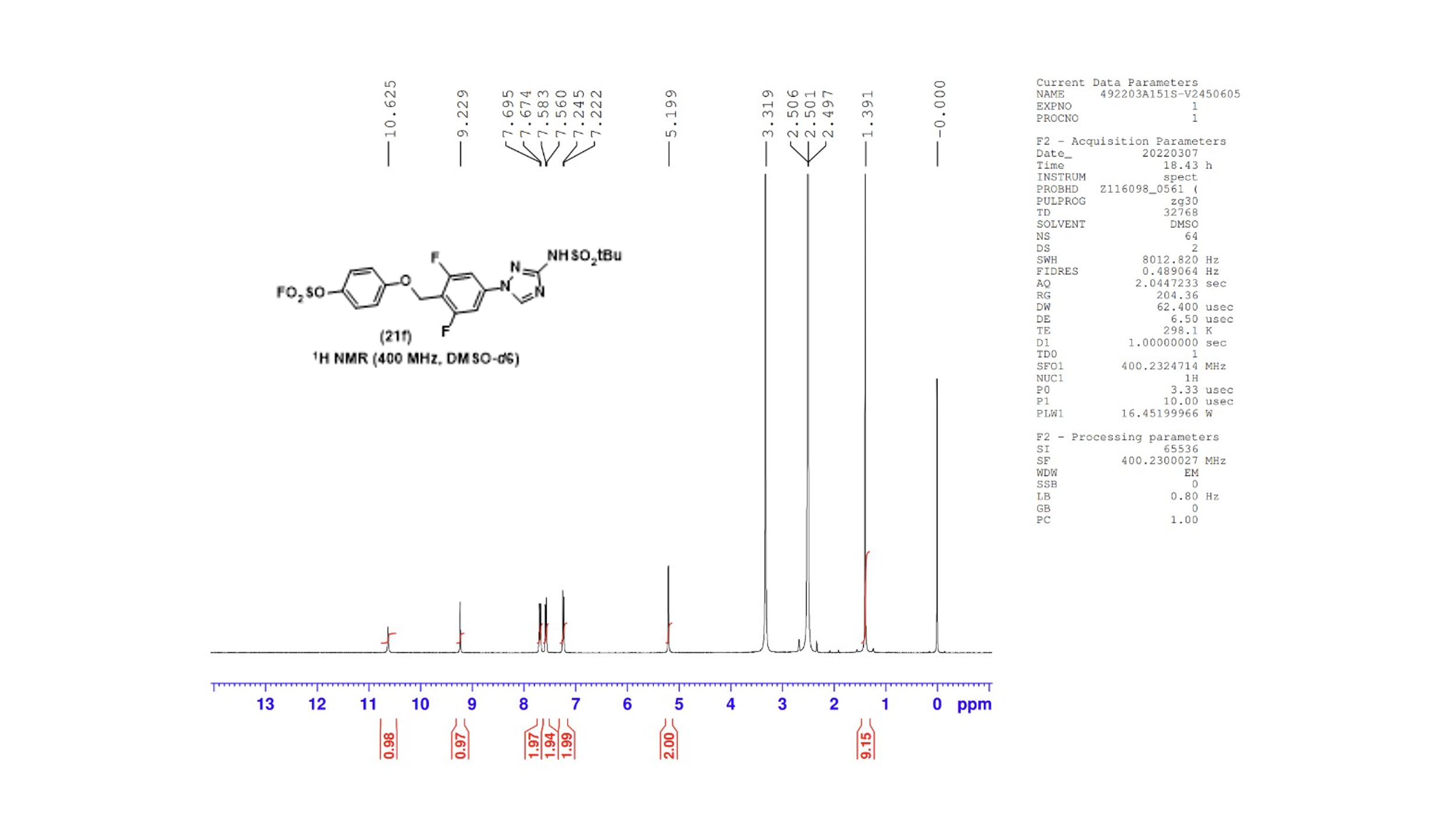


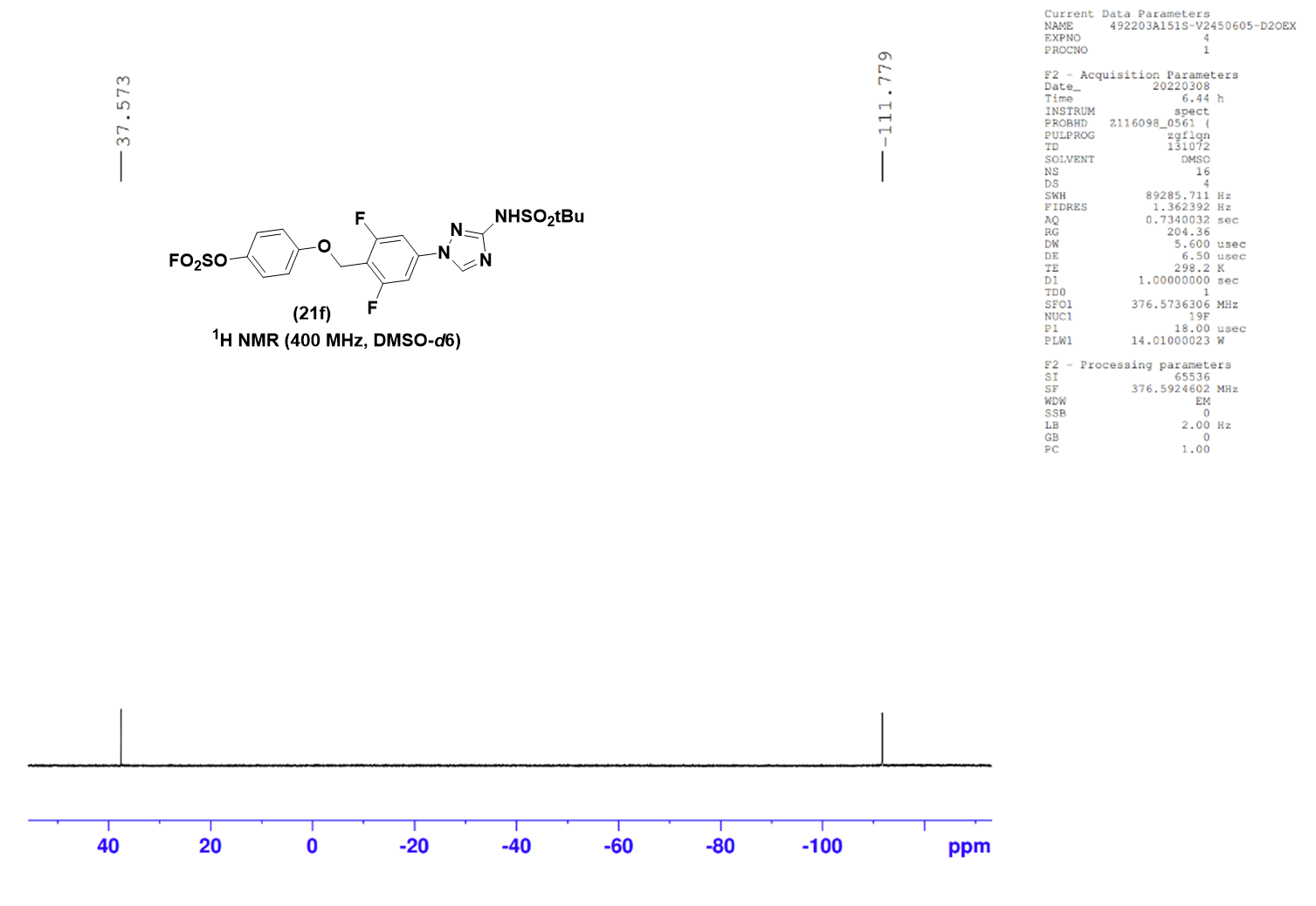

Supplement: Supplementary data 1 [file mmc1.docx]
